# Design and Synthesis of Novel Phenylahistin Derivatives Based on Co-Crystal Structures as Potent Microtubule Inhibitors for Anti-Cancer Therapy

**DOI:** 10.3390/md20120752

**Published:** 2022-11-29

**Authors:** Zhongpeng Ding, Feifei Li, Lianghui Xie, Minqing Gu, Chunlei Li, Chang Liu, Chao Peng, Wenbao Li

**Affiliations:** 1 Medical College, Linyi University, Shuangling Road, Linyi 276000, China; 2 Shenzhen BGI Marine Science & Technology Co., Ltd., Shenzhen 518002, China; 3 Shenzhen Huahong Marine Biomedical Co., Ltd., Shenzhen 518002, China; 4 College of Life Sciences, University of Chinese Academy of Sciences, Beijing 100049, China; 5 Innovation Center for Marine Drug Screening and Evaluation, Qingdao National Laboratory for Marine Science and Technology, Qingdao 266071, China

**Keywords:** phenylahistin, allyl-imidazole-type, SAR, microtubule inhibitor, anticancer

## Abstract

Phenylahistin is a naturally occurring marine product with a diketopiperazine structure that can bind to the colchicine site of microtubulin as a possible anticancer agent. To develop more potent microtubule inhibitors, novel phenylahistin derivatives were designed and synthesized based on the co-crystal complexes of phenylahistin derivatives and microtubulin. We established a focused library of imidazole-type molecules for the introduction of different groups to the C-ring and A-ring of phenylahistin. Structure–activity relationship studies indicated that appropriate hydrocarbon substituents and unsaturated alkenyl substituents at the 1-position of the imidazole group are important for improving the activity of such compounds. In addition, this study found that propylamine groups could maintain the activity of these compounds, as exemplified by compound **16d** (IC_50_ = 5.38 nM, NCI-H460). Compound **15p** (IC_50_ = 1.03 nM, NCI-H460) with an allyl group exhibited potent cytotoxic activity at the nanomolar level against human lung cancer cell lines. Immunofluorescence assay indicated that compound **15p** could efficiently inhibited microtubule polymerization and induced a high expression of caspase-3. **15p** also displayed good pharmacokinetic characteristics in vitro. Additionally, the growth of H22 transplanted tumors was significantly inhibited in BALB/c mice when **15p** alone was administered at 4 mg/kg, and the tumor inhibition rate was as much as 65%. Importantly, the continuous administration of **15p** resulted in a lower toxicity than that of docetaxel (10 mg/kg) and cyclophosphamide (20 mg/kg). Overall, the novel allyl-imidazole-diketopiperazine-type derivatives could be considered safe and effective potential agents for cancer treatment.

## 1. Introduction

According to the World Health Organization (WHO) estimates for 2019, cancer was either the first or second leading cause of death of those aged under 70 in 112 out of 183 countries [1]. Chemotherapeutic drugs are an important component of commonly used drug-treatment regimens and play an indispensable role in cancer treatment. Tubulin inhibitors are significant types of chemotherapeutic drugs, since microtubules play an essential role in multiple cellular functions [2,3]. There are six tubulin target sites for prospective anticancer agents: maytansine, vinca, laulimalide, taxane, colchicine, and pironetin. Several microtubule-targeting agents, including vinca alkaloids and taxanes, are frequently used for the treatment of various cancer types. However, colchicine-type tubulin inhibitors have yet to be developed as anti-tumor drugs [4,5].

**Figure 1:**
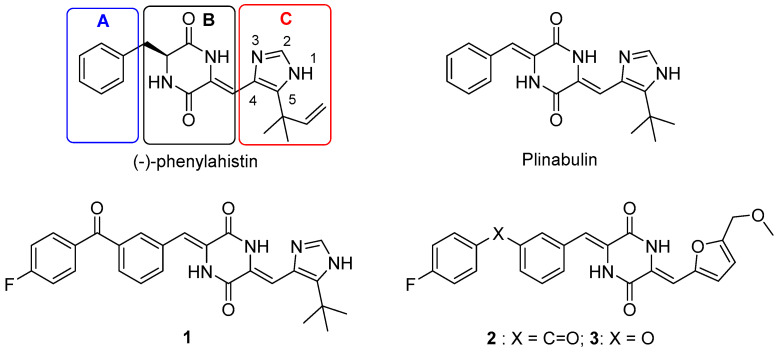
Chemical structures of (-)-phenylahistin, plinabulin and its representative derivatives.

Phenylahistin is a diketopiperazine structure produced by the marine fungus Aspergillus sp., and phenylahistin is a tubulin depolymerization agent that targets the colchicine site [6,7]. Plinabulin (NPI-2358), obtained by the structural modification of phenylahistin, can directly act on tumor cells by inhibiting tubulin polymerization and blocking microtubule formation, thus inducing cell death [8,9,10] (Figure 1). Furthermore, plinabulin can also destroy the blood vessels surrounding the tumor to block the nutrient supply to the cancer cells. In addition, plinabulin can induce the release of GEF-H1, which triggers signaling programs to increase neutrophils [11,12]. The candidate drug is being developed by Beyond Spring Pharmaceuticals and a New Drug Application (NDA) has been submitted in the United States and China for plinabulin’s use in the treatment of non-small cell lung cancer (NSCLC) and chemotherapy-induced neutropenia (CIN). However, plinabulin has low efficacy in vivo when used alone as an anticancer agent. It was used in combination with docetaxel during clinical trials for NSCLC. Therefore, the development of novel phenylahistin derivatives that display greater efficacy can be of great significance in improving cancer treatment.

**Figure 2:**
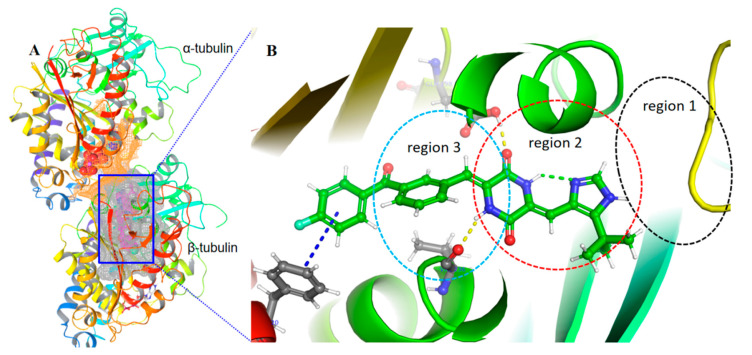
The complex structure of compound 1 and tubulin. (**A**) Binding area and interaction force. (**B**) Co-crystal structure of tubulin with compound 1 in different visual displays.

Recently, the crystal structures of various phenylahistin derivatives have been resolved and analyzed, namely Plinabulin, (PDB: 5C8Y, 6S8K, 6S8L) [12,13], KPU-105 (PDB: 5YL4) [14], MBRI-001 (PDB: 5XI5) [15], and compound **1** (PDB: 5XHC)). The co-crystal complex of compound **1** (Figure 2) with a resolution of 2.75 Å has been deposited in the protein structure database (PDB), which provided a new understanding for the interaction of tubulin with phenylahistin derivatives [16]. In this study, Maestro software was used to further analyze the crystal structures of phenylahistin derivatives. The results showed that phenylahistin and its derivatives were located in the deeper positions of β-tubulin and mainly bound to regions 2 and 3 of the colchicine site. The binding pocket of phenylahistin and its derivatives crosses the α/β interface and extends to the boundary of the GTP pocket formed by hydrophilic and hydrophobic amino acid residues. To obtain more potent phenylahistin derivatives, this study entailed a further analysis of the co-crystal structure of compound **1** with tubulin and, furthermore, the design and modification of the C-ring [16,17].

## 2. Results and Discussion

### 2.1. Design Strategy 

**Figure 3:**
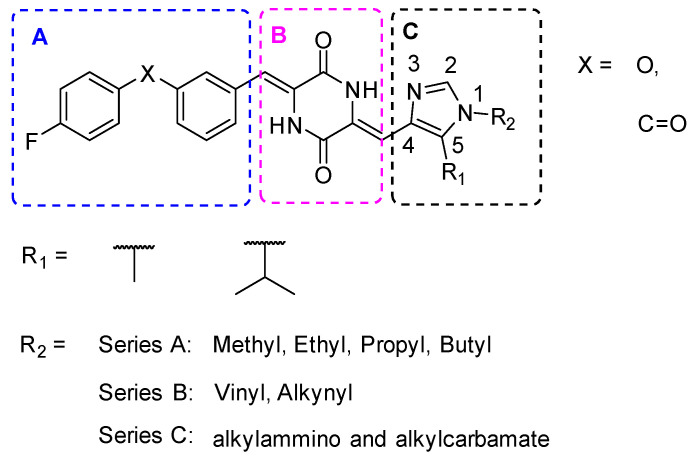
General design structures of compounds **15a**–**15q** and compounds **16a**–**16d**.

The co-crystal complex of compound **1** with tubulin provided new insights into the interaction between the microtubule and the molecule [17]. In the crystal structure of 5XHC, the benzoyl derivatives induced a new binding pocket in region 3 (induced-fit theory), and enabled the benzene ring of the benzoyl group and the benzene ring of the amino acid residue PHE20 to generate stacking interaction. The binding pocket of the phenylahistin derivative crosses the α/β interface and extends to the boundary of the GTP pocket, providing a new space for bonding. Region 1 of this pocket was not occupied by the phenylahistin derivative, and the molecule did not form an interaction force with the protein amino acid residues. the p-fluorobenzoylphenyl or p-fluorophenoxyphenyl groups were preferred as pharmacophores of A-ring, and the diketopiperazine core structure of the B-ring was unchanged to retain their favorable interactions [17]. In this study, we further designed three series of compounds with different substitutions of the C-ring to explore the possibility for more active phenylahistin derivatives. Postion 1 or 5 of the imidazole was substituted by different groups, and different types of groups at the C-ring of the series A/B/C were searched in order to design compounds **15a**–**15p** and compounds **16a**–**16d** (Figure 3).

### 2.2. Chemistry

To synthesize the phenylahistin derivatives **15a**–**15q** and **16a**–**16d**, we explored and adopted three synthetic strategies to couple the imidazole moiety and R_2_ groups. The preferred routes could then be used for different bases (For example, NaH, Cs_2_CO_3_, K_2_CO_3_) to obtain key intermediates, taking into account the optimization for higher yield and fewer by-products. The chemical structures of these compounds were characterized by nuclear magnetic resonance (NMR) and high-resolution mass spectrometry (HRMS) analysis (Figures S1–S86, Supplementary Materials).

#### 2.2.1. Synthesis of Intermediates **11a**–**11c**

**Scheme 1:**
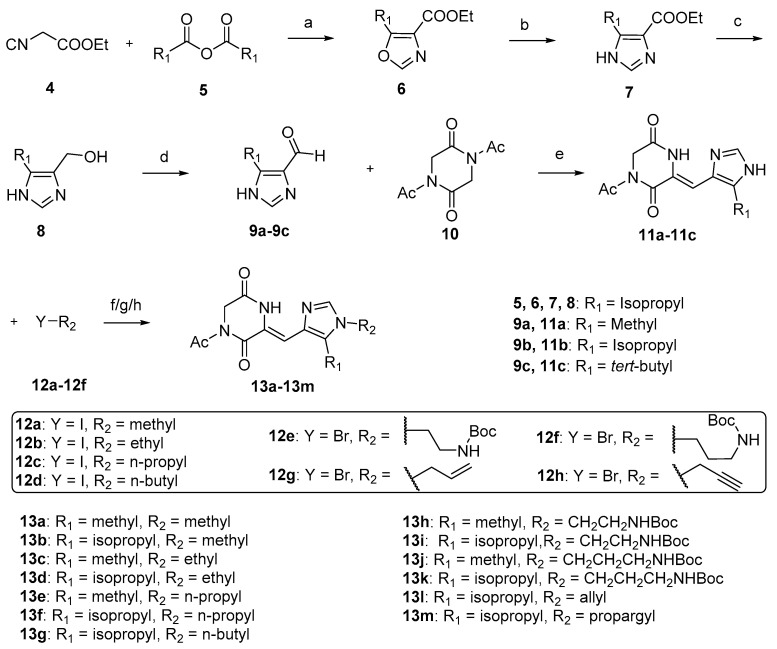
Synthesis of intermediates **13a**–**13k**. Regents and conditions: (**a**) DBU, THF, rt, 48 h, 95%; (**b**) formamide, 175 °C, 36 h, 60%; (**c**) LiAlH_4_, THF, 0 °C to rt, 6 h, 99%; (**d**) MnO_2_, DCM, rt, 48 h, 61%; (**e**) Cs_2_CO_3_, Na_2_SO_4_, DMF, rt, 20 h, 36–47%; (**f**) NaH, DMF, −30 °C –rt, 1–2 h, 45–73%; (**g**) Cs_2_CO_3_, (NaI), DMF, rt–70 °C, 2 h, 43–80%; (**h**) K_2_CO_3_, DMF, 45 °C, 2 h, 59–68%.

The phenylahistin derivatives **15a**–**15q** were synthesized via a sequence of seven linear synthesis. Firstly, compound **6** was synthesized through a [3 + 2] cyclization reaction using ethyl isocyanoacetate and isobutyric anhydride as starting materials in the presence of 1, 8-diazabicyclo[5.4.0]undec-7-ene (DBU) for 48 h at room temperature, and then purified by silica gel column chromatography. The oxazole ester was converted into the imidazole ester by the solvolysis reaction in formamide for 24 h at 175 °C, followed by slurry purification using water. The isopropyl imidazole ester was then reduced to alcohol with LiAlH_4_. Subsequently, the isopropyl aldehyde **9b** was produced by an oxidation reaction using MnO_2_. 5-Methylimidazole-4-carbaldehyde (**9a**) and 5-tert-butylimidazole-4-carbaldehyde (**9c**) were purchased from commercial suppliers (Scheme 1). Then, a tandem aldol condensation of two different aldehydes onto the diacetyl-2,5-piperazinedione ring was carried out in the presence of Cs_2_CO_3_ in N,N-dimethylformamide (DMF) to obtain the target product.

**Figure 4:**
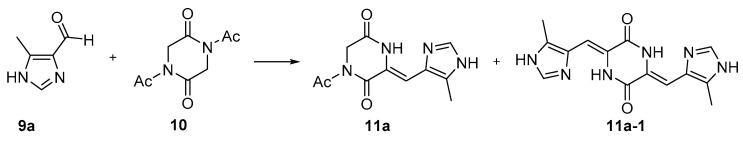
Reaction formula of compounds **11a** and **11a**-**1**.

The imidazole aldehydes were condensed with diacetyl-2,5-piperazinedione for 20 h at room temperature. When the alkyl group was isopropyl, the diacetyl-2,5-piperazinedione **10,** only monocondensation product was achieved. However, the reaction can produce double imidazol-3,6-yl piperazine-2,5-dione (**11a**-**1**) as a by-product because of the small steric hindrance of the methyl group at the 5-position of the imidazole (see Figure 4). The by-product of the unsubstituted imidazole was greater than that of methyl imidazole, and was difficult to purify by silica gel column chromatography. Therefore, the results indicated that the bulky groups at the 5-position were important in preventing the formation of by-products. 

#### 2.2.2. Synthesis of Intermediates **13a**–**13m**

Compounds **1** has three active hydrogen reaction centers on the nitrogen atoms. Three synthetic strategies were, therefore, explored in order to obtain single substituted compounds. (1) Strategy 1 [18]: Compound **1** is directly reacted to the starting materials containing halogenated; (2) Strategy 2: If the reaction selectivity of strategy 1 is poor, **11a** or **11b** would be used as a starting material to react with the halogenated group; (3) Strategy 3: if the alkylation reaction involves the NH group of the 2,5-DKP, then **9a** or **9b** would be used to react with halogenated hydrocarbons. After several experimental explorations, it was discovered that the substituents in Strategy 1 could react with multiple nitrogen atom centers, and different proportions of hydrocarbyl substitution products were produced.

**Figure 5:**
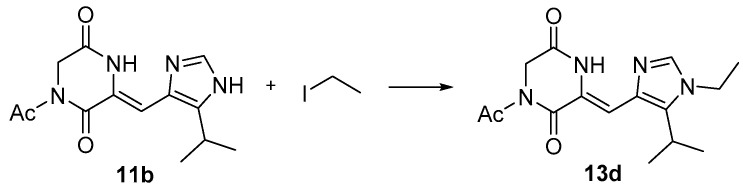
Synthesis of intermediate **13d**.

According to Strategy 2 (Figure 5), **11b** was reacted with iodoethane using NaH at −30 °C as per the method reported in the literature [18]. However, **11b** raw material remained; therefore, different reaction conditions were re-explored, as shown in Table 1. When NaH was used as the base, the conversion rate was only approximately 40%, and changes in the temperature had little effect on the reaction. Furthermore, altering the order in which materials were added did not positively influence the conversion rate. When DIPEA, TEA, and Cs_2_CO_3_ were used as bases, the unconverted amount of **11b** were 82%, 91%, and 0%, respectively. Therefore, the reaction system consisting of Cs_2_CO_3_ as the base and DMF at room temperature was preferable. Experiments verified that this condition was also suitable for the reaction of other iodohydrocarbyl groups with **11a** or **11b** to obtain key intermediates **13a**–**13k** with better yield and higher purity.

**Figure 6:**
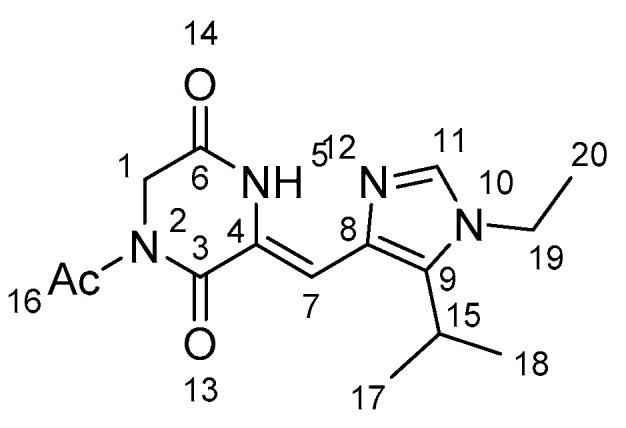
The structural formula and atom number of compound **13d**.

The structure of the key intermediate **13d** was confirmed by HNMR, CNMR, two-dimensional NMR, and mass spectrometry (Figure 6). The molecular formula of compound **13d** was determined as C_15_H_20_N_4_O_3_ and the exact mass [M + H]^+^ was 305.1608. The purity achieved was over 99% by LC-MS analysis. The MS data obtained were 304.82 by Qd assay, which was preliminarily identified as a monoethyl-substituted intermediate.

NMR spectra characterizations of the key intermediate **13b** are shown in the Supporting Information. The chemical shifts in H-11, H-17 and H-18, H-19 were 7.47, 1.41, 3.99, respectively, based on the hydrogen spectrum of compound **13b**. From the long-range hydrocarbon correlation (HMBC), 139.34 related to 1.41 (H-17 and H-18). The hydrogen associated with H-17 and H-18 only was related to C-9 on the imidazole ring; therefore, 139.34 was related to C-9. 7.47 (H-11) related to 139.34 and 133.28. The aryl carbon that related to H-11 was C-8 or C-9, so 133.28 was a chemical shift of C-8. It was further confirmed that 139.34 was a chemical shift of C-9, and 139.34 (C-9) related to 3.99 (H-19), and 3.99 related to 135.72, 139.34 (C-9). H-19 could relate to C-9 and C-11 on the imidazole ring, so the ethyl group was substituted at the 1-position of the 1,3-imidazole group (Table 2 and Table 3) (Supplementary Materials).

It was verified that Strategy 2 was feasible, i.e., intermediates **11a** or **11b** could undergo mono-substitution reactions with substituent groups. The 1-position nitrogen atom of imidazole was substituted by SN_2_ reaction. The brown solid pure **13a**–**13k** was obtained by washing with methanol.

#### 2.2.3. Synthesis of Phenylahistin Derivatives **15a**–**15q** and **16a**–**16d**

**Scheme 2:**
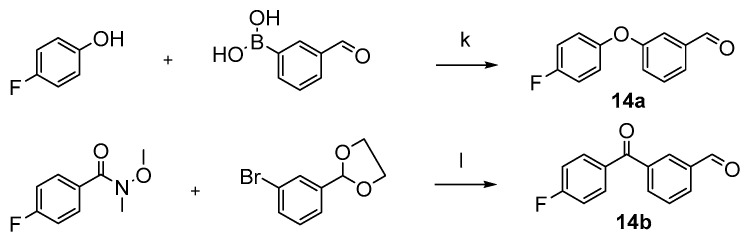
Synthesis of intermediate aldehydes **14a**–**14b**. Regents and conditions: (k) (1) *n*-BuLi, THF, −78 °C, 5 h; (2) HCl (1 M), THF, rt–40 °C, 24 h; two steps yield 67%; (l) Cu(OAc)_2_, Et_3_N, DCM, rt, 48 h, 22%.

To obtain the targeted compounds **15a**–**15q** and **16a**–**16d** derivatives, **14a** and **14b** were prepared, as indicated in Scheme 2. Thus, 4-fluorophenol and phenylboronic acid were coupled via Chan-Lam reaction [19] to obtain p-fluorophenoxybenzaldehyde **14a** and N-methyl-N-methoxy-p-fluorobenzamide was reacted with 2-(3-(bromophenyl)-1,3- dioxolane to afford p-fluorobenzoylbenzaldehyde **14b** [17].

**Scheme 3:**
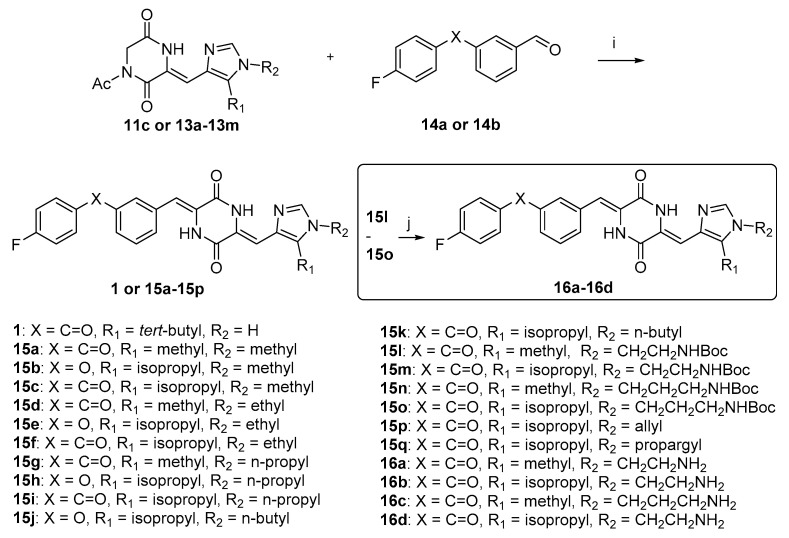
Synthesis of phenylahistin derivatives **15a**–**15q**, **16a**–**16d**. Regents and conditions: (i) Cs_2_CO_3_, Na_2_SO_4_, DMF, 45–55 °C, 24 h, 8–75%; (j) HCl, MeOH, rt, 30–40 °C, 45–85%.

Then compounds **13a**–**13m** were reacted with **14a** or **14b** in DMF under alkaline conditions (Cs_2_CO_3_ or K_2_CO_3_) at 45–55 °C (see Scheme 3). The purification of compounds **15a**–**15q** was performed using methanol-washing or column chromatography to obtain yellow solids. The Boc-protecting groups of **15l**, **15m**, **15n**, and **15o** were removed by hydrochloric acid hydrolysis to obtain **16a**–**16d** as yellow solids (see Scheme 3).

### 2.3. Cytotoxic Activity

#### 2.3.1. Biological Activities of The Synthesized Phenylahistin Derivatives **15a**–**15q** and **16a**–**16d**

**Figure 7:**
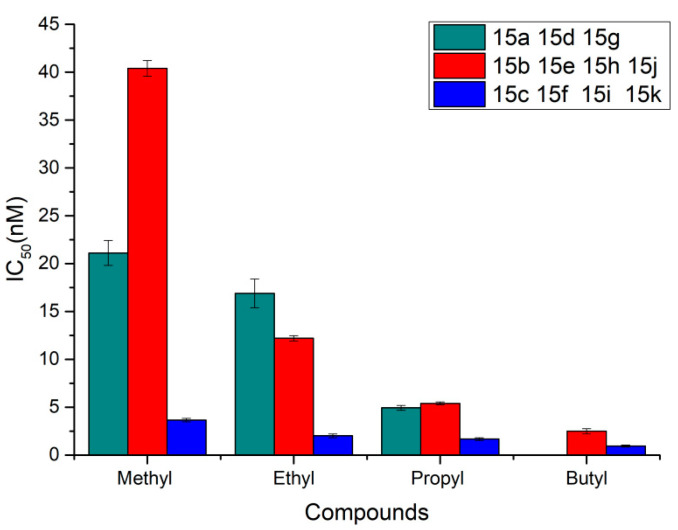
Cytotoxic activities of compounds **15a**-**15k** histogram. (i) olive lines: X = C=O, R_1_ = Methyl, R2 = Methyl, Ethyl, Propyl, respectively; (ii) red lines: X = O, R_1_ = Isopropyl R2 = Methyl, Ethyl, Propyl, Butyl, respectively; (iii) blue lines: X = C=O, R_1_ = Isopropyl, R2 = Methyl, Ethyl, Propyl, Butyl, respectively.

To obtain compounds with greater cytotoxic activity, the 1- (R_1_) and 5- (R_2_) positions of 1,3 imidazole were substituted by alkyl groups, namely compounds **15a**–**15k**. Their bioactivity against human lung cancer NCI-H460 cell line was tested by MTT assay. When X was a carbonyl group and R_1_ was a methyl group, the compound **15a** (IC_50_ = 21.11 nM, R_2_ = methyl), **15d** (IC_50_ = 16.9 nM, R_2_ = ethyl) and **15g** (IC_50_ = 4.93 nM, R_2_ = n-propyl) (Figure 7, olive line) exhibited potent cytotoxic activities against human lung cancer NCI-H460 cell line. With the extension of the carbon chain of the R_2_ substituent, the compounds’ acitvities continued to increase. When X was an oxygen atom or carbonyl group and R_1_ was an isopropyl group, the R_2_ group was substituted with methyl, ethyl, propyl, and butyl groups to obtain compounds **15b**, **15e**, **15h**, **15j** (X = oxygen) (Figure 7 red lines) and **15c**, **15f**, **15i**, **15k** (X = carbonyl group) (Figure 7 blue lines). Compounds **15j** (IC_50_ = 2.49 nM) and **15k** (IC_50_ = 0.94 nM) with butyl groups showed optimal activities. Importantly, the activity of compound **15k** was achieved at the picomolar level. These results suggested that the hydrocarbyl substituents could perform the same function as the tert-butyl-imidazole group, and that the R_2_ hydrocarbon substituent group could enhance the anti-tumor activity of such compounds.

Encouraged by the above research, we designed and synthesized alkylamino- or alkylcarbamate-substituted derivatives, namely compounds **15l**–**15o** and **16a**–**16d**. When the R_2_ was an N-tert-butoxycarbonylaminoethyl group and the R_1_ group was methyl or isopropyl group, compounds **15l** (IC_50_ = 104.78 nM) and **15m** (IC_50_ = 27.26 nM) were obtained. The activities of both compounds significantly decreased the potency compared to compounds **15g** and **15h**, which suggested that substituent groups with a large volume or large stereo space are detrimental to maintaining biological activity. Compounds **16a** and **16b** were prepared by removal of the tert-butoxycarbonyl-protecting group of compounds **15l** and **15m**. Compared with compounds **15l** and **15m**, compounds **16a** and **16b** showed approximately three times greater activity at 33.4 nM and 11.33 nM, respectively. The IC_50_ values of compounds **15n** and **15o** with the N-tert-butoxycarbonylaminopropyl group (R_2_) and methyl or isopropyl group (R_1_) were 145.72 and 12.70 nM, respectively. Cytotoxic activity was greatly improved when the tert-butoxycarbonyl-protecting group weas removed to prepare compounds **16c** and **16d**. The IC_50_ value of compound **16d** was equivalent to that of compound **15h**.

To further explore the structure–activity relationship, unsaturated substituent groups were synthesized to occupy the 1-position of imidazole to obtain compounds **15p** and **15q**. Compounds **15p** (1.03 nM) and **15q** (1.49 nM) with allyl and alkynyl groups, respectively, exhibited potent cytotoxic activities against human lung cancer NCI-H460 cell line.

In summary, compounds **15j** and **15p** exhibited the best antiproliferative activities among the various novel scaffold derivatives. In addition, as reported in Table 4, the cytotoxic activities of compounds **15j**, **16d**, **16b, 15p**, and **15q** were potent at the nanomolar level.

#### 2.3.2. Biological Activities of Phenylahistin and Its Derivatives in Various Cancer Cell Lines 

The cytotoxic activities of compounds **15j**, **15p**, **15q**, **16b**, and **16d** were further evaluated against various other cancer cells, such as pancreatic cancer BxPC-3 cell line and colon cancer HT-29 cell line by MTT assay. The results showed that compounds **15j**, **15p**, **15q**, **16b**, and **16d** exhibited highly potent cytotoxic activities at the nanomolar level against different cancer cell lines as Table 4. For example, the IC_50_ values were 1.03 nM(H460), 0.81 nM(BxPC-3), 0.67 nM(HT-29) for compound **15p**, and 1.49 nM(H460), 1.15 nM(BxPC-3), 0.67 nM(HT-29) for compound **15q**, respectively.

### 2.4. Immunofluorescece Assay

**Figure 8:**
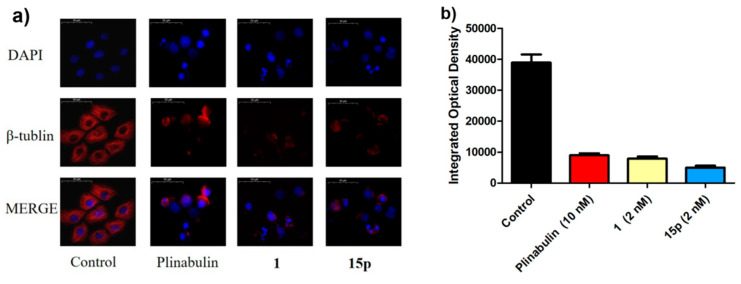
(**a**) Immunofluorescence assays of plinabulin, compounds **1** and **15p**. (**a**) NCI-H460 cells were treated with Plinabulin (10 nM), compounds **1** (2 nM) or **15p** (2 nM) for 24 h and then stained with β-tubulin and DAPI. (i) nuclear (blue); (ii) tubulin (red); (iii) (i) and (ii) were overlapped. (**b**) The inhibition of tubulin polymerization plinabulin, compounds **1** and **15p** through semi-quantitative analysis.

To further explore the effect of phenylahistin derivatives **1** and **15p** on microtubules in cancer cells, an immunofluorescence assay was performed. Figure 8 showed that NCI-H460 cells were treated with plinabulin (10 nM), compounds **1** (2 nM), or **15p** (2 nM) for 24 h and then stained with β-tubulin and DAPI. In comparison with the control group, the microtubule networks were damaged in the other groups (Figure 8a). Semi-quantitative calculations were performed using the software Image Pro Plus 6.0 and shown in Figure 8b. The inhibitory activities were consistent with the previously shown anti-proliferative activities.

### 2.5. Western Blotting Test

**Figure 9:**
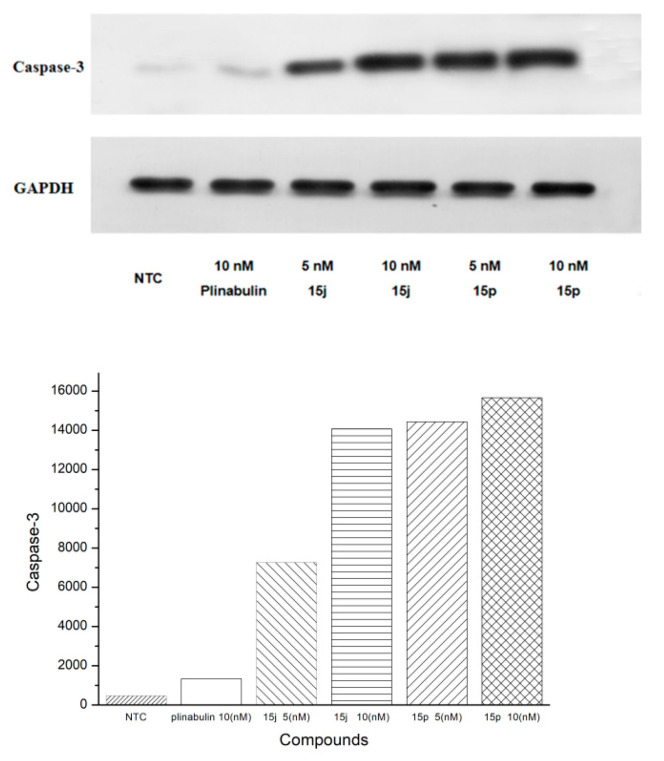
Western blotting analysis reveals caspase-3 expression.

Western blotting (WB) results showed that the expression of caspase-3 in the compounds **15j-** and **15p**-treated groups was significantly higher than that in the control group and plinabulin group. Compound **15p** showed the strongest upregulation of caspase-3 at a concentration of 10 nM (Figure 9).

### 2.6. Theoretical Calculations and Molecular Docking

Theoretical calculations of the physical properties of the synthesized compounds were performed using the Qikprop Module of Maestro software. The interaction modes of compounds **15c, 15f, 15i, 15k, 15p** and **16c** were investigated by molecular docking using Maestro software. In addition, the oil–water partition coefficient (LogPo/w), cell permeability (PCaco) and docking score were calculated and are listed in Table 5. The LogPo/w values of the phenylahistin derivatives ranged from 2.1 to 5.5, which were within a reasonable range of −2.0–6.5. The cell permeability of some compounds was greater than 500, which was beneficial for the improvement in activity.

**Figure 10:**
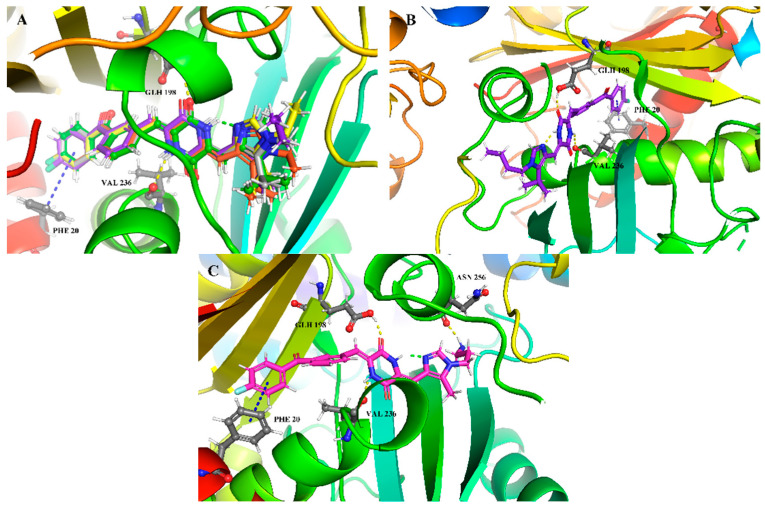
Binding poses of compounds **15c, 15f, 15i, 15k, 16c** (stick-model) with co-crystal structure (PDB: 5XHC). (**A**) Compounds **15c** (light gray), **15f** (orange), **15i** (yellow) and **15k** (violet) are shown as sticks. (**B**) Compounds **15k** (violet) are shown as sticks. (**C**) Compounds **16c** (faded red) are shown as sticks (yellow dashed: hydrogen bonds of ligand-receptor; green dashed: hydrogen bonds of intra-ligand; blue dashed: π–π interaction).

The co-crystal structure of compound **1** shown in Figure 1. Superimposed images of compounds **15c** (light gray), **15f** (orange), **15i** (yellow), and **15k** (purple) are shown in Figure 10A. They were similar to compound **1** in terms of their three-dimensional conformation, and had the same interaction force with tubulin. However, the conformation of the substituent group at position 1 of the 2,3-imidazol-4-yl group was different. The methyl group of **15c** was coplanar with the planar structure formed by 2,5-piperazinedione-imidazole. The ethyl group of **15f** pointed out of the plane, the propyl group of **15i** pointed to the plane, and the butyl group of **15k** pointed out of the plane. The conformations of different lengths of hydrocarbon groups that occupied area 1 might affect the αT5 loop and βT7 loop, which might resulte in disruptions to the dynamic balance of microtubules (the dynamic balance of microtubules is important for them to perform their cell division function) and causing cancer cell death. Figure 10B shows the binding diagram of **15k** and tubulin, which more clearly revealed the effect of butyl on the βT7 loop, in addition to the 1**5k** form of hydrogen bond interactions with GLH198 and VAL236 of tubulin. Compound **16c** also could form π–π interactions with PHE20 as well as other compounds. More importantly, the amino hydrogen atom formed hydrogen bond interactions with the carbonyl oxygen atom of ASN256. This might be an important factor regarding the compound’s activity, as shown in Figure 10C.

**Figure 11:**
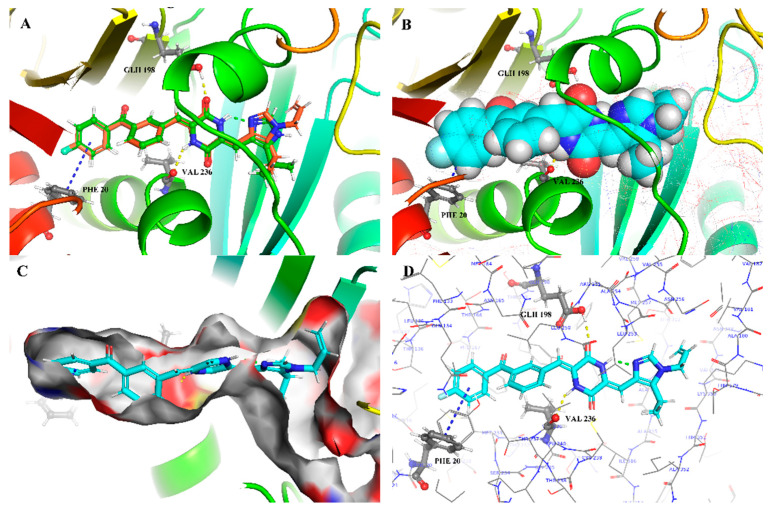
Binding poses of compound **15p** (stick-model) with co-crystal structure (PDB: 5XHC). (**A**) **1** (yellow) and **15p** (orange) are shown as sticks. (**B**) Compound **15p** (teal) are shown as spheres. (**C**) Compound **15p** (teal) are shown as sticks in pocket of tubulin. (**D**) Compound **15p** (teal) are shown as sticks with amino acid residue (yellow dashed: hydrogen bonds of ligand-receptor; green dashed: hydrogen bonds of intra-ligand; blue dashed: π–π interaction).

Figure 11A–D represent diagrams of the interaction between compound **15p** and tubulin. Figure 11A shows the interaction between the molecular stick model and key amino acid residues, including hydrogen bonds, π–π interactions, and intramolecular hydrogen bonds. Figure 11B shows the sphere diagram and surrounding pocket (red mesh) space. Figure 11C shows a schematic diagram of the binding of compound **15p** to the colchicine pocket, and Figure 11D shows the amino acid residues surrounding the molecule. **15p** and **1** could overlap well, and the allyl group pointed in the plane. In fact, the conformations of **15p** are displayed by docking in Figure 11, showing only one of the superimposed molecules and tubulin in the actual state. The surrounding loop conformation was more affected by the allyl substituent group with Z/E conformation, thereby causing stronger damage to the microtubules.

### 2.7. In Vitro Pharmacokinetic Evaluation of Compound **15p**

**Figure 12:**
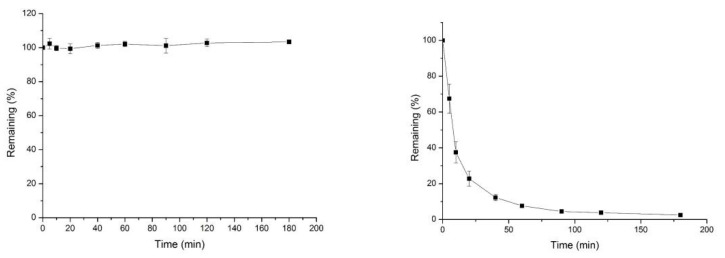
Percentage (%) of **15p** remaining in the plasma or liver microsomes of 37 °C wistar rats at different timepoints. (i) Stability of compound **15p** in plasma. (ii) Stability of compound **15p** in liver microsomes.

In order to explore the metabolic stability of compound **15p** in plasma and liver microsomes, its pharmacokinetic stability was tested in vitro. The plasma stability test results (Figure 12) showed that **15p** was very stable in mouse plasma. This indicated that the amide bond of the core structure 2,5-piperazinedione was be hydrolyzed by esterase. During the liver microsomal stabilization experiment, compound **15p** was gradually metabolized by liver microsomal enzymes over time (Figure 12). Compound **15p** was quickly cleared by liver microsomes (Table 6).

### 2.8. In Vivo Pharmacodynamic Evaluation of Compound **15p**

**Figure 13:**
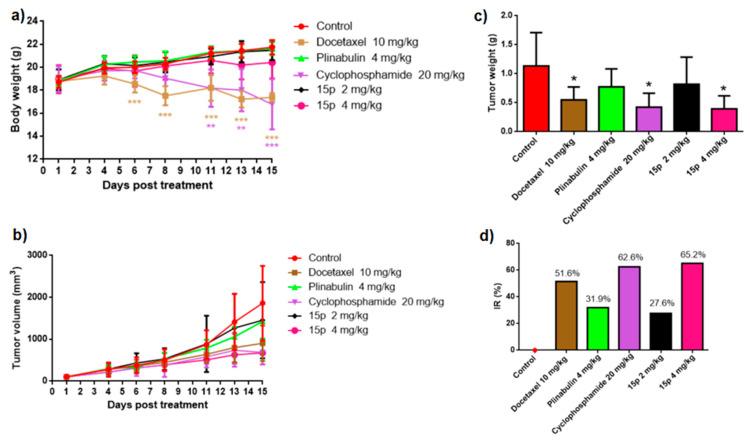
Effect of compound **15p** on body weight (**a**), tumor volume (**b**), tumor weight (**c**) and inhibition rate (**d**) in mice bearing H22 hepatoma carcinoma cells. Data were presented as mean ± SD. * *p* < 0.05, ** *p* < 0.01, *** *p* < 0.01, compared with the control group. Tumor weight inhibition rate: IR (%) = (1 − TWt/TWc) × 100%, TWt: tumor weight of the treatment group; TWc: tumor weight of the model control group.

The anticancer effect of the compound **15p** was further evaluated against H22 tumor-bearing mice models by intravenous injection at doses of 2 mg/kg, or 4 mg/kg, two or three times a week, and for 14 consecutive days. Docetaxel (10 mg/kg), plinabulin (4 mg/kg), and cyclophosphamide (20 mg/kg) were used as the positive controls. At the end of administration period, the average value of the final tumor volumes of vehicle, docetaxel (10 mg/kg), plinabulin (4 mg/kg), cyclophosphamide (20 mg/kg) and compound **15p** (2 mg/k; 4 mg/kg) were 1861.4, 903.6, 1425.3, 687.2, 1453.6, and 674.5 mm^3^ (Table 7), respectively. The average excised tumor weights of the corresponding groups were 1.13 g, 0.55 g, 0.77 g, 0.42 g, 0.82 g, and 0.39 g, respectively (Figure 13c), and the inhibitory rates (IR) were 51.6%, 31.9%, 62.6%, 27.6%, and 65.2%, respectively (Figure 13d and Table 7). Compound **15p** with 4 mg/kg had a strong inhibitory action regarding the growth of the H22-transplanted tumor. Overall, these results suggest that compound **15p** displayed dose-dependent effects in the concentration range of 2–4 mg/kg. Compared with docetaxel at 10 mg/kg, both the tumor weight and tumor volume were reduced in the 4 mg/kg group of **15p**, while the body weight was not significantly different (Figure 13a). However, both docetaxel and cyclophosphamide decreased the body weights compared to the control group.

## 3. Materials and Methods

### 3.1. General

All starting materials were purchased from commercial suppliers and used without further purification. Column chromatography was performed on silica gel (200−300 mesh, Yantai Chemical Industry Research Institute). Thin-layer chromatography (TLC) was performed using silica gel GF-254 plates (Xinzheng Experimental Equipment Co., Ltd., Linyi, China) with detection by UV (254 nm or 365 nm). Melting points were measured on a Yidianwuguang WRS-3 melting point instrument (China). ^1^H and ^13^C NMR spectra were obtained on a JEOL 400 spectrometer (400 MHz) or Agilent Pro pulse 500 MHz spectrometer with TMS as an internal standard. The following abbreviations were used to explain the multiplicities: s = singlet, d = doublet, t = triplet, q = quartet, b = broad, td = triple doublet, dt = double triplet, dq = double quartet, m = multiplet. High-resolution mass spectra (ESI or EI) were recorded on Agilent 1290 Infinity II UHPLC/6530 Q-TOF mass spectrometer.

### 3.2. Synthesis

#### 3.2.1. Preparation of Ethyl 5-(isopropyl) Oxazole-4-carboxylate (**6**)

1, 8-diazabicyclo[5.4.0]undec-7-ene (DBU) (80.7 g, 0.53 mol) and pivalic anhydride (83.8 g, 0.53 mol) were added dropwise to a solution of ethyl isocyanoacetate (50 g, 441.66 mmol) in THF (200 mL). The mixture was stirred for 48 h at room temperature. The reaction solution was removed by evaporation under reduced pressure. The residue was extracted with EtOAc, then washed with 10% Na_2_CO_3_ and 10% citric acid. The combined organic layer was washed with saturated brine, and dried over anhydrous Mg_2_SO_4_. The solvent was concentrated in vacuo, and the crude product was purified by column chromatography (EtOAc–petroleum ether, 2:1) to produce **6** (73 g) as a yellow oil [20]. Yield: 95%. ^1^H NMR (500 MHz, CDCl_3_) *δ* 7.73 (s, 1H), 4.37 (q, *J* = 7.1 Hz, 2H), 3.79 (dt, *J* = 14.0, 7.0 Hz, 1H), 1.38 (t, *J* = 7.1 Hz, 3H), 1.28 (d, *J* = 7.0 Hz, 6H). MS (ESI) *m/z*: [M + H]^+^ Calcd for C_9_H_14_NO_3_: 184.10, Found: 183.74.

#### 3.2.2. Preparation of Ethyl 5-(isopropyl)-1H-imidazole-4-carboxylate (**7**)

A mixture of compound **6** (5 g, 27.29 mmol) and formamide (49.2 g (43 mL), 1091.70 mmol) was heated at 180 °C for 48 h. After cooling, the reaction mixture was added with 10% Na_2_CO_3_ (60 mL), and extracted with petroleum ether, then extracted with EtOAc. The petroleum ether layer was given up, and the EtOAc layers were combined and washed with saturated brine and dried over anhydrous Mg_2_SO_4_. The solvent was evaporated in vacuo, and the crude product was purified by slurry using H_2_O to obtain 3.0 g compound **7** [19]. Yield: 60%. Faded orange solid. ^1^H NMR (500 MHz, CDCl_3_) δ 7.60 (s, 1H), 4.31 (q, *J* = 7.1 Hz, 2H), 3.77 (s, 1H), 1.30 (t, *J* = 7.7 Hz, 9H). MS (ESI) *m/z*: [M + H]^+^ Calcd for C_9_H_15_N_2_O_2_: 183.11, Found: 182.77.

#### 3.2.3. Preparation of (5-(isopropyl)-1H-imidazol-4-yl) Methanol (**8**)

Ethyl 5-(*tert*-butyl)-1*H*-imidazole-4-carboxylate (2.5 g, 13.70 mmol) in THF (10 mL) was added dropwise at 0 °C under nitrogen to a solution of LiAlH_4_ (1.6 g, 41.1 mmol) in THF (10 mL). The mixture was stirred for 4.5 h at room temperature. Water was added to this solution, and the resulting precipitate was removed by celite filtration. The filtrate was evaporated in vacuo to obtain a white solid (1.9 g) with a yield at 99%. The crude product was used in the next step without further purification [19].

Yield: 99%. White solid. MS (ESI) *m/z*: [M + H]^+^ Calcd for C_7_H_13_N_2_O: 141.10, Found: 140.68.

#### 3.2.4. Preparation of 5-(isopropyl)-1H-imidazole-4-carbaldehyde (**9b**)

MnO_2_ (11.9 g, 0.14 mmol) was added to a solution of compound **8** (1.9 g, 13.70 mmol) in DCM (20 mL), and the mixture was stirred for 48 h at 25 °C. The reaction was monitored by TLC. The solution was filtrated using celite filtration. The solvent was evaporated in vacuo, and the crude product was purified by column chromatography (EtOAc–petroleum ether, 4:1) to obtain a white solid 1.2 g with a yield at 61% [20]. 

Yield: 61%. White solid. ^1^H NMR (500 MHz, CDCl_3_) δ 9.88 (s, 1H), 7.79 (s, 1H), 3.56 – 3.43 (m, 1H), 1.37 (d, *J* = 6.9 Hz, 6H). MS (ESI) *m/z*: [M + H]^+^ Calcd for C_7_H_11_N_2_O: 139.09, Found: 138.65.

#### 3.2.5. Preparation of (Z)-1-acetyl-3-((1H-imidazol-4-yl) methylene) Piperazine-2, 5-dione (**11a**)

General procedure for the synthesis of **11a** and **11b**, and synthesis of **11a** as an example.

Under nitrogen, a mixture of compound **9a** (1.0 g, 9.08 mmol), 1, 4-diacetylpiperazine-2, 5-dione (13.6 g, 18.16 mmol) and Cs_2_CO_3_ (4.4 g, 13.62 mmol) in DMF (18 mL) was stirred for 20 h at room temperature. The reaction solution was poured into cool water to precipitate a solid. The mixture solution was filtered to provide an orange solid 1.1 g with a yield of 47%.

Yield: 47%. Orange solid. ^1^H NMR (500 MHz, DMSO-*d_6_*) *δ* 12.56 (s, 1H), 11.74 (s, 1H), 7.87 (s, 1H), 6.76 (s, 1H), 4.31 (s, 2H), 2.50 (s, 3H), 2.33 (s, 3H). MS (ESI) *m/z*: [M + H]^+^ Calcd for C_11_H_13_N_4_O_3_: 249.10, Found: 248.81.

#### 3.2.6. Preparation of (Z)-1-acetyl-3-((5-(isopropyl)-1H-imidazol-4-yl) methylene) Piperazine-2, 5-dione (**11b**)

Yield: 36%. Orange solid. ^1^H NMR (500 MHz, DMSO-*d_6_*) δ 12.60 (s, 1H), 11.78 (s, 1H), 7.90 (s, 1H), 6.80 (s, 1H), 4.31 (s, 2H), 3.30–3.16 (m, 1H), 2.50 (d, *J* = 3.9 Hz, 3H), 1.24 (d, *J* = 6.9 Hz, 6H). MS (ESI) *m/z*: [M + H]^+^ Calcd for C_13_H_17_N_4_O_3_: 277.13, Found: 276.83.

#### 3.2.7. Preparation of (Z)-1-acetyl-3-((5-methyl-1-methyl-imidazol-4-yl) Methylene) Piperazine-2, 5-dione (**13a**)

General procedure for synthesis of **13a**–**13m**, and synthesis of **13a** is used as an example.

A mixture of (Z)-1-acetyl-3-((5-methyl-1*H*-imidazol-4-yl) methylene) piperazine-2, 5-dione (100 mg, 0.40 mmol), NaH(48.3 mg (60%), 1.21 mmol) was stirred in DMF (2 mL) under nitrogen at −30 °C for 20 min. Then, iodomethane (571.8 mg, 4.03 mmol) was added dropwise. The reaction was continued with stirring for 1 h. The reaction was detected by LC-MS. After the reaction was completed, the aqueous ammonium chloride solution was added to quench the reaction. The mixture solution was filtered. Then, the filter cake was washed with water and dried in vacuum drying equipment at 50 °C to obtain a faded orange solid 47 mg (13a) with a yield of 45%. The product was directly used in the next reaction without further purification.

^1^H NMR (500 MHz, CDCl_3_) *δ* 11.84 (s, 1H), 7.48 (s, 1H), 6.88 (s, 1H), 4.47 (s, 2H), 3.61 (s, 3H), 2.65 (s, 3H), 2.34 (s, 3H). MS (ESI) *m/z*: [M + H]^+^ Calcd for C_12_H_15_N_4_O_3_: 263.1139, Found: 262.83. 

#### 3.2.8. Preparation of (Z)-1-acetyl-3-((5-isopropyl-1-methyl-imidazol-4-yl) methylene) Piperazine-2, 5-dione (**13b**)

Yield: 57%. Orange solid 60 mg. ^1^H NMR (500 MHz, CDCl_3_) *δ* 11.98 (s, 1H), 7.39 (s, 1H), 7.02 (s, 1H), 4.46 (s, 2H), 3.67 (s, 3H), 3.31–3.16 (m, 1H), 2.64 (s, 3H), 1.39 (d, *J* = 7.2 Hz, 6H). MS (ESI) *m/z*: [M + H]^+^ Calcd for C_14_H_19_N_4_O_3_: 291.1452, Found: 290.75.

#### 3.2.9. Preparation of (Z)-1-acetyl-3-((5-methyl-1-ethyl-imidazol-4-yl) methylene) Piperazine-2, 5-dione (**13c**)

Yield: 56%. Orange solid 127 mg. ^1^H NMR (500 MHz, CDCl_3_) *δ* 11.86 (s, 1H), 7.51 (s, 1H), 6.88 (s, 1H), 4.46 (s, 2H), 3.94 (q, *J* = 7.3 Hz, 2H), 2.64 (s, 3H), 2.33 (s, 3H), 1.44 (t, *J* = 7.3 Hz, 3H). MS (ESI) *m/z*: [M + H]^+^ Calcd for C_13_H_17_N_4_O_3_: 277.1295, Found: 276.74.

#### 3.2.10. Preparation of (Z)-1-acetyl-3-((5-isopropyl-1-ethyl-imidazol-4-yl) methylene) Piperazine-2, 5-dione (**13d**)

Yield: 68%. Orange solid 112 mg. ^1^H NMR (500 MHz, CDCl_3_) *δ* 12.02 (s, 1H), 7.45 (s, 1H), 7.05 (s, 1H), 4.46 (s, 2H), 3.99 (q, *J* = 7.3 Hz, 2H), 3.16 (dt, *J* = 14.4, 7.2 Hz, 1H), 2.64 (s, 3H), 1.45 (t, *J* = 7.3 Hz, 3H), 1.41 (d, *J* = 7.2 Hz, 6H). MS (ESI) *m/z*: [M + H]^+^ Calcd for C_15_H_21_N_4_O_3_: 305.1608, Found: 304.82.

#### 3.2.11. Preparation of (Z)-1-acetyl-3-((5-methyl-1-(n-propyl)-imidazol-4-yl) Methylene) piperazine-2, 5-dione (**13e**)

Yield: 73%. Orange solid 129 mg. ^1^H NMR (500 MHz, CDCl_3_) *δ* 11.86 (s, 1H), 7.48 (s, 1H), 6.89 (s, 1H), 4.46 (s, 2H), 3.85 (t, *J* = 7.2 Hz, 2H), 2.64 (s, 3H), 2.32 (s, 3H), 1.79 (dd, *J* = 14.6, 7.3 Hz, 2H), 0.96 (t, *J* = 7.4 Hz, 3H). MS (ESI) *m/z*: [M + H]^+^ Calcd for C_14_H_19_N_4_O_3_: 291.1452, Found: 291.26.

#### 3.2.12. Preparation of (Z)-1-acetyl-3-((5-isopropyl-1-(n-propyl)-imidazol-4-yl) Methylene) piperazine-2, 5-dione (**13f**)

Yield: 43%. Orange solid 74 mg. ^1^H NMR (500 MHz, CDCl_3_) *δ* 12.02 (s, 1H), 7.42 (s, 1H), 7.06 (s, 1H), 4.46 (s, 2H), 4.01–3.80 (m, 2H), 3.24–2.99 (m, 1H), 2.64 (s, 3H), 1.79 (dd, *J* = 14.8, 7.4 Hz, 2H), 1.41 (d, *J* = 7.2 Hz, 6H), 0.98 (t, *J* = 7.4 Hz, 3H). MS (ESI) *m/z*: [M + H]^+^ Calcd for C_16_H_23_N_4_O_3_: 319.1765, Found: 319.23.

#### 3.2.13. Preparation of (Z)-1-acetyl-3-((5-isopropyl-1-(n-butyl)-imidazol-4-yl) Methylene) piperazine-2, 5-dione (**13g**)

Yield: 41%. Orange solid 148 mg. ^1^H NMR (400 MHz, CDCl_3_) *δ* 12.03 (s, 1H), 7.42 (s, 1H), 7.05 (s, 1H), 4.45 (s, 2H), 3.90 (t, *J* = 7.4 Hz, 2H), 3.13 (dt, *J* = 14.3, 7.1 Hz, 1H), 2.64 (s, 3H), 1.80–1.67 (m, 2H), 1.46–1.25 (m, 8H), 0.97 (t, *J* = 7.4 Hz, 3H). MS (ESI) *m/z*: [M + H]^+^ Calcd for C_17_H_25_N_4_O_3_: 333.1921, Found: 332.86.

#### 3.2.14. Preparation of (Z)-1-acetyl-3-((5-methyl-1-(N-boc-amino- ethyl)-imidazol-4-yl) methylene) Piperazine-2, 5-dione (**13h**)

Yield: 80%. Orange solid 378 mg. ^1^H NMR (500 MHz, DMSO-*d_6_*) *δ* 11.54 (s, 1H), 8.13 (s, 1H), 7.77 (s, 1H), 6.43 (s, 1H), 4.03 (d, *J* = 1.4 Hz, 2H), 3.99 (t, *J* = 5.9 Hz, 2H), 3.21 (dd, *J* = 11.9, 5.9 Hz, 2H), 2.26 (s, 3H), 1.34 (s, 9H). MS (ESI) *m/z*: [M + H]^+^ Calcd for C_18_H_26_N_5_O_5_: 392.1928, Found: 392.40.

#### 3.2.15. Preparation of (Z)-1-acetyl-3-((5-methyl-1-(N-boc-amino- propyl)-imidazol-4-yl) methylene) Piperazine-2, 5-dione (**13j**)

Yield: 75%. Orange solid 367 mg. ^1^H NMR (500 MHz, CDCl_3_) *δ* 11.84 (s, 1H), 7.57 (s, 1H), 6.88 (s, 1H), 4.47 (s, 2H), 3.95 (t, *J* = 7.3 Hz, 2H), 3.19 (d, *J* = 6.3 Hz, 2H), 2.64 (s, 3H), 2.33 (s, 3H), 2.01–1.90 (m, 2H), 1.46 (s, 9H). MS (ESI) *m/z*: [M + H]^+^ Calcd for C_19_H_28_N_5_O_5_: 406.2085, Found: 406.00.

#### 3.2.16. Preparation of (Z)-1-acetyl-3-((5-isopropyl-1-(N-boc-amino- propyl)-imidazol-4-yl) methylene) Piperazine-2, 5-dione (**13k**)

Yield: 60%. Orange solid 260 mg. ^1^H NMR (500 MHz, CDCl_3_) δ 12.00 (s, 1H), 7.50 (s, 1H), 7.04 (s, 1H), 4.45 (s, 2H), 4.02–3.93 (m, 2H), 3.20 (d, *J* = 6.2 Hz, 2H), 3.11 (dd, *J* = 14.3, 7.2 Hz, 1H), 2.64 (s, 3H), 1.98–1.90 (m, 2H), 1.45 (s, 9H), 1.40 (d, *J* = 7.2 Hz, 6H).

#### 3.2.17. Preparation of (Z)-1-acetyl-3-((5-isopropyl-1-allyl-imidazol-4-yl) methylene) Piperazine-2, 5-dione (**13l**)

Yield: 59%. Orange solid 136 mg. ^1^H NMR (500 MHz, CDCl_3_) *δ* 11.99 (s, 1H), 7.43 (s, 1H), 7.05 (s, 1H), 5.94 (ddd, *J* = 22.0, 10.2, 5.0 Hz, 1H), 5.30 (d, *J* = 10.4 Hz, 1H), 5.02 (d, *J* = 17.1 Hz, 1H), 4.56 (d, *J* = 4.9 Hz, 2H), 4.46 (s, 2H), 3.12 (dq, *J* = 14.3, 7.1 Hz, 1H), 2.64 (s, 3H), 1.38 (d, *J* = 7.2 Hz, 6H). MS (ESI) *m/z*: [M + H]^+^ Calcd for C_16_H_21_N_4_O_3_: 317.1608, Found: 316.76.

#### 3.2.18. Preparation of (Z)-1-acetyl-3-((5-isopropyl-1-(propargyl)-imidazol-4-yl) Methylene) piperazine-2, 5-dione (**13m**)

Yield: 68%. Orange solid 232 mg. ^1^H NMR (500 MHz, CDCl_3_) *δ* 11.91 (s, 1H), 7.58 (s, 1H), 7.03 (s, 1H), 4.71 (d, *J* = 2.5 Hz, 2H), 4.46 (s, 2H), 3.26 (dt, *J* = 14.3, 7.2 Hz, 1H), 2.64 (s, 3H), 2.52 (t, *J* = 2.5 Hz, 1H), 1.43 (d, *J* = 7.2 Hz, 6H). MS (ESI) *m/z*: [M + H]^+^ Calcd for C_16_H_19_N_4_O_3_: 315.1452, Found: 314.79.

#### 3.2.19. Preparation of 3-(4-fluorophenoxy)benzaldehyde (**14a**)

The following literature was referenced for this part of the experiment [17].

To a solution of 4-fluorophenol (500 mg, 4.46 mol) in dry DCM (10 mL), (3-formylphenyl)boronic acid(1.00 g, 6.69 mol), Cu(OAc)_2_ (811 mg, 4.46 mol) and Et_3_N (135.6 mg, 1.34 mol) were added under O_2_ atmosphere, then the mixture was stirred at 25 °C for 48 h. the mixture was diluted with brine and extracted with EtOAc. The solvent was removed under reduced pressure. The residue was purified by silica gel column chromatography using petroleum ether/ethyl acetate (10:1) to give white solid 0.22 g with a yield of 22%.

^1^H NMR (500 MHz, CDCl_3_) δ 9.96 (s, 1H), 7.59 (d, *J* = 7.5 Hz, 1H), 7.50 (t, *J* = 7.8 Hz, 1H), 7.41 (dd, *J* = 2.2, 1.4 Hz, 1H), 7.25 (dt, *J* = 3.5, 1.4 Hz, 1H), 7.10–7.04 (m, 2H), 7.02 (ddd, *J* = 6.8, 5.2, 3.0 Hz, 2H). MS (ESI) *m/z*: [M + H]^+^ Calcd for C_13_H_10_FO_2_: 217.07, Found: 217.27.

#### 3.2.20. Preparation of 3-(4-fluorobenzoyl)benzaldehyde (**14b**)

The literature was referenced for this part of the experiment [17].

The tetrahydrofuran solution (12 mL) of 2-(3-Bromophenyl)-1, 3-dioxolane (4.88 g, 21.29 mmol) was added dropwise to a solution of *n*-BuLi (20.48 ml (1.6 M THF)), 32.75 mmol) in dry THF (20 mL) at −78 °C under nitrogen atmosphere. Then, the solution was stirred at −78 °C under nitrogen atmosphere for 50 min. Then, a solution of 4-fluoro-N-methyl-N-methoxybenzamide (3.0 g, 16.38 mmol) in THF (10 ml) was added dropwise, and the mixture was stirred at −78 °C for 2 h. The reaction mixture was quenched using a saturated ammonium chloride solution. The residue was dissolved in methanol and hydrochloric acid (2 mol/L) was added. The mixture was stirred at room temperature. The solvent was moved under pressure, extracted with EtOAc (100 mL × 3), washed with saturated NaCl (50 mL × 3), and dried over anhydrous sodium sulfate. The solvent was concentrated in vacuo. The residue was purified by silica gel column chromatography using petroleum ether/ethyl acetate (10:1/8:1) to provide a white solid 2.51 g with a yield of 67%. 

^1^H NMR (500 MHz, DMSO-*d_6_*) δ 10.11 (s, 1H), 8.24 – 8.18 (m, 2H), 8.07–8.03 (m, 1H), 7.90–7.85 (m, 2H), 7.80 (t, *J* = 7.7 Hz, 1H), 7.46–7.39 (m, 2H). MS (ESI) *m/z*: [M + H]^+^ Calcd for C_14_H_10_FO_2_: 229.07, Found: 228.75.

#### 3.2.21. (3. Z, 6Z)-3-(4-fluorobenzoyl)benzylidene)-6-((5-methyl-1-methyl- imidazol-4-yl)methylene)piperazine-2, 5-dione (**15a**) 

General procedure for synthesis of 15a-15q, and synthesis of 15a are used as an example.

A mixture of (Z)-1-acetyl-3-((5-(methyl)-1-methyl-imidazol-4-yl) methylene) piperazine-2, 5-dione (43 mg, 0.16 mmol), 3- (4-fluorobenzyloxy) benzaldehyde (44.9 mg, 0. 20 mmol), Cs_2_CO_3_ (80.2 mg, 0.25 mmol), Na_2_SO_4_ (46.6 mg, 0.33 mmol) was stirred in DMF (1.5 mL) under nitrogen at 55 °C for 24 h. The reaction solution was poured into cold water (20 mL). The filter cake was re-dissolved using methanol and dichloromethane (1:3) and filtered. The solvent was combined and concentrated under reduced pressure. The filtration was stirred in methanol at room temperature for 2 h, then moved to 0 °C. The solution was filtered, washed with methanol, and dried in vacuum at 50 °C to obtain a yellow solid 51 mg (15a) with a yield of 72%. 

Yield: 72%, yellow solid 51.0 mg, MP: 224–226 °C. ^1^H NMR (500 MHz, DMSO-*d_6_*) *δ* 11.90 (s, 1H), 10.30 (s, 1H), 7.95-7.87 (m, 3H), 7.82 (s, 1H), 7.75 (d, *J* = 7.6 Hz, 1H), 7.63 (d, *J* = 7.7 Hz, 1H), 7.58 (t, *J* = 7.6 Hz, 1H), 7.40 (t, *J* = 8.8 Hz, 2H), 6.80 (s, 1H), 6.58 (s, 1H), 3.61 (s, 3H), 2.29 (s, 3H). ^13^C NMR (125 MHz, DMSO-*d_6_*) *δ* 194.17, 164.74 (*J_C-F_* = 251.6 Hz), 157.49, 156.02, 137.85, 137.27, 133.54, 133.45 (*J_C-F_* = 2.8 Hz), 133.32, 133.11, 132.77×2 (*J_C-F_* = 9.4 Hz), 130.76, 130.11, 128.85, 128.69, 127.70, 123.72, 115.66×2 (*J_C-F_* = 21.9 Hz), 112.64, 104.19, 31.34, 7.92. MS (ESI) *m/z*: [M + H]^+^ Calcd for C_24_H_20_FN_4_O_3_: 431.1514, Found: 431.1514.

#### 3.2.22. (3. Z, 6Z)-3-(4-fluorophenoxy)benzylidene)-6-((5-isopropyl-1-methyl- imidazol-4-yl)methylene) piperazine-2, 5-dione (**15b**) 

Yield: 37%, yellow solid 17 mg, MP: 218–219 °C. ^1^H NMR (500 MHz, DMSO-*d_6_*) *δ* 11.98 (s, 1H), 10.12 (s, 1H), 7.84 (s, 1H), 7.41 (t, *J* = 7.9 Hz, 1H), 7.29–7.20 (m, 3H), 7.17 (s, 1H), 7.12 (dd, *J* = 8.9, 4.5 Hz, 2H), 6.90 (d, *J* = 6.6 Hz, 1H), 6.70 (d, *J* = 13.7 Hz, 2H), 3.68 (s, 3H), 3.31–3.23 (m, 1H), 1.32 (d, *J* = 7.1 Hz, 6H). ^13^C NMR (125 MHz, DMSO-*d_6_*) *δ* 159.1, 157.2, 157.0, 156.8 (*J_C-F_* = 155.7 Hz), 152.6, 138.6, 138.1, 135.2, 132.2, 130.3, 127.3, 124.4, 123.8, 120.5×2 (*J_C-F_* = 8.4 Hz), 118.9, 117.7, 116.6×2 (*J_C-F_* = 23.2 Hz), 113.1, 104.1, 32.2, 23.8, 21.9×2.MS (ESI) *m/z*: [M + H]^+^ Calcd for C_25_H_24_FN_4_O_3_: 447.1827, Found 447.1825.

#### 3.2.23. (3. Z, 6Z)-3-(4-fluorobenzoyl)benzylidene)-6-((5-isopropyl-1-methyl- imidazol-4-yl)methylene) piperazine-2, 5-dione (**15c**)

Yield: 61%, yellow solid 78 mg, MP: 225–227 °C. ^1^H NMR (500 MHz, DMSO-*d_6_*) *δ*12.00 (s, 1H), 10.35 (s, 1H), 7.92 (dd, *J* = 8.7, 5.6 Hz, 2H), 7.84 (d, 2H), 7.76 (d, *J* = 7.6 Hz, 1H), 7.64 (d, *J* = 7.7 Hz, 1H), 7.59 (t, *J* = 7.6 Hz, 1H), 7.40 (t, *J* = 8.8 Hz, 2H), 6.80 (s, 1H), 6.69 (s, 1H), 3.68 (s, 3H), 3.27 (dt, *J* = 14.3, 7.1 Hz, 1H), 1.32 (d, *J* = 7.1 Hz, 6H). ^13^C NMR (125 MHz, DMSO-*d_6_*) *δ* 194.16, 164.74 (*J_C-F_* = 251.6 Hz), 157.57, 156.10, 138.64, 138.14, 137.26, 133.58, 133.44 (*J_C-F_* = 2.8 Hz), 133.33, 132.76*2 (*J_C-F_* = 9.4 Hz), 132.21, 130.10, 128.83, 128.67, 127.77, 123.88, 115.69×2 (*J_C-F_*= 22.0 Hz), 112.70, 104.13, 32.19, 23.84, 21.85×2. MS (ESI) *m/z*: [M + H]^+^ Calcd for C_26_H_24_FN_4_O_3_: 459.1827, Found: 459.1818.

#### 3.2.24. (3. Z, 6Z)-3-(4-fluorobenzoyl)benzylidene)-6-((5-methyl-1-ethyl- imidazol-4-yl)methylene)- piperazine-2, 5-dione (**15d**)

Yield: 24%, yellow solid 47 mg, MP: 227–229 °C. ^1^H NMR (500 MHz, DMSO-*d_6_*) *δ*11.91 (s, 1H), 10.31 (s, 1H), 7.95 (s, 1H), 7.91 (dd, *J* = 8.3, 5.7 Hz, 2H), 7.82 (s, 1H), 7.75 (d, *J* = 7.4 Hz, 1H), 7.63 (d, *J* = 7.7 Hz, 1H), 7.58 (t, *J* = 7.6 Hz, 1H), 7.40 (t, *J* = 8.7 Hz, 2H), 6.80 (s, 1H), 6.58 (s, 1H), 4.00 (q, *J* = 7.2 Hz, 2H), 2.31 (s, 3H), 1.32 (t, *J* = 7.2 Hz, 3H). ^13^C NMR (125 MHz, DMSO-*d_6_*) *δ* 194.17, 164.73 (*J_C-F_* = 251.4 Hz), 157.49, 156.03, 137.27, 136.90, 133.54, 133.46, 133.32, 133.28, 132.77×2 (*J_C-F_* = 9.4 Hz), 130.10, 129.91, 128.85, 128.69, 127.71, 123.77, 115.66×2 (*J_C-F_* = 9.60, 22.0 Hz), 112.64, 104.12, 15.68, 7.90. MS (ESI) *m/z*: [M + H]^+^ Calcd for C_25_H_22_FN_4_O_3_: 445.1670, Found: 445.1668.

#### 3.2.25. (3. Z, 6Z)-3-(4-fluorophenoxy)benzylidene)-6-((5-isopropyl-1-ethyl- imidazol-4-yl)methylene)- piperazine-2, 5-dione (**15e**)

Yield: 30%, yellow solid 70 mg, MP: 195–198 °C. ^1^H NMR (500 MHz, DMSO-*d_6_*) *δ* 12.02 (s, 1H), 10.13 (s, 1H), 7.92 (s, 1H), 7.42 (t, *J* = 7.9 Hz, 1H), 7.31-7.22 (m, 3H), 7.18 (s, 1H), 7.15-7.11 (m, 2H), 6.92 (dd, *J* = 8.1, 2.1 Hz, 1H), 6.72 (d, *J* = 9.5 Hz, 2H), 4.07 (q, *J* = 7.2 Hz, 2H), 3.32-3.22 (m, 1H), 1.34 (d, *J* = 7.0 Hz, 9H). ^13^C NMR (125 MHz, DMSO-*d_6_*) *δ* 159.1, 157.2, 157.0, 156.8 (*J_C-F_* = 154.2 Hz), 152.6, 138.3, 136.7, 135.2, 132.1, 130.3, 127.2, 124.4, 123.7, 120.5×2 (*J_C-F_* = 8.4 Hz), 118.9, 117.7, 116.5×2 (*J_C-F_* = 23.2 Hz), 113.1, 104.2, 39.7, 23.7, 22.5×2, 16.5. MS (ESI) *m/z*: [M + H]^+^ Calcd for C_26_H_26_FN_4_O_3_: 461.1983, Found 461.1973.

#### 3.2.26. (3. Z, 6Z)-3-(4-fluorobenzoyl)benzylidene)-6-((5-isopropyl-1-ethyl- imidazol-4-yl)methylene)- piperazine-2, 5-dione (**15f**)

Yield: 61%, yellow solid 105 mg, MP: 234–236 °C. ^1^H NMR (500 MHz, DMSO-*d_6_*) *δ* 12.01 (s, 1H), 10.34 (s, 1H), 7.94-7.87 (m, 3H), 7.82 (s, 1H), 7.76 (d, *J* = 7.6 Hz, 1H), 7.63 (d, *J* = 7.7 Hz, 1H), 7.58 (t, *J* = 7.6 Hz, 1H), 7.40 (t, *J* = 8.8 Hz, 2H), 6.79 (s, 1H), 6.69 (s, 1H), 4.04 (p, *J* = 7.2 Hz, 2H), 3.22 (dt, *J* = 14.3, 7.1 Hz, 1H), 1.33 (dd, *J* = 7.1, 5.6 Hz, 9H). ^13^C NMR (125 MHz, DMSO-*d_6_*) *δ* 194.17, 164.74 (*J_C-F_* = 251.6 Hz), 157.59, 156.14, 138.24, 137.26, 136.69, 133.61, 133.45 (*J_C-F_* = 2.8 Hz), 133.33, 132.77×2 (*J_C-F_* = 9.4 Hz), 132.12, 130.09, 128.83, 128.67, 127.82, 123.80, 115.70×2 (*J_C-F_* = 21.9 Hz), 112.69, 104.18, 23.67, 22.47×2, 16.51. MS (ESI) *m/z*: [M + H]^+^ Calcd for C_27_H_26_FN_4_O_3_: 473.1983, Found: 473.1986.

#### 3.2.27. (3. Z, 6Z)-3-(4-fluorobenzoyl)benzylidene)-6-((5-methyl-1-(n-propyl)-imidazol-4- yl)methylene)- piperazine-2, 5-dione (**15g**)

Yield: 34%, yellow solid 54 mg, MP: 214–216 °C. ^1^H NMR (500 MHz, DMSO-*d_6_*) *δ* 11.91 (s, 1H), 10.30 (s, 1H), 7.93 (s, 1H), 7.91 (dd, *J* = 8.8, 5.6 Hz, 2H), 7.82 (s, 1H), 7.75 (d, *J* = 7.6 Hz, 1H), 7.63 (d, *J* = 7.7 Hz, 1H), 7.58 (t, *J* = 7.6 Hz, 1H), 7.40 (t, *J* = 8.8 Hz, 2H), 6.80 (s, 1H), 6.58 (s, 1H), 3.93 (t, *J* = 7.2 Hz, 2H), 2.31 (s, 3H), 1.69 (dd, *J* = 14.5, 7.3 Hz, 2H), 0.86 (t, *J* = 7.4 Hz, 3H). ^13^C NMR (125 MHz, DMSO-*d_6_*) *δ* 194.17, 164.74 (*J_C-F_* = 251.6 Hz), 157.50, 156.03, 137.57, 137.27, 133.55, 133.45 (*J_C-F_* = 2.8 Hz), 133.32, 133.23, 132.77×2 (*J_C-F_* = 9.4 Hz), 130.10, 130.01, 128.85, 128.69, 127.73, 123.78, 115.69×2 (*J_C-F_* = 21.9 Hz), 112.63, 104.11, 45.89, 23.17, 10.76, 7.99. MS (ESI) *m/z*: [M + H]^+^ Calcd for C_26_H_24_FN_4_O_3_: 459.1827, Found: 459.1818.

#### 3.2.28. (3. Z, 6Z)-3-(4-fluorophenoxy)benzylidene)-6-((5-isopropyl-1-(n- propyl)-imidazol-4-yl)methylene)- piperazine-2, 5-dione (**15h**)

Yield: 67%, yellow solid 75 mg, MP: 201–203 °C. ^1^H NMR (500 MHz, DMSO-*d_6_*) *δ* 12.00 (s, 1H), 10.12 (s, 1H), 7.89 (s, 1H), 7.41 (t, *J* = 7.9 Hz, 1H), 7.30-7.20 (m, 3H), 7.17 (s, 1H), 7.12 (dd, *J* = 9.0, 4.5 Hz, 2H), 6.94-6.87 (m, 1H), 6.71 (d, *J* = 8.0 Hz, 2H), 3.97 (t, *J* = 7.3 Hz, 2H), 3.31-3.18 (m, 1H), 1.71-1.67 (m, 2H), 1.32 (d, *J* = 7.1 Hz, 6H), 0.87 (t, *J* = 7.3 Hz, 3H). ^13^C NMR (125 MHz, DMSO-*d_6_*) *δ* 159.1, 157.2, 157.0, 156.8 (*J_C-F_* = 152.6 Hz), 152.6, 138.5, 137.2, 135.2, 132.1, 130.3, 127.2, 124.4, 123.7, 120.5×2 (*J_C-F_* = 8.5 Hz), 118.9, 117.7, 116.5×2 (*J_C-F_* = 23.2 Hz), 113.1, 104.2, 46.08, 24.0, 23.6, 22.6×2, 10.7. MS (ESI) *m/z*: [M + H]^+^ Calcd for C_27_H_28_FN_4_O_3_: 475.2140, Found 475.2133.

#### 3.2.29. (3. Z, 6Z)-3-(4-fluorobenzoyl)benzylidene)-6-((5-isopropyl-1-(n- propyl)-imidazol-4-yl)methylene)- piperazine-2, 5-dione (**15i**)

Yield: 57%, yellow solid 61 mg, MP: 200–202 °C. ^1^H NMR (500 MHz, DMSO-*d_6_*) *δ* 12.01 (s, 1H), 10.36 (s, 1H), 7.95–7.87 (m, 3H), 7.84 (s, 1H), 7.78 (d, *J* = 7.6 Hz, 1H), 7.63 (d, *J* = 7.7 Hz, 1H), 7.58 (t, *J* = 7.6 Hz, 1H), 7.40 (t, *J* = 8.8 Hz, 2H), 6.79 (s, 1H), 6.69 (s, 1H), 3.97 (t, *J* = 7.3 Hz, 2H), 3.21 (dt, *J* = 14.2, 7.1 Hz, 1H), 1.74-1.64 (m, 2H), 1.32 (d, *J* = 7.1 Hz, 6H), 0.88 (t, *J* = 7.4 Hz, 3H). ^13^C NMR (125 MHz, DMSO-*d_6_*) *δ* 194.18, 164.74 (*J_C-F_* = 251.6 Hz), 157.66, 156.22, 138.35, 137.25, 137.16, 133.73, 133.45 (*J_C-F_* = 2.8 Hz), 133.33, 132.76×2 (*J_C-F_* = 9.4 Hz), 132.14, 130.09, 128.80, 128.59, 128.10, 123.88, 115.69×2 (*J_C-F_* = 22.0 Hz), 112.58, 104.05, 46.09, 23.99, 23.64, 22.61×2, 10.75. MS (ESI) *m/z*: [M + Na]^+^ Calcd for C_28_H_27_FN_4_O_3_ Na: 509.1965, Found: 509.1970.

#### 3.2.30. (3. Z, 6Z)-3-(4-fluorophenoxy)benzylidene)-6-((5-isopropyl-1-(n- butyl)-imidazol-4-yl)methylene)- piperazine-2, 5-dione (**15g**)

Yield: 61%, yellow solid 90 mg, MP: 203–204 °C. ^1^H NMR (500 MHz, DMSO-*d_6_*) *δ* 12.01 (s, 1H), 10.16 (s, 1H), 7.90 (s, 1H), 7.41 (t, *J* = 7.9 Hz, 1H), 7.24 (ddd, *J* = 8.6, 7.3, 5.4 Hz, 3H), 7.17 (s, 1H), 7.14 – 7.09 (m, 2H), 6.90 (dd, *J* = 8.1, 2.0 Hz, 1H), 6.71 (s, 1H), 6.69 (s, 1H), 4.00 (t, *J* = 7.4 Hz, 2H), 3.21 (dt, *J* = 14.2, 7.1 Hz, 1H), 1.69 – 1.58 (m, 2H), 1.34 – 1.24 (m, 8H), 0.90 (t, *J* = 7.4 Hz, 3H). ^13^C NMR (125 MHz, DMSO-*d_6_*) *δ* 159.13, 157.38, 157.23, 157.04, 156.16, 152.59, 138.44, 137.17, 135.20, 132.09, 130.28, 127.23, 124.42, 123.68, 120.57, 120.50, 118.89, 117.74, 116.63, 116.44, 113.16, 104.23, 44.38, 32.71, 23.67, 22.63×2, 19.18, 13.47. MS (ESI) *m/z*: [M + Na]^+^ Calcd for C_28_H_29_FN_4_O_3_Na: 511.2116, Found 511.2108.

#### 3.2.31. (3. Z, 6Z)-3-(4-fluorobenzoyl)benzylidene)-6-((5-isopropyl-1-(n- butyl)-imidazol-4-yl)methylene)- piperazine-2, 5-dione (**15k**)

Yield: 30%, yellow solid 101 mg, MP: 198–201 °C. ^1^H NMR (500 MHz, DMSO-*d_6_*) *δ*12.01 (s, 1H), 10.34 (s, 1H), 7.95-7.88 (m, 3H), 7.84 (s, 1H), 7.78 (d, *J* = 7.5, 1H), 7.63 (d, *J* = 7.7, 1H), 7.58 (t, *J* = 7.6, 1H), 7.40 (t, *J* = 8.8, 2H), 6.79 (s, 1H), 6.69 (s, 1H), 4.01 (t, *J* = 7.3, 2H), 3.28-3.14 (m, 1H), 1.65 (dt, *J* = 14.9, 7.5, 2H), 1.38-1.25 (m, 8H), 0.91 (t, *J* = 7.4, 3H). ^13^C NMR (125 MHz, DMSO-*d_6_*) *δ* 194.19, 164.74 (*J_C-F_* = 250.1 Hz), 157.63, 156.19, 138.35, 137.26, 137.13, 133.68, 133.44, 133.33, 132.77×2 (*J_C-F_* = 9.45 Hz), 132.14, 130.09, 128.82, 128.62, 123.83, 115.70×2 (*J_C-F_* = 21.94 Hz), 112.63, 104.10, 44.37, 32.71, 23.66, 22.62*2, 19.18, 13.47. MS (ESI) *m/z*: [M + Na]^+^ Calcd for C_29_H_29_FN_4_O_3_Na: 523.2116, Found: 523.2123.

#### 3.2.32. Preparation of (3Z, 6Z)-3-(4-fluorobenzoyl)benzylidene)-6- ((5-methyl-1-(N-boc-amino-ethyl)-imidazol-4-yl) methylene) piperazine-2, 5-dione (**15l**)

Yield: 8%, yellow solid 53 mg, MP: 189–191 °C. ^1^H NMR (500 MHz, DMSO-*d*_6_) *δ* = 11.84 (s, 1H), 10.34 (s, 1H), 7.95-7.88 (m, 3H), 7.85 (d, *J* = 6.0, 1H), 7.82 (s, 1H), 7.57 (dt, *J* = 15.1, 7.6, 2H), 7.40 (t, *J* = 8.8, 2H), 7.00 (t, *J* = 5.7, 1H), 6.74 (s, 1H), 6.53 (s, 1H), 4.01 (t, *J* = 5.8, 2H), 3.22 (d, *J* = 5.9, 2H), 2.29 (s, 3H), 1.37 (s, 9H). ^13^C NMR (125 MHz, DMSO-*d_6_*) *δ* 194.25, 165.71, 163.71, 155.58, 137.59, 137.18, 133.30, 132.79, 132.72, 130.11, 128.70, 115.77, 115.60, 77.97, 43.93, 28.13×2, 16.42, 7.94. MS (ESI) *m/z*: [M + Na]^+^ Calcd for C_30_H_30_FN_5_O_5_Na: 582.2123, Found 582.2130.

#### 3.2.33. Preparation of (3Z, 6Z)-3-(4-fluorobenzoyl)benzylidene)-6- ((5-isopropyl-1-(N-boc-amino-ethyl)-imidazol-4-yl) methylene) piperazine-2, 5-dione (**15m**)

Yield: 19%, yellow solid 64 mg, MP: 204–206 °C. ^1^H NMR (500 MHz, DMSO-*d*_6_) δ = 12.00 (s, 1H), 10.35 (s, 1H), 7.91 (dt, *J* = 8.4, 4.2, 2H), 7.83 (s, 1H), 7.77 (d, *J* = 11.6, 2H), 7.63 (d, *J* = 7.7, 1H), 7.58 (t, *J* = 7.6, 1H), 7.40 (t, *J* = 8.8, 2H), 7.03 (t, *J* = 5.7, 1H), 6.79 (s, 1H), 6.70 (s, 1H), 4.05 (t, *J* = 5.8, 2H), 3.25–3.15 (m, 3H), 1.38–1.27 (m, 15H). ^13^C NMR (125 MHz, DMSO-*d_6_*) δ 194.18, 165.74, 163.74, 157.61, 156.16, 155.61, 137.32, 137.26, 133.44, 133.34, 132.81, 132.73, 131.96, 130.09, 128.83, 128.64, 115.79, 115.62, 77.99, 44.06, 28.13*3, 23.65, 22.57×2. MS (ESI) *m/z*: [M + Na]^+^ Calcd for C_32_H_34_FN_5_O_5_Na: 610.2436, Found 610.2438.

#### 3.2.34. Preparation of (3Z, 6Z)-3-(4-fluorobenzoyl)benzylidene)-6-((5-methyl-1-(N-boc- amino-propyl)-imidazol-4-yl) methylene) piperazine-2, 5-dione (**15n**)

Yield: 50%, yellow solid 260 mg, MP: 206–208 °C. ^1^H NMR (500 MHz, DMSO-*d*_6_) *δ* =11.87 (s, 1H), 10.37 (s, 1H), 7.97-7.88 (m, 3H), 7.86 (s, 1H), 7.82 (d, *J* = 7.2, 1H), 7.62 (d, *J* = 7.5, 1H), 7.57 (t, *J* = 7.6, 1H), 7.40 (t, *J* = 8.7, 2H), 6.95 (s, 1H), 6.77 (s, 1H), 6.56 (s, 1H), 3.97 (t, *J* = 6.8, 2H), 2.93 (d, *J* = 5.8, 2H), 2.30 (s, 3H), 1.90-1.70 (m, 2H), 1.39 (s, 9H). ^13^C NMR (125 MHz, DMSO-*d_6_*) *δ* 194.20, 165.73, 163.72, 157.67, 156.20, 155.63, 137.48, 137.23, 133.81, 133.46, 133.38, 133.31, 132.80, 132.73, 130.11, 129.77, 128.79, 128.52, 124.09, 115.78, 115.60, 112.39, 103.72, 77.69, 42.05, 36.93, 29.98, 28.22×3, 7.94. MS (ESI) *m/z*: [M + H]^+^ Calcd for C_31_H_33_FN_5_O_5_: 574.2460, Found 574.2446. 

#### 3.2.35. Preparation of (3Z, 6Z)-3-(4-fluorobenzoyl)benzylidene)-6- ((5-isopropyl-1-(N- boc-amino-propyl)-imidazol-4-yl) methylene) piperazine-2, 5-dione (**15o**)

Yield: 44%, yellow solid 200 mg, MP: 179–180 °C. ^1^H NMR (500 MHz, DMSO-*d*6) δ = 12.01 (s, 1H), 10.35 (s, 1H), 7.91 (s, 3H), 7.82 (s, 1H), 7.75 (d, *J* = 6.5, 1H), 7.66-7.56 (m, 2H), 7.40 (s, 2H), 6.98 (s, 1H), 6.81 (s, 1H), 6.69 (s, 1H), 4.02 (s, 2H), 3.20 (s, 1H), 2.95 (s, 2H), 1.79 (s, 2H), 1.47-1.24 (m, 15H). ^13^C NMR (125 MHz, DMSO-*d_6_*) δ 194.17, 165.74, 163.74, 157.51, 156.09, 155.64, 138.35, 137.28, 137.21, 133.53, 133.43, 133.34, 132.81, 132.73, 132.16, 130.09, 128.85, 128.72, 127.62, 123.77, 115.79, 115.61, 112.81, 104.18, 77.69, 42.39, 37.02, 30.91, 28.22×3, 23.62, 22.53×2. MS (ESI) *m/z*: [M + Na]^+^ Calcd for C_33_H_36_FN_5_NaO_5_^+^: 624.2593, Found 624.2590.

#### 3.2.36. Preparation of (3Z, 6Z)-3-(4-fluorobenzoyl)benzylidene)-6- ((5-isopropyl-1-allyl-imidazol-4-yl) methylene) piperazine-2, 5-dione (**15p**)

Yield: 75%, yellow solid 115 mg, MP: 200–202 °C. ^1^H NMR (500 MHz, DMSO-d_6_) *δ*11.99 (s, 1H), 10.36 (s, 1H), 7.94-7.89 (m, 2H), 7.88 (s, 1H), 7.82 (s, 1H), 7.75 (d, *J* = 7.52 Hz, 1H), 7.64 (d, *J* = 7.63 Hz, 1H), 7.59 (t, *J* = 7.63 Hz, 1H), 7.40 (t, *J* = 8.74 Hz, 2H), 6.81 (s, 1H), 6.71 (s, 1H), 6.01 (ddd, *J* = 4.97, 10.23, 15.29 Hz, 1H), 5.22 (d, *J* = 10.32 Hz, 1H), 4.94 (d, *J* = 17.16 Hz, 1H), 4.73 (s, 2H), 3.15 (dt, *J* = 7.05, 14.20 Hz, 1H), 1.30 (d, *J* = 7.07 Hz, 6H). ^13^C NMR (125 MHz, DMSO-*d_6_*) *δ* 194.16, 165.75, 163.74, 157.50, 156.12, 138.61, 137.38, 137.28, 134.31, 133.53, 133.45, 133.43, 133.34, 132.81, 132.73, 132.25, 130.10, 128.85, 128.73, 127.62, 123.89, 116.78, 115.79, 115.61, 112.85, 104.12, 46.83, 23.72, 22.47*2. MS (ESI) *m/z*: [M + Na]^+^ Calcd for C_28_H_25_FN_4_O_3_Na: 507.1803, Found 507.1812.

#### 3.2.37. Preparation of (3Z, 6Z)-3-(4-fluorobenzoyl)benzylidene)-6- ((5-isopropyl-1-(propargyl)-imidazol-4-yl) methylene) Piperazine-2, 5-dione (**15q**)

Yield: 72%, yellow solid 166 mg, MP: 150–152 °C. ^1^H NMR (500 MHz, DMSO-d_6_) *δ*11.90 (s, 1H), 10.38 (s, 1H), 7.92 (dd, *J* = 9.14, 17.20 Hz, 3H), 7.82 (s, 1H), 7.76 (d, *J* = 7.43 Hz, 1H), 7.64 (d, *J* = 7.54 Hz, 1H), 7.59 (t, *J* = 7.58 Hz, 1H), 7.40 (t, *J* = 8.70 Hz, 2H), 6.82 (s, 1H), 6.69 (s, 1H), 5.02 (s, 2H), 3.58 (s, 1H), 3.37-3.25 (m, 1H), 1.35 (d, *J* = 7.03 Hz, 6H). ^13^C NMR (125 MHz, DMSO-*d_6_*) *δ* 194.29, 165.84, 163.84, 157.56, 156.25, 138.41, 137.36, 137.05, 133.56, 133.44, 132.90, 132.82, 132.61, 130.17, 128.97, 128.88, 127.62, 124.27, 115.88, 115.70, 113.13, 103.93, 78.52, 76.63, 34.59, 23.92, 22.32×2. MS (ESI) *m/z*: [M + Na]^+^ Calcd for C_28_H_23_FN_4_O_3_Na: 505.1646, Found 505.1646.

#### 3.2.38. Preparation of (3Z, 6Z)-3-(4-fluorobenzoyl)benzylidene)-6- ((5-methyl-1-(amino-ethyl)-imidazol-4-yl) methylene) Piperazine-2, 5-dione (**16a**)

Yield: 45%, yellow solid 14 mg, MP: 180–182 °C. ^1^H NMR (500 MHz, DMSO-*d*_6_) *δ* = 11.70 (s, 1H), 10.38 (s, 1H), 8.36 (s, 3H), 8.17 (s, 1H), 7.94-7.88 (m, 2H), 7.82 (s, 1H), 7.75 (d, *J* = 7.4, 1H), 7.64 (d, *J* = 7.5, 1H), 7.59 (t, *J* = 7.6, 1H), 7.40 (t, *J* = 8.6, 2H), 6.82 (s, 1H), 6.58 (s, 1H), 4.32 (t, *J* = 6.3, 2H), 3.20 (d, *J* = 5.6, 2H), 2.34 (s, 3H). ^13^C NMR (125 MHz, DMSO-*d_6_*) *δ* 195.10, 166.35, 164.35, 158.15, 156.86, 138.20, 137.79, 133.92, 133.84, 133.51, 133.37, 133.30, 131.17, 130.45, 129.66, 129.60, 127.90, 124.57, 116.37, 116.20, 113.95, 104.79, 42.43, 8.51. MS (ESI) *m/z*: [M + Na]^+^ Calcd for C_25_H_22_FN_5_O_3_Na: 482.1599, Found 482.1601.

#### 3.2.39. Preparation of (3Z, 6Z)-3-(4-fluorobenzoyl)benzylidene)-6- ((5-isopropyl-1-(amino-ethyl)-imidazol-4-yl) methylene) Piperazine-2, 5-dione (**16b**)

Yield: 77%, yellow solid 24 mg, MP: 190–192 °C. ^1^H NMR (500 MHz, DMSO-*d*_6_) δ = 11.89 (s, 1H), 10.40 (s, 1H), 8.31 (s, 3H), 8.06 (s, 1H), 7.91 (dd, *J* = 8.7, 5.6, 2H), 7.82 (s, 1H), 7.75 (d, *J* = 7.7, 1H), 7.64 (d, *J* = 7.7, 1H), 7.59 (t, *J* = 7.6, 1H), 7.41 (t, *J* = 8.8, 2H), 6.82 (s, 1H), 6.70 (s, 1H), 4.34 (t, *J* = 6.7, 2H), 3.26 (dt, *J* = 14.2, 7.1, 1H), 3.15 (dd, *J* = 12.0, 6.0, 2H), 1.33 (d, *J* = 7.1, 6H). ^13^C NMR (125 MHz, DMSO-*d_6_*) δ 194.18, 165.76, 163.76, 157.42, 156.20, 138.38, 137.29, 133.47, 133.38, 132.82, 132.74, 130.08, 128.87, 128.81, 127.53, 115.81, 115.64, 113.11, 103.72, 41.95, 23.48, 22.44×2. MS (ESI) *m/z*: [M + Na]^+^ Calcd for: C_27_H_26_FN_5_O_3_Na, 510.1912, Found 510.1908.

#### 3.2.40. Preparation of (3Z, 6Z)-3-(4-fluorobenzoyl)benzylidene)-6- ((5-methyl-1-(amino-propyl)-imidazol-4-yl) methylene) piperazine-2, 5-dione (**16c**)

Yield: 85%, yellow solid 82 mg, MP: 247–248 °C. ^1^H NMR (500 MHz, DMSO-*d*_6_) *δ* = 11.65 (s, 1H), 10.39 (s, 1H), 8.27 (s, 1H), 8.20 (s, 3H), 7.91 (dd, *J* = 8.7, 5.6, 2H), 7.82 (s, 1H), 7.75 (d, *J* = 7.7, 1H), 7.64 (d, *J* = 7.7, 1H), 7.59 (t, *J* = 7.6, 1H), 7.40 (t, *J* = 8.8, 2H), 6.82 (s, 1H), 6.57 (s, 1H), 4.16 (t, *J* = 7.0, 2H), 2.80 (dd, *J* = 12.9, 6.6, 2H), 2.32 (s, 3H), 2.08–1.98 (m, 2H). ^13^C NMR (125 MHz, DMSO-*d_6_*) *δ* 194.17, 165.75, 163.75, 157.33, 156.33, 137.29, 137.08, 133.47, 133.37, 132.82, 132.75, 130.12, 129.87, 128.88, 128.79, 127.59, 115.80, 115.63, 113.10, 103.00, 41.98, 35.89, 27.56, 8.19. MS (ESI) *m/z*: [M + Na]^+^ Calcd for C_26_H_24_FN_5_O_3_ Na: 496.1755, Found 496.1768.

#### 3.2.41. Preparation of (3Z, 6Z)-3-(4-fluorobenzoyl)benzylidene)-6- ((5-isopropyl-1-(amino-propyl)-imidazol-4-yl) methylene) piperazine-2, 5-dione (**16d**)

Yield: 78%, yellow solid 310 mg, MP: 199–201 °C. ^1^H NMR (500 MHz, DMSO-*d*_6_) δ = 11.82 (s, 1H), 10.41 (s, 1H), 8.17 (s, 3H), 7.93–7.86 (m, 2H), 7.81 (s, 1H), 7.75 (d, *J* = 7.7, 1H), 7.64 (d, *J* = 7.7, 1H), 7.58 (t, *J* = 7.6, 1H), 7.43–7.37 (m, 2H), 6.81 (s, 1H), 6.67 (d, *J* = 3.6, 1H), 4.19 (dt, *J* = 14.5, 7.4, 2H), 3.28–3.19 (m, 1H), 2.81 (dd, *J* = 13.3, 6.5, 2H), 2.04–1.95 (m, 2H), 1.32 (dd, *J* = 7.0, 2.4, 6H). ^13^C NMR (125 MHz, DMSO-*d_6_*) δ 194.17, 165.75, 163.75, 157.37, 156.29, 138.27, 137.29, 136.78, 133.47, 133.38, 132.82, 132.74, 130.10, 128.87, 128.80, 127.53, 115.81, 115.63, 113.16, 42.08, 36.01, 28.55, 23.52, 22.47×2. MS (ESI) *m/z*: [M + Na]^+^ Calcd for C_28_H_28_FN_5_O_3_Na: 524.2068, Found 524.2071.

### 3.3. Biology

#### 3.3.1. Anticancer Activities

Human cancer cell lines (lung cancer NCI-H460 cell line, pancreatic cancer BxPC-3 cell line, colon cancer HT-29 cell line) were purchased from American Type Cell Culture Collection (ATCC, Manassas, VA, USA). Cells were maintained in RPMI-1640 medium supplemented with 10% (*v/v*) heat-inactivated fetal bovine serum, penicillin-streptomycin (100 U/mL-100 g/mL) at 37 °C in a humidified atmosphere (5% CO_2_–95% air). All tested derivatives were dissolved in 100% cell culture grade DMSO. Cells (3 × 10^3^ per well) were seeded in 96-well plates for 24 h. Then, cells were treated with the tested compounds in six concentrations for 72 h. Cell viability was assessed by MTT assay. The absorbance at 490 nm was measured with a Microplate Reader (Molecular Devices, Silicon Valley, CA, USA). The data were analyzed by Origin 8.5 (OriginLab, Northampton, MA, USA) [17,20].

#### 3.3.2. Immunofluorescence Assay

NCI-H460 cells were seeded in 24-well plate (with coverslips plated) at the density of 5 × 10^4^ cells. After overnight adherence, the cells were exposed to compounds at 5 nM or 10 nM for 24 h, respectively. Then, cells were fixed with 4% cold paraformaldehyde at 4 °C for 15 min and then permeabilized in 0.05% Triton X-100 for 10 min. The cells were blocked in 1% BSA for 30 min. Microtubules were detected by incubation with a monoclonal anti-β-tubulin (β-tubulin antibody was obtained from Servicebio, Wuhan, China.) at 4 °C for 12 h. Subsequently, the cells were washed with PBS and incubated with a FITC-conjugated anti-mouse IgG antibody. Nuclei were stained with DAPI (G1012, Servicebio, Wuhan, China). The coverslips were visualized under fluorescence microscope (Nikon Eclipse C1, Nikon, Japan) and an image-forming system (Nikon DS-U3, Nikon, Tokyo, Japan) [17,21].

#### 3.3.3. Western Blotting Test

Western blot was performed as previously described [22] with antibody caspase-3 (1:1000) (WB0177/WB0176) or GAPDH (1:3000) (WB0197/WB0196, Shanghai Wei-Ao Biotechnology Co., Shanghai, China). Densities of the Western blotting bands were quantified using ImageJ 19.0. (NIH, Bethesda, MD, USA).

### 3.4. Molecular Modeling

Ligands were prepared by using the QuickPrep module in Maestro and energy minimized through general method. The X-ray crystallographic structure was retrieved from the protein data bank (PDB: 5XHC) at a resolution of 2.75 Å. The protein domain subunits C, D and water molecules were removed using Maestro 11.5 using the protein preparation refinement module. A subsequent energy minimization was carried out using the OPLS_2005 force field. Then, molecules were docked into the co-crystal structure of tubulin-compound **1** [17].

The docking position was constrained by hydrogen bond using receptor grid generation module. Molecular modeling was completed by ligand docking module according to the import of the pretreatment ligand and protein. At least five poses were retained for each compounds **15a**–**15q** and **16a**–**16d**, and the best rigid docking and induced fit docking poses were refined.

### 3.5. In Vitro Pharmacokinetics Study 

Compounds **15p**. **15p** were weighed and dissolved in DMSO to prepare a master batch with a concentration of 1 mg/mL. The plasma was obtained from wistar male rats, and stored at −20 °C for pending use. The 10 μmol/L of the sample solution to be tested was vortexed and mixed, and rapidly incubated in a 37 °C water bath shaker. The 100 μL of the incubation system was removed at 0 min, 5 min, 10 min, 20 min, 40 min, 60 min, 90 min, and 120 min of the reaction, respectively. The remaining amount of compound was detected (note: each sample was operated three times in parallel). The concentrations of the samples to be measured (**15p**) in rat plasma were detected by HPLC-MS/MS, and the experimental data were processed and plotted using the computer programs Microsoft Excel (Microsoft 97–2003, Redmond, Washington, DC, USA) and Origin 8.5 (OriginLab, Northampton, MA, USA).

#### Stability of Liver Microsomal Metabolism

The total volume of the incubation system is 1 mL, which contains 1 mmol/L NADPH, 0.5 mg/mL rat liver microsomes, 1 μmol/L **15p** standard solution, which was supplemented with K_2_HPO_4_ buffer to 1 mL. The solution should be added to the incubation system. The incubation system was rapidly incubated in a water bath shaker at 37 °C for 5 min before adding 200 μL of 5 mmol/L NADPH. The 50 μL of the incubation system was quickly removed at 0, 5, 10, 20, 40, 60, 90, 120, 150, 210, and 270 min. The concentrations of the samples to be measured (**15p**) in rat liver microsomes were measured by HPLC-MS/MS. Each sample was operated on three times in parallel. The experimental data were processed and plotted using the computer programs Microsoft Excel (97–2003) and Origin 7.5 software.

### 3.6. In Vivo Pharmacodynmic Study

The literature was referenced for this part of the experiment [20].

Balb/c mice (4–6 weeks old, 16–18 g) were purchased from Shanghai Sippr-BK laboratory animal Co. Ltd. The animal study protocol was approved by Institutional Review Board of Science and Technology Division of Linyi University (review opinion on behalf of the Ethics Committee) (The protocol code is LYU20220107, provided on 10 March 2022). H22 cells were grown in RPMI-1640 midium supplemented with 10% fetal bovine serum, and maintained at 37 °C in humidified 5% CO_2_. Cells were passaged when they reached 70–80% confluence. Viable H22 cells (1 × 10^7^ cells/0.2 ml 0.9% sodium chloride) were injected into the peritoneal cavity of BALB/C mice to trigger ascitic cell growth. Later, cells were harvested and suspended. H22 cancer cells (2 × 10^7^ cells/0.1 ml 0.9% sodium chloride cells) were subcutaneously injected into the right flank of each mouse. After implantation, the tumor size was measured with an electronic caliper twice a week, and the tumor volume was calculated according to the following formula: tumor volume (mm^3^) = 0.5 × length × width^2^. When the tumor volume reached about 130 mm^3^, the xenograft tumor-bearing BALB/C mice were randomly placed into six groups at 10 mice per group: vehicle, plinabulin (4 mg/kg), **15p** (12 mg/kg), **15p** (6 mg/kg), cyclophosphamide (20 mg/kg) and docetaxel (10 mg/kg) groups. Docetaxel, plinabulin, and cyclophosphamide were chosen as positive control drug. The reference compound docetaxel and cyclophosphamide were completely dissolved in isotonic saline. Plinabulin and **15p** concentrated solution were made by dissolving 16 mg compound in propylene glycol (2.4 g) and solutol-HS15 (1.6 g) due to its relatively lower solubility. The solution of compound **15p** was diluted with the concentrated solution and in isotonic saline at a calculated ratio. The solutions of plinabulin were made up according to similar methods. The mice received intravenous administration (iv) every two or three days for consecutive 14 days. The tumor volumes and the body weights were recorded every two or three days after treatment. At the end of the administration period, the animals were euthanized by dislocation, and the tumor bulks were peeled off conforming to the Guide for the Animal use and Management of Shanghai Medicilon Biological Medicine Co. Ltd. The tumor volume was calculated according to the Principles of Non-clinical Research Techniques for Anti-tumor Drugs of Cytotoxic Drugs by China Food and Drug Administration.

## 4. Conclusions

In summary, we designed and synthesized a total of 21 novel phenylahistin derivatives based on the co-crystal structure of tubulin with compound **1**. Based on the SAR study, we found that the appropriate substitute groups, i.e., 3-4 carbon atoms carbon chains and unsaturated groups, at 1-position of 1,3-imidazol-2-yl were optimal for improving the activity of the novel allyl-imidazole-type derivatives. We found that the increased chain length of the 1,3-imidazol-2-yl will decrease its activity. Among the derivatives, **15p** with an imidazole-allyl group displayed potent cytotoxicity against several human cancer cell lines, which could effectively inhibit tubulin polymerization observed in immunofluorescent assays. Compound **15p** and **1** could overlap, and the allyl of **15p** group pointed in the plane in the docking model. In vivo activity tests showed that compound **15p** was effective in inhibiting tumor growth at 4 mg/kg dose, with an inhibition rate of 65%. The effect on mice body weight was low and superior to that of docetaxel and cyclophosphamide. Therefore, derivative **15p** with an allyl-imidazole group can be considered a potential agent for the treatment of cancer. Subsequent pharmaceutical studies in vivo are currently ongoing.

## Figures and Tables

**Table 1:** Synthetic exploration conditions of compound **13d**.

Entry	Temp.	Base	Solv.	Remaining of 11b	Yield
1	0 °C	NaH	DMF	62%	-
2	rt	NaH	DMF	59%	-
3	rt	DIPEA	DMF	82%	-
4	rt	TEA	DMF	91%	-
5	rt	Cs_2_CO_3_	DMF	0%	68%

**Table 2:** Partial Hydrogen spectrum data of **13d** NMR.

Hydrogen Type	Chemical Shifts (ppm)	Peak Type	H Number	Number of Protons
N-H	12.02	s	NH-5	1
C-H	7.45	s	H-11	1
C-H	7.05	s	H-7	1
C-H	4.46	s	H-1	2
C-H	3.99	q	H-19	2
C-H	3.16	septet	H-15	1
C-H	2.64	s	H-16	3
C-H	1.45	t	H-20	3
C-H	1.41	d	H-17,18	6

**Table 3:** Partial carbon spectrum data of **13d** NMR.

C Number	Chemical Shifts (ppm)
C-8	133.28
C-9	139.34
C-11	135.72

**Table 4:** Biological activities of the synthesized plinabulin C-ring derivatives **15a**–**15q** and **16a**–**16d**.

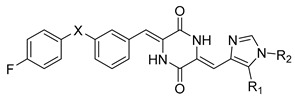						
Compds	X	R1	R2	IC_50_ (nM) *^a^* NCI-H460	IC_50_ *^a^* (nM)/BxPC-3	IC_50_ (nM)/HT-29
Plinabulin				26.2 ± 3.2	5.8 ± 0.2	6.6 ± 1.6
**15a**	C=O	Methyl	Methyl	21.1 ± 1.30	-	-
**15b**	O	Isopropyl	Methyl	40.40 ± 0.82	-	-
**15c**	C=O	Isopropyl	Methyl	3.65 ± 0.18	-	-
**15d**	C=O	Methyl	Ethyl	16.90 ± 1.50	-	-
**15e**	O	Isopropyl	Ethyl	12.20 ± 0.27	-	-
**15f**	C=O	Isopropyl	Ethyl	2.01 ± 0.19	-	-
**15g**	C=O	Methyl	n-Propyl	4.93 ± 0.25	-	-
**15h**	O	Isopropyl	n-Propyl	5.40 ± 0.15	-	-
**15i**	C=O	Isopropyl	n-Propyl	1.67 ± 0.15	-	-
**15j**	O	Isopropyl	n-Butyl	2.49 ± 0.28	1.13 ± 0.35	2.11 ± 0.09
**15k**	C=O	Isopropyl	n-Butyl	0.94 ± 0.11	-	-
**15l**	C=O	Methyl	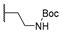	104.78 ± 11.72	-	-
**16a**	C=O	Methyl	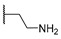	33.4 ± 9.59	-	-
**15m**	C=O	Isopropyl	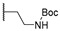	27.26 ± 1.32	-	-
**16b**	C=O	Isopropyl	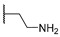	11.32 ± 3.45	3.27 ± 0.09	5.92 ± 0.39
**15n**	C=O	Methyl	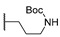	145.72 ± 0.27	-	-
**16c**	C=O	Methyl	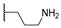	102.76 ± 11.67	-	-
**15o**	C=O	Isopropyl	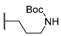	12.70 ± 0.71	-	-
**16d**	C=O	Isopropyl	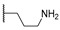	5.38 ± 1.46	2.35 ± 1.75	2.45 ± 0.42
**15p**	C=O	Isopropyl	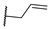	1.03 ± 0.18	0.81 ± 0.02	0.67 ± 0.064
**15q**	C=O	Isopropyl	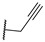	1.49 ± 0.31	1.15 ± 0.042	0.67 ± 0.035

*^a^* Values represent mean ± SD from three independent dose response curves with NCI-H460 cancer cell line. “-” indicates no data.

**Table 5:** Docking and theoretical calculations of the synthesized plinabulin C-ring derivatives **15a**–**15q** and **16a**–**16d**.

Compds	LogPo/w	PCaco	Docking Score	Compds	LogPo/w	PCaco	Docking Score
Plinabulin	2.48	377.79	11.68	**15k**	4.93	197.58	−14.16
**15a**	3.25	335.39	−14.11	**15l**	4.59	57.67	−10.16
**15b**	4.61	571.11	−14.46	**16a**	2.44	20.93	−14.56
**15c**	3.34	137.94	−14.55	**15m**	4.88	115.99	-
**15d**	3.57	336.58	−14.17	**16b**	2.71	28.92	-
**15e**	4.85	721.47	−13.91	**15n**	4.34	58.49	-
**15f**	4.20	194.15	−14.30	**16c**	2.13	25.87	−15.01
**15g**	3.89	190.86	−14.66	**15o**	5.22	64.30	-
**15h**	5.22	599.60	−14.11	**16d**	3.06	19.89	-
**15i**	4.58	201.73	−14.72	**15p**	4.51	200.27	−14.88
**15j**	5.55	506.41	−13.53	**15q**	4.35	200.28	−15.34

“-” indicates no data.

**Table 6:** Calculated pharmacokinetic parameters of liver microsomes for compound **15p**.

Parameters	15p
t_1/2_ (min)	7.07
CL_int_ (mL/min/kg)	771.91
CL (mL/min/kg)	80.60

Among them: the incubation volume is 1 mL, and the mass of liver microsomes is 0.5 mg. t_1/2_ = halflife, CLint = intrinsic Clearance, CL = total clearance.

**Table 7:** Effect of compound **15p** on tumor volume and tumor weight in mouse chemotherapy model with subcutaneous transplanted tumor of H22 hepatoma carcinoma cells (mean ± SD).

Group	Dose (mg/kg)	Tumor Volume (D1, mm^3^)	Tumor Volume (D15, mm^3^)	Tumor Weight (g)	IR (%)
Control	--	103.3 ± 32.23	1861.4 ± 890.1	1.13 ± 0.58	--
Docetaxel	10	103.6 ± 30.75	903.6 ± 421.7*	0.55 ± 0.22*	51.6
Plinabulin	4	104.7 ± 26.66	1425.3 ± 411.2	0.77 ± 0.31	31.9
Cyclophosphamide	20	105.1 ± 30.26	687.2 ± 288.6*	0.42 ± 0.24*	62.6
Compound **15p**	2	103.7 ± 29.99	1453.6 ± 909.5	0.82 ± 0.47	27.6
Compound **15p**	4	102.2 ± 28.62	674.5 ± 274.0*	0.39 ± 0.22*	65.2

Notes: * *p* < 0.05, compared with the control group. Tumor weight inhibition rate: IR (%) = (1 − TWt/TWc) × 100%, TWt: tumor weight of the treatment group; TWc: tumor weight of the model control group.

## Data Availability

Not applicable.

## Supplementary Materials

The following are available online https://www.mdpi.com/article/10.3390/md20120752/s1, see the Supporting Information, page Figures S1–S86: ^1^H NMR, ^13^C NMR and HRMS spectra of compounds in the article.

## Abbreviations

HRMS	high resolution mass spectrometer
^1^HNMR	^1^H-nuclear magnetic resonance
^13^CNMR	^13^C-nuclear magnetic resonance
HMBC	^1^H detected heteronuclear multiple bond correlation
HMQC	heteronuclear Multiple Quantum Coherence
DKP	diketopiperazine
DBU	1,8-diazabicyclo[5.4.0]undec-7-ene
TEA	Triethylamine
DMF	N,N-dimethylformamide
HT-29	human colon cancer cell
BxPC-3	biopsy xenograft of pancreatic cell
NCI-H460	human lung cancer cell
H22	mouse liver cancer cells
Mp	melting point
