# Supplementary material for: Design and Synthesis of Novel Phenylahistin Derivatives Based on Co-Crystal Structures as Potent Microtubule Inhibitors for Anti-Cancer Therapy
Source: Mar Drugs. 2022 Nov 29;20(12):752. doi: 10.3390/md20120752 (PMC9785606; doi:10.3390/md20120752)

Supporting Information for Design and Synthesis of Novel Marine Phenylahistin Derivatives Based on Co-Crystal Structure as Potent Microtubule Inhibitors for Anti-Cancer Therapy

**Contents**	pages
NMR/HRMS spectra of Compounds	S2-S44

NMR/HRMS spectra

Figure S1. ^{1}H NMR spectrum for **6** (500 MHz, $CDCl_3$)

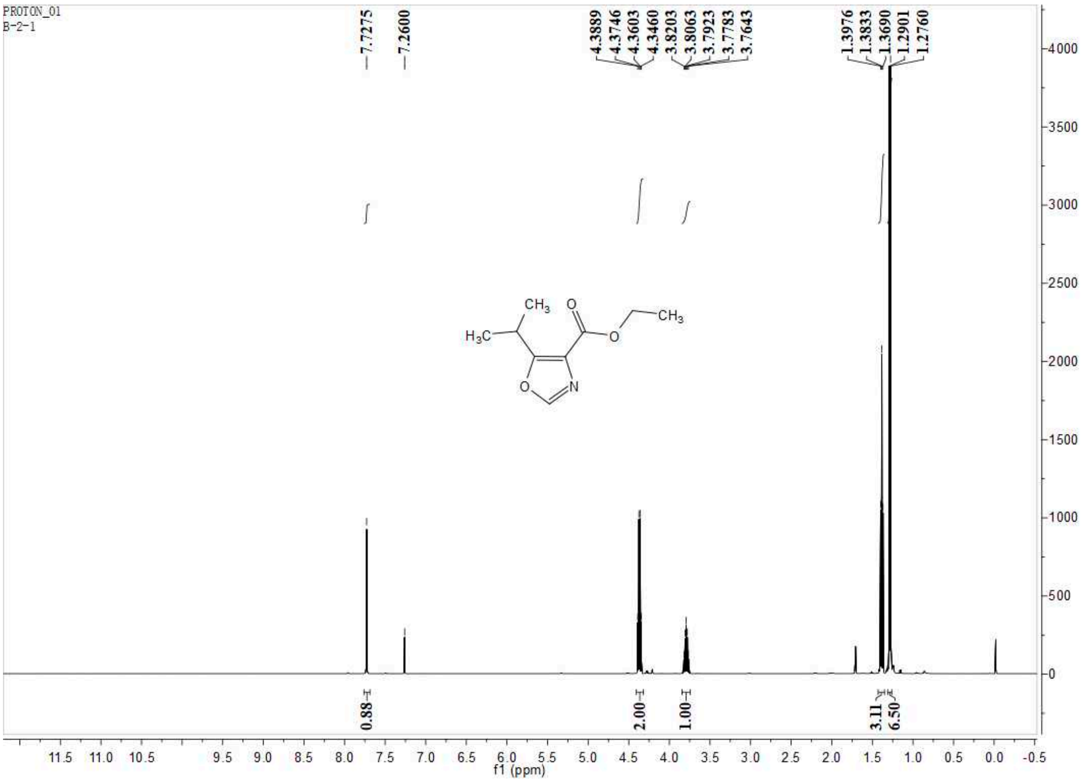

Figure S2. ^{1}H NMR spectrum for **7** (500 MHz, $CDCl_3$)

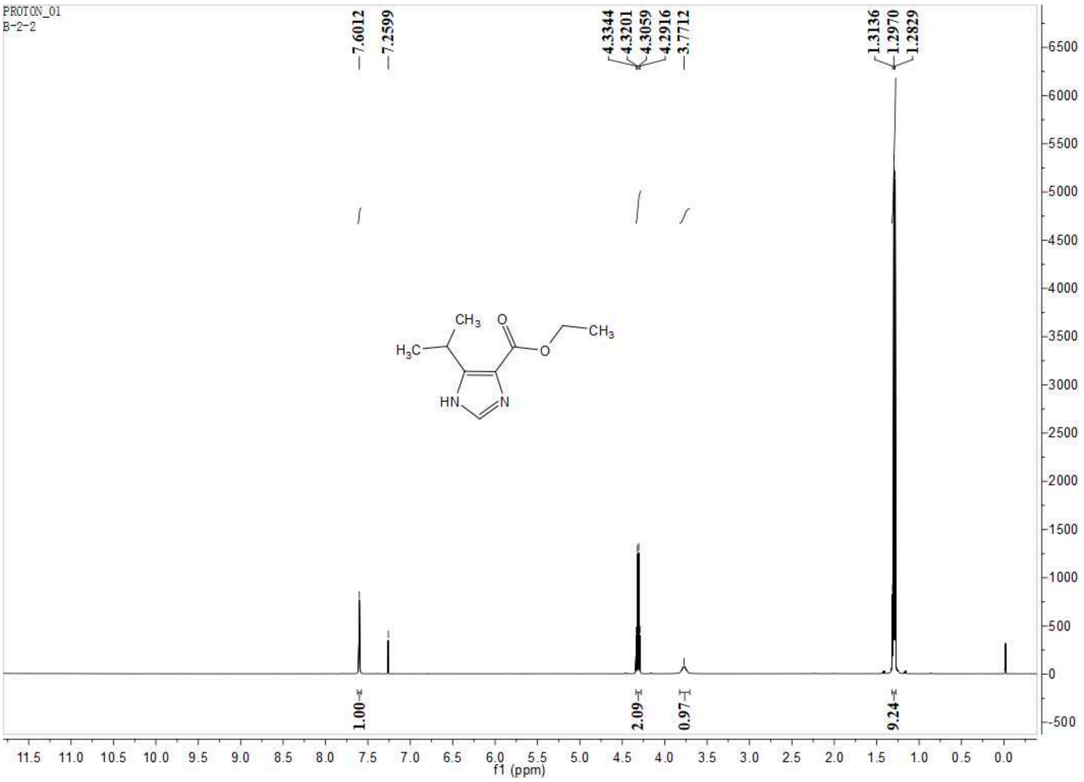

Figure S3. ^{1}H NMR spectrum for **9b** (500 MHz, $CDCl_3$)

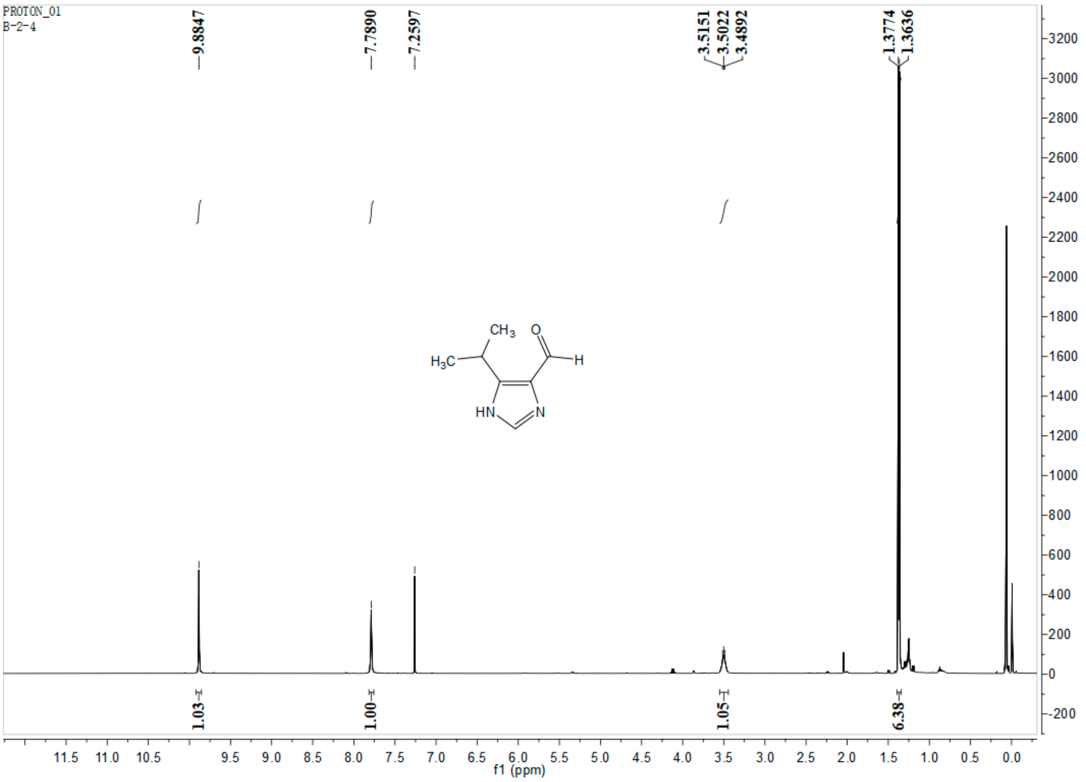

Figure S4. ^{1}H NMR spectrum for **11a** (500 MHz, DMSO-*d*$_6$)

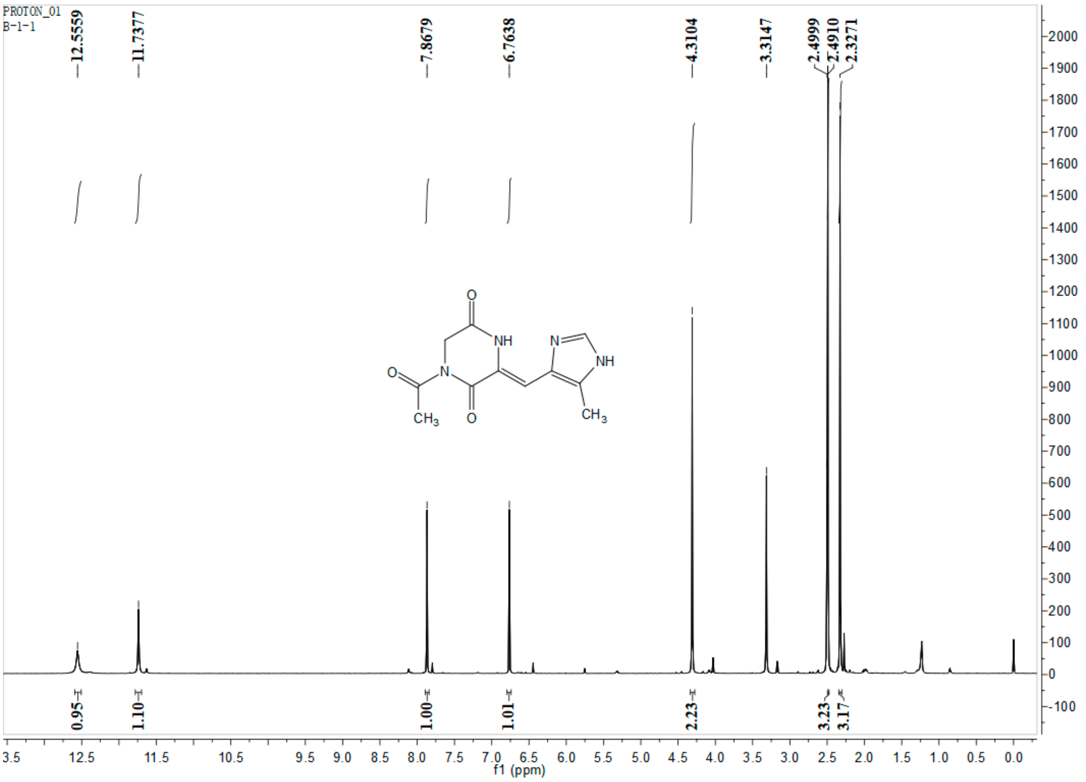

Figure S5. ^{1}H NMR spectrum for **11b** (500 MHz, DMSO-d_6)

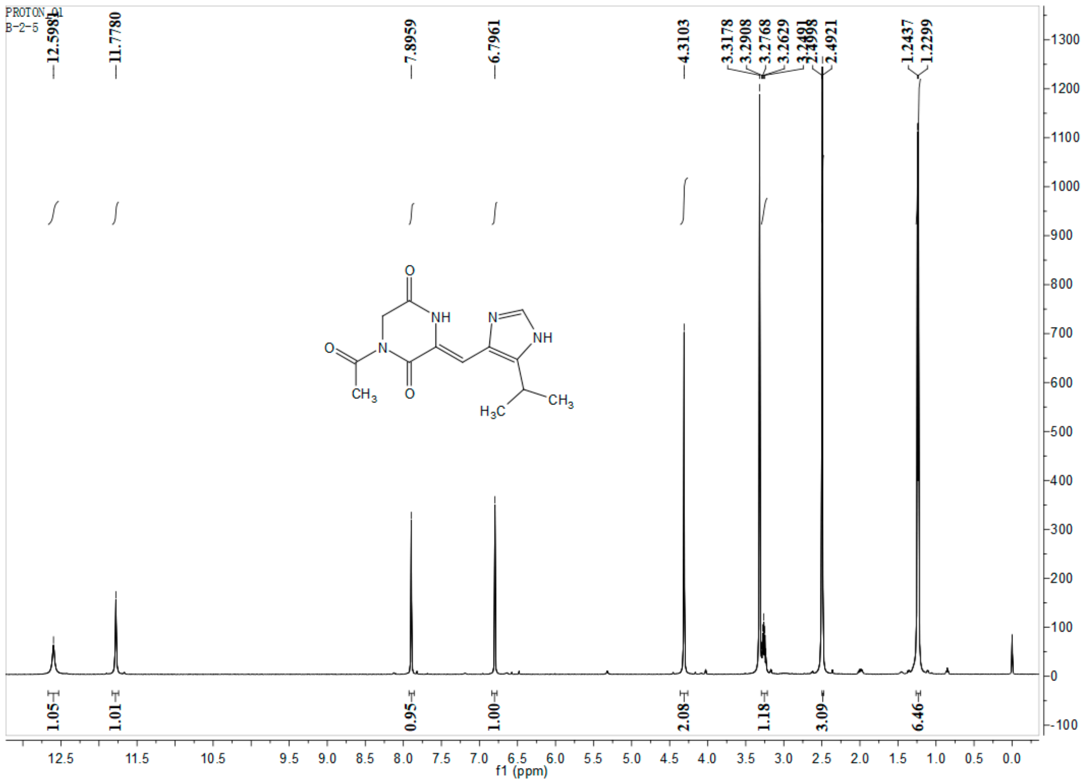

Figure S6. ^{1}H NMR spectrum for **14a** (500 MHz, $CDCl_3$)

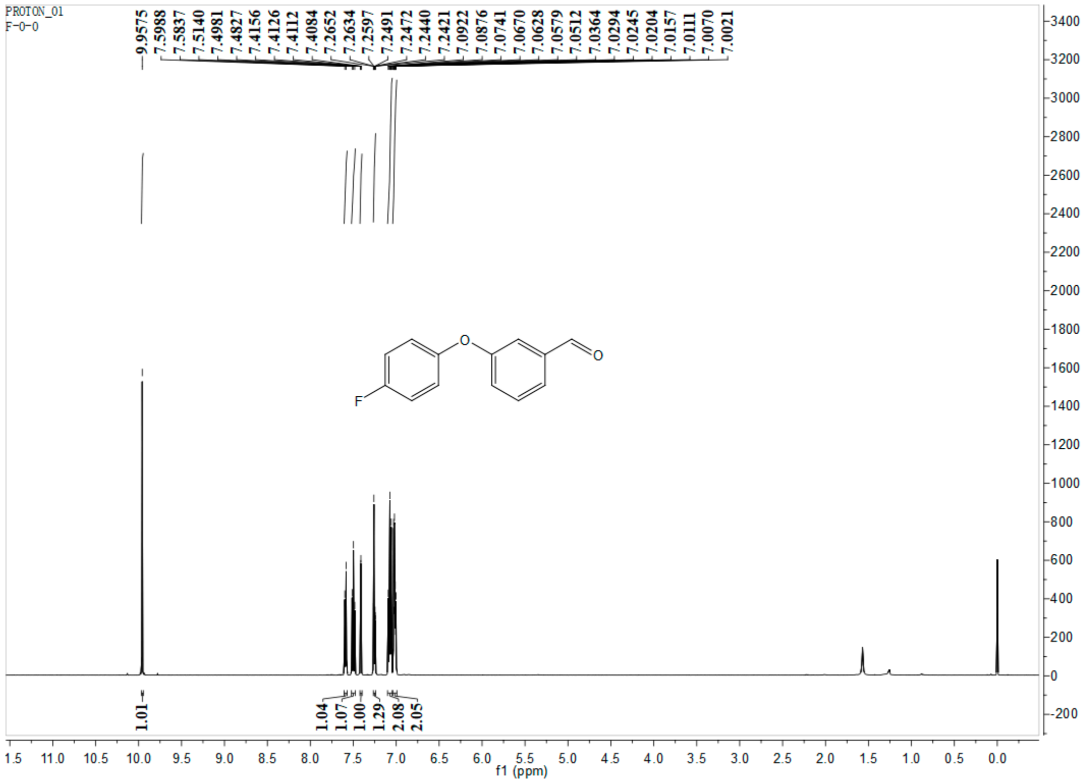

Figure S7. ^{1}H NMR spectrum for **14b** (500 MHz, $CDCl_3$)

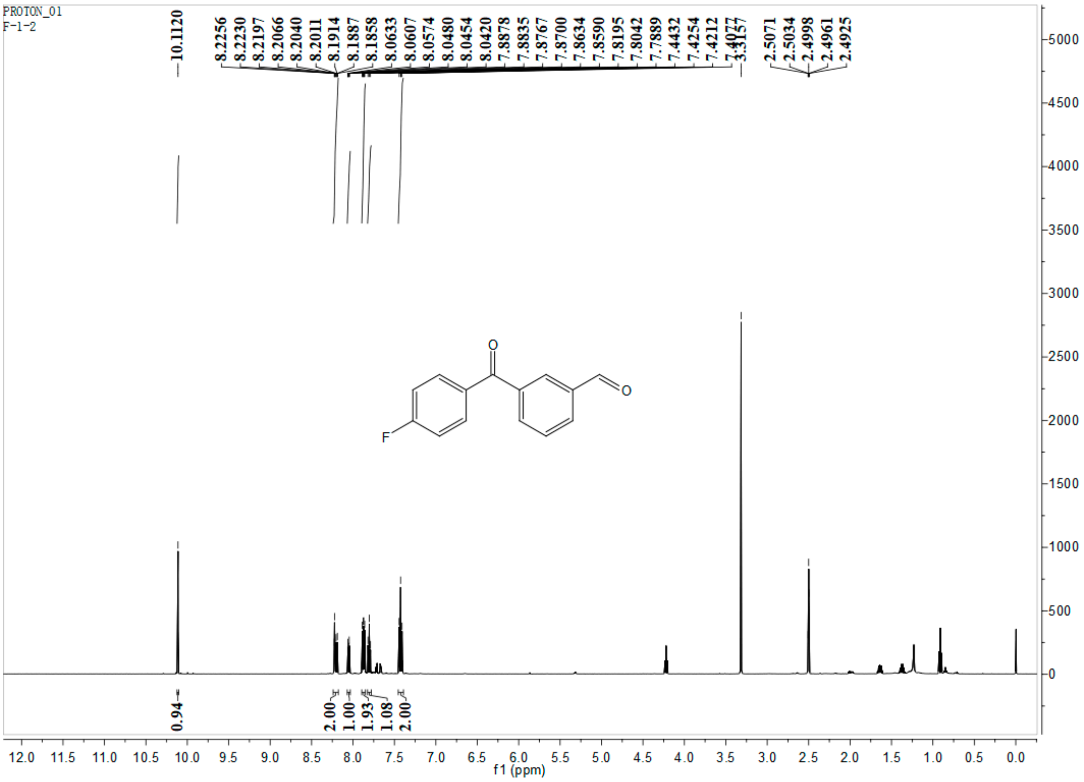

Figure S8. ^{1}H NMR spectrum for **13a** (500 MHz, $CDCl_3$)

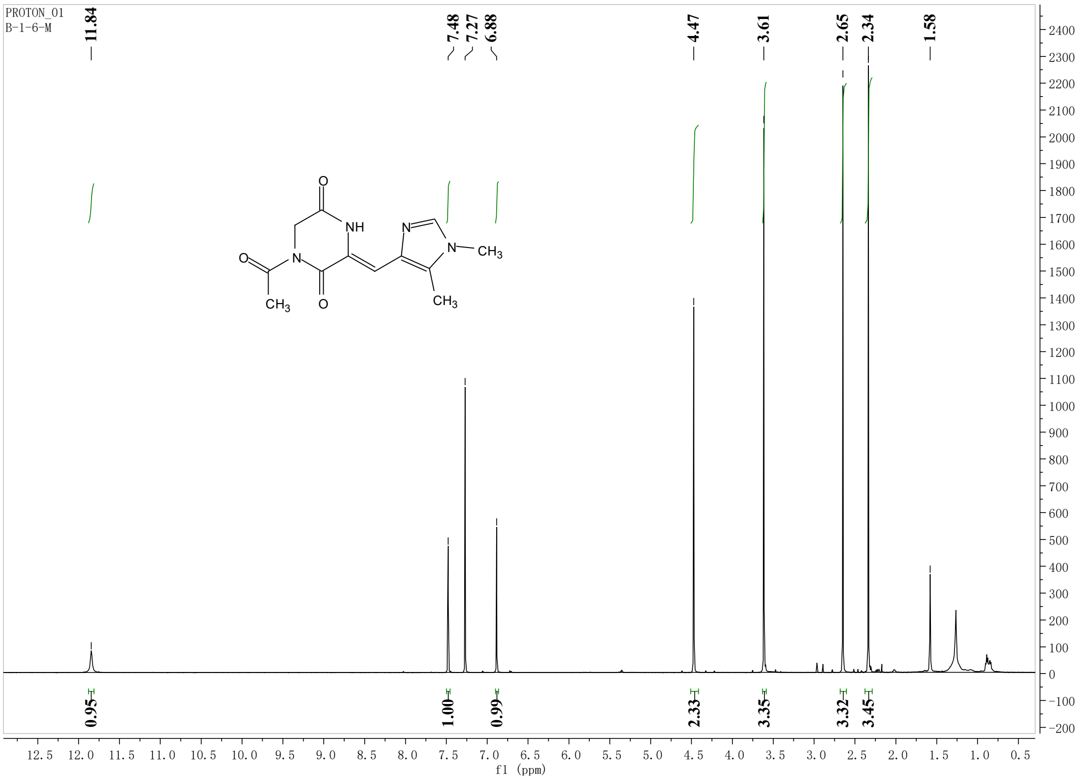

Figure S9. ^{1}H NMR spectrum for **13b** (500 MHz, $CDCl_3$)

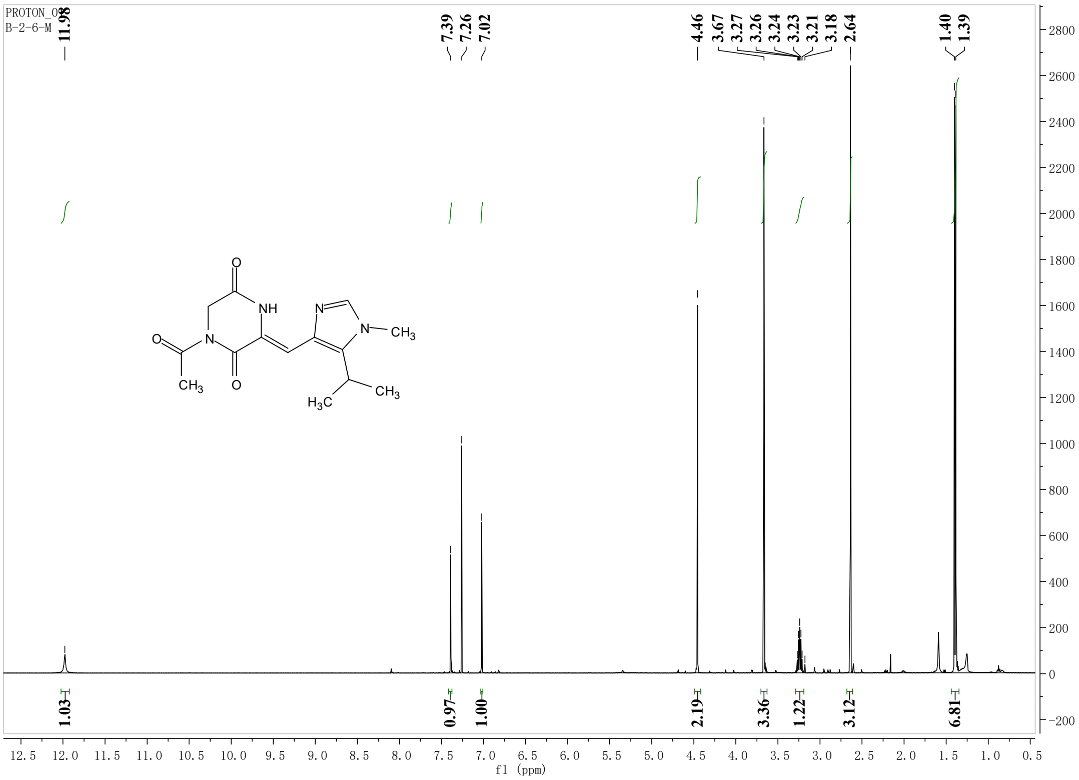

Figure S10. ^{1}H NMR spectrum for **13c** (500 MHz, $CDCl_3$)

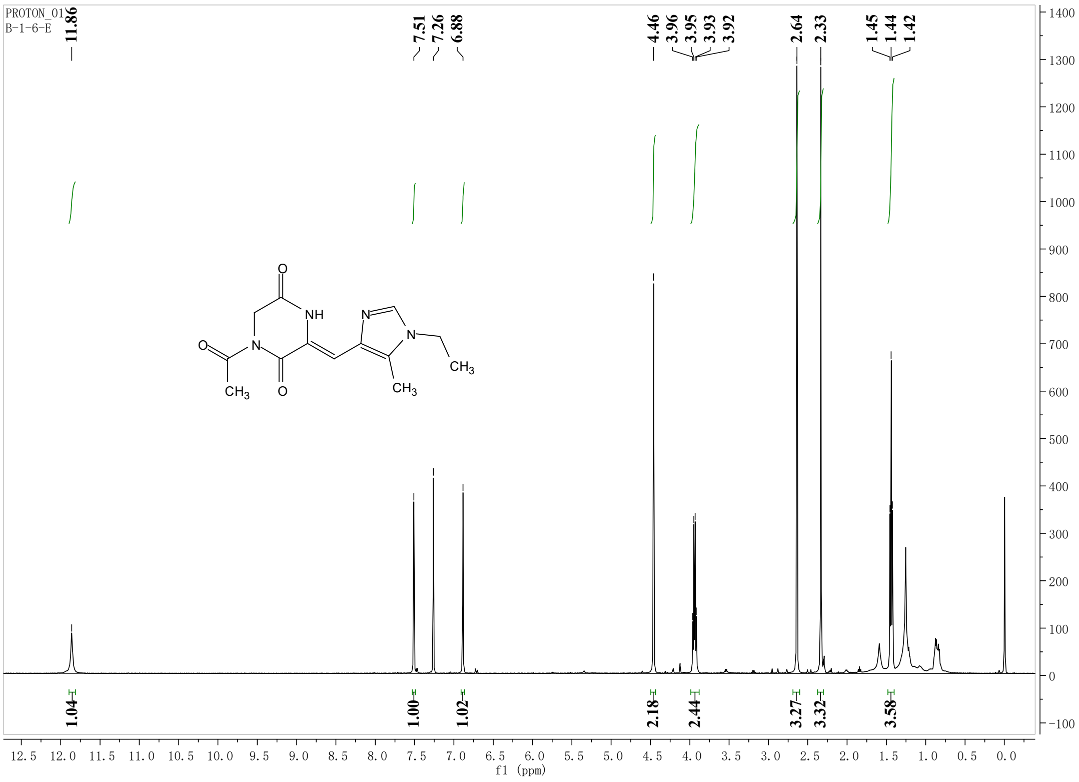

Figure S11. ^{1}H NMR spectrum for **13d** (500 MHz, $CDCl_3$)

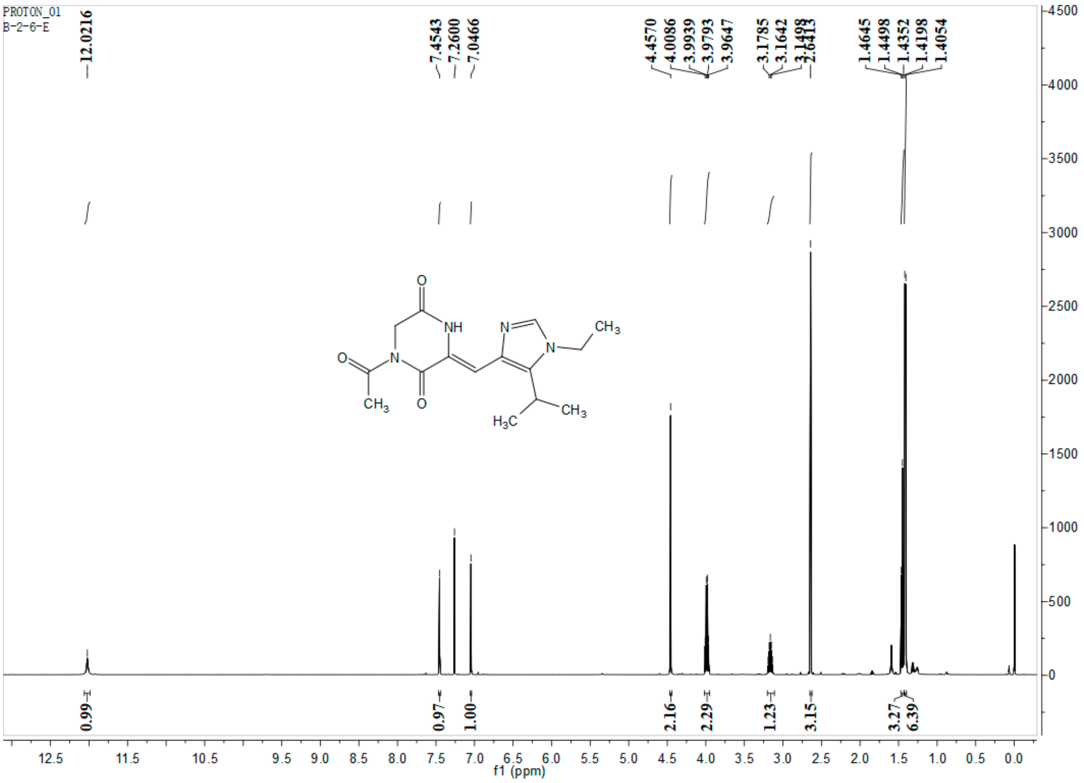

Figure S12. ^{13}C NMR spectrum for **13d** (125 MHz, $CDCl_3$)

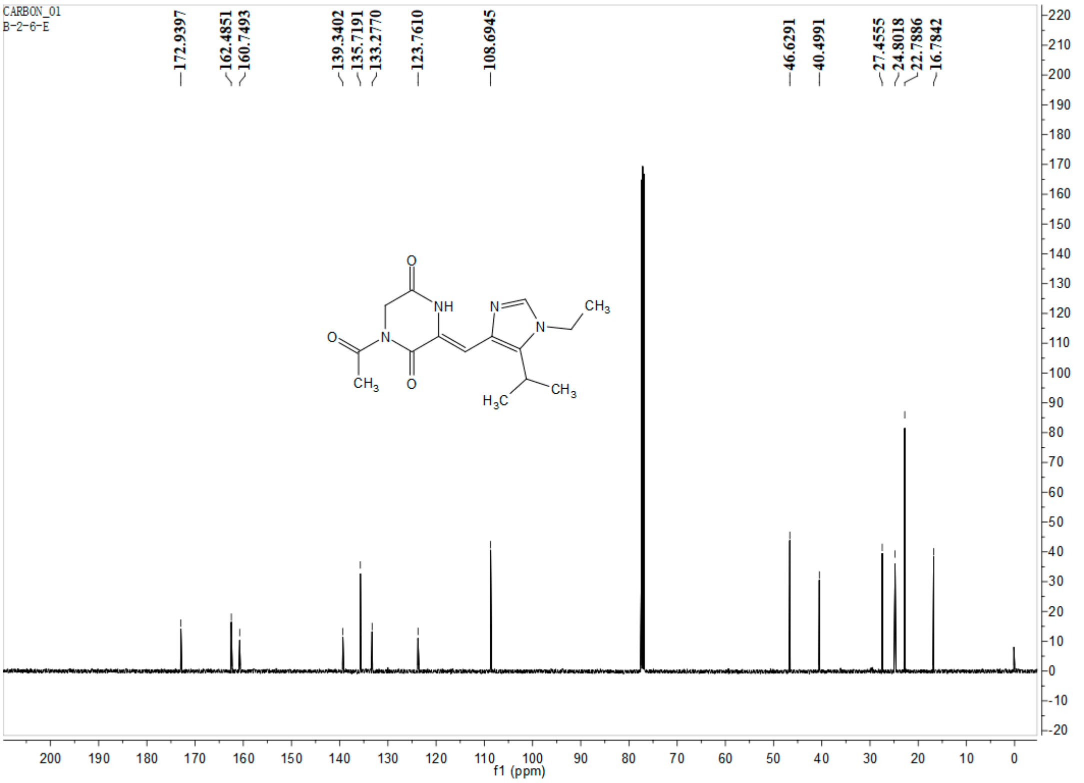

Figure S13. HMBC spectrum for **13d** (500 MHz, $CDCl_3$)

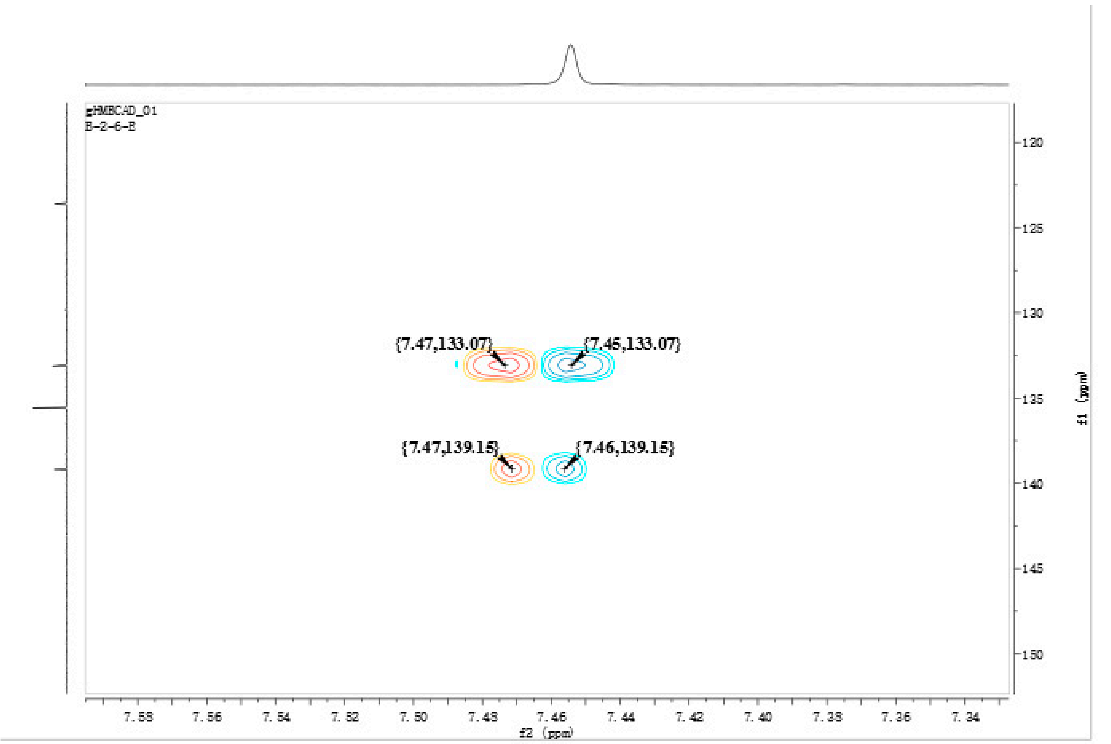

Figure S14. HMBC spectrum for **13d** (500 MHz, $CDCl_3$)

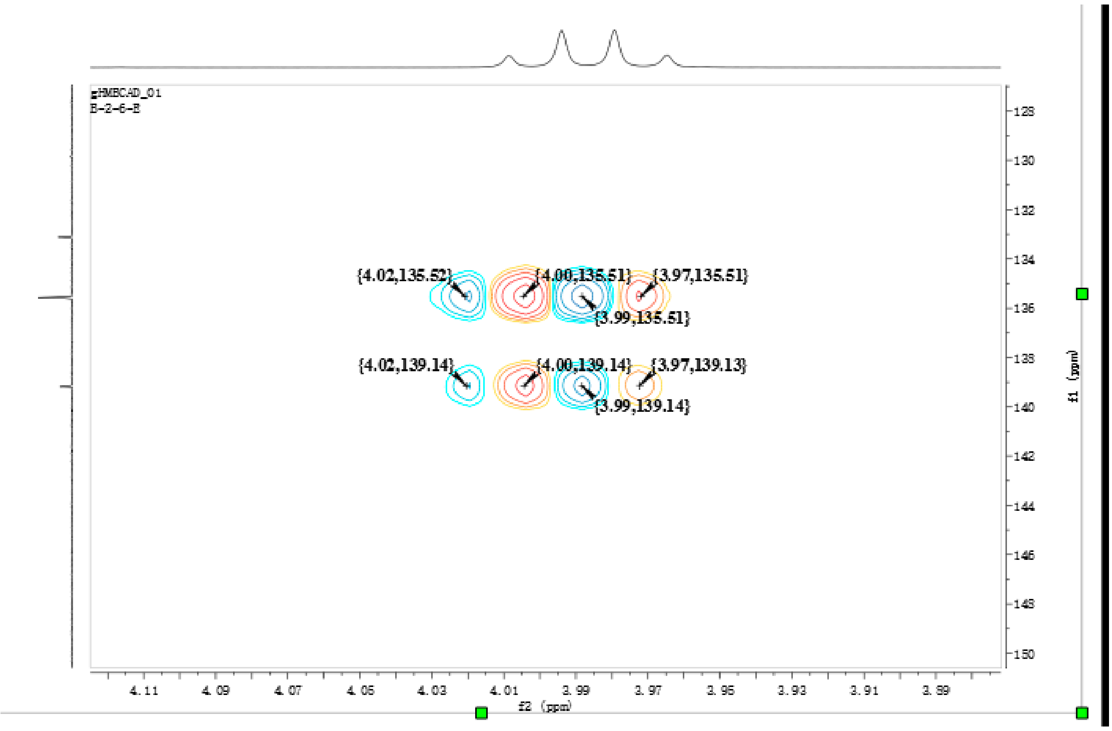

Figure S15. HMBC spectrum for **13d** (500 MHz, $CDCl_3$)

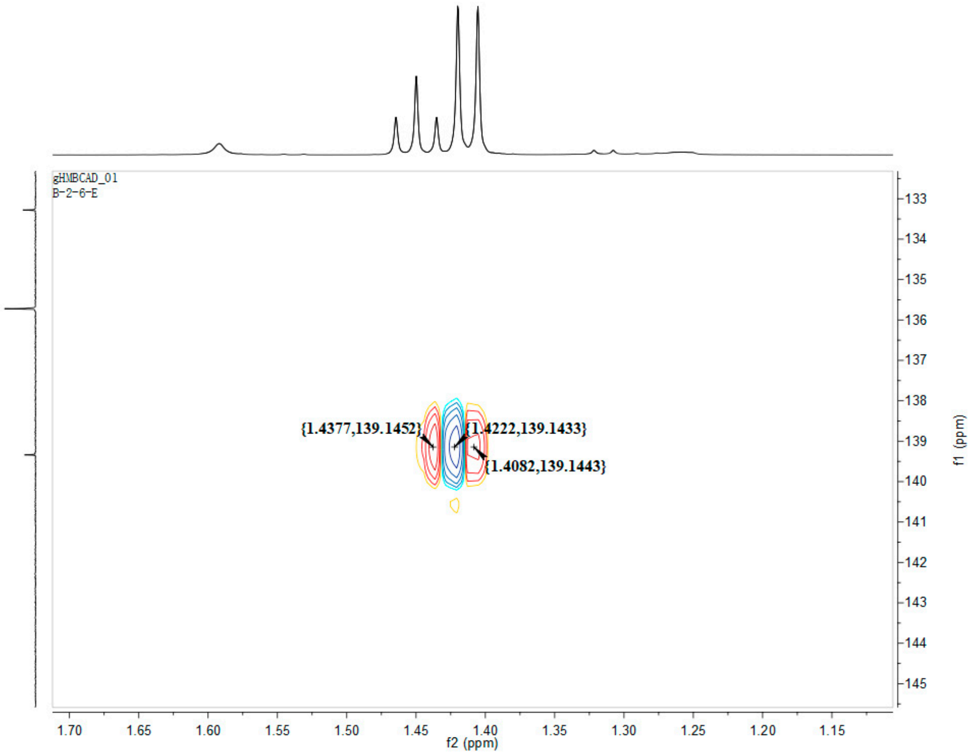

Figure S16. ^{1}H NMR spectrum for **13e** (500 MHz, $CDCl_3$)

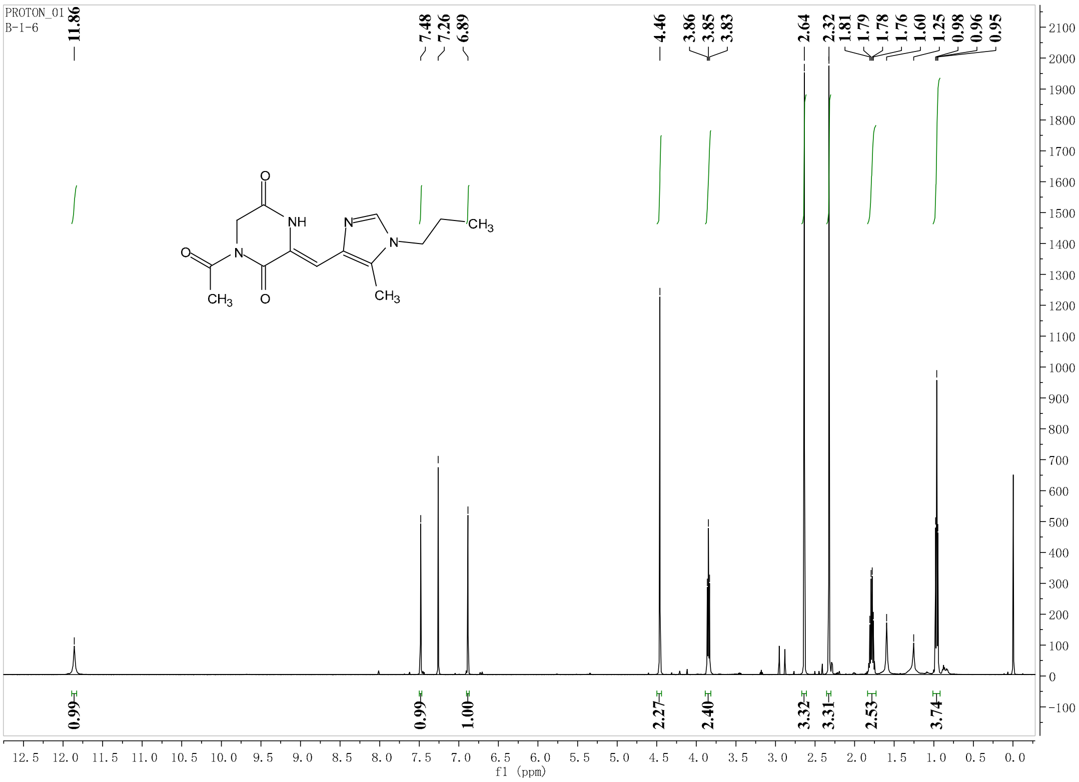

Figure S17. ^{1}H NMR spectrum for **13f** (500 MHz, $CDCl_3$)

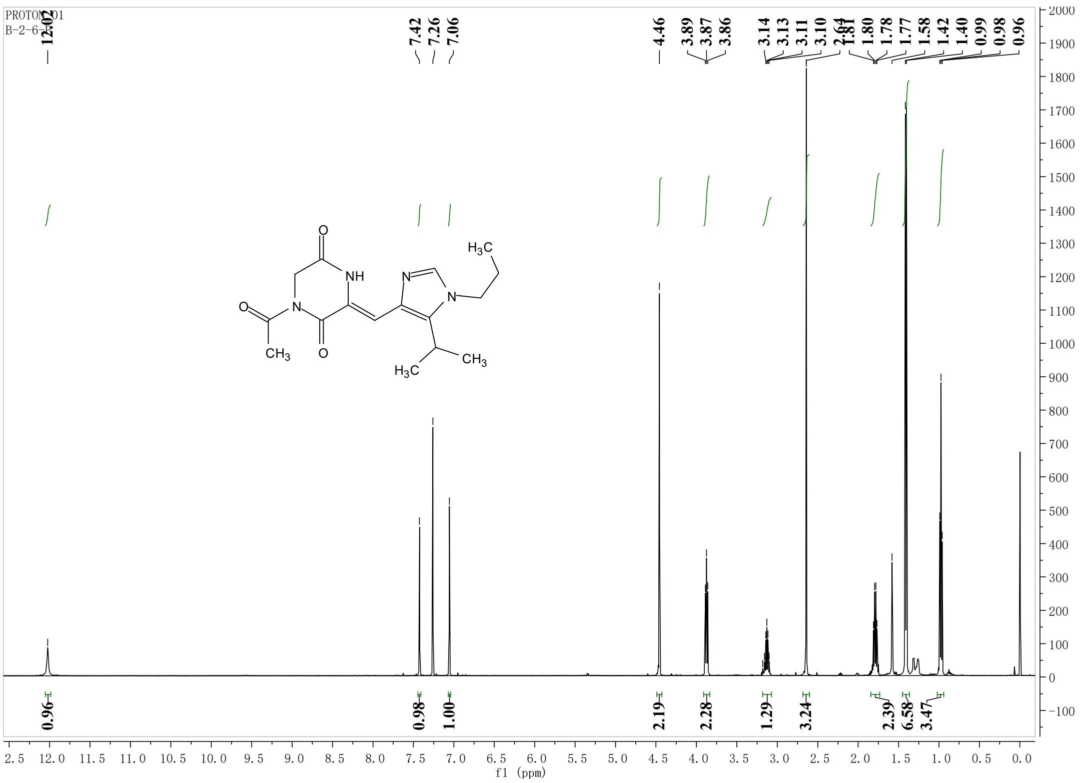

Figure S18. ^{1}H NMR spectrum for **13g** (400 MHz, $CDCl_3$)

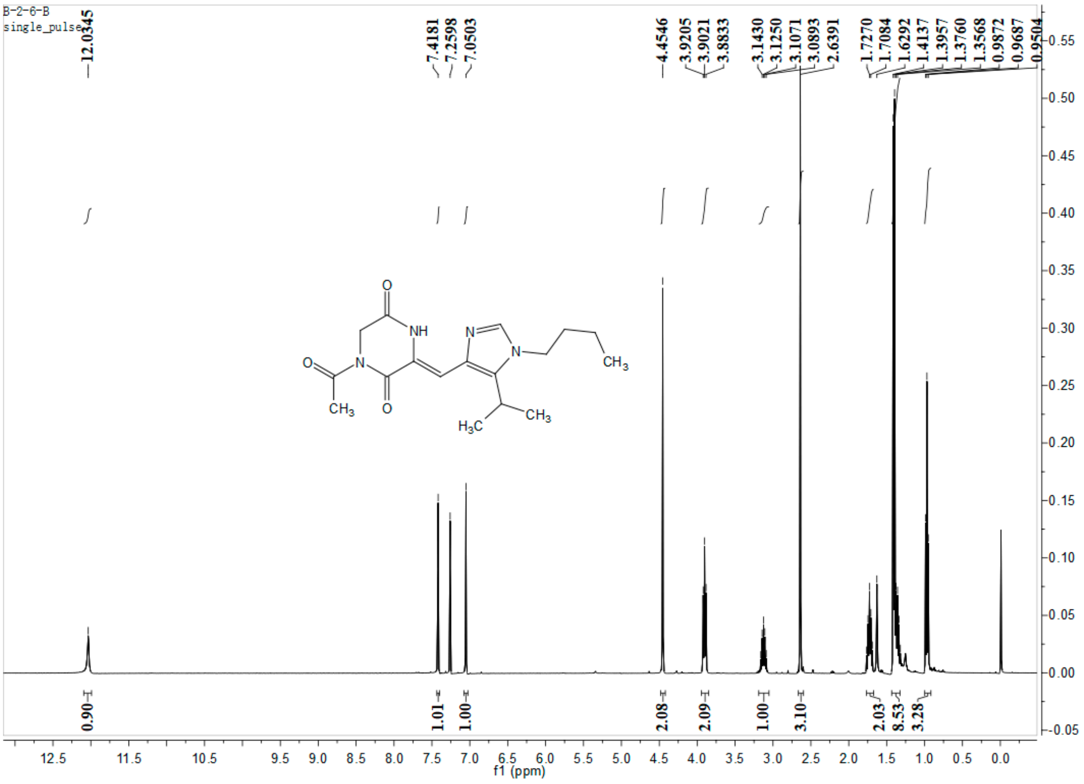

Figure S19. ^{1}H NMR spectrum for **13h** (500 MHz, $CDCl_3$)

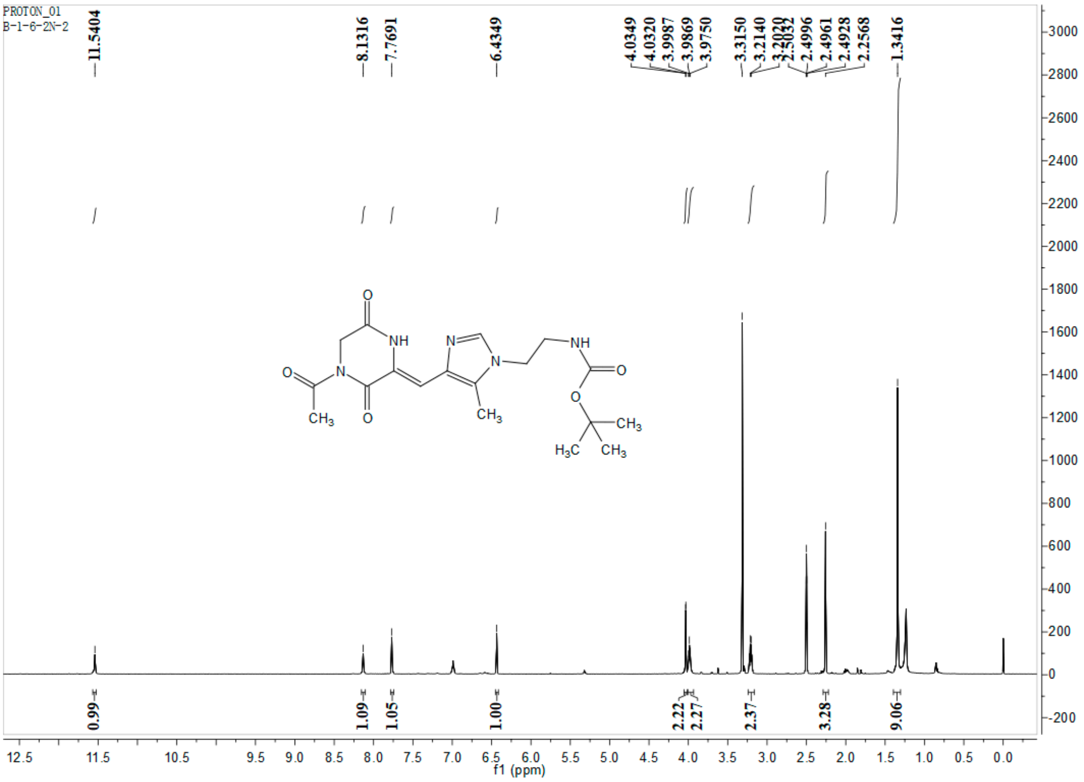

Figure S20. ^{1}H NMR spectrum for **13j** (500 MHz, DMSO-d_6)

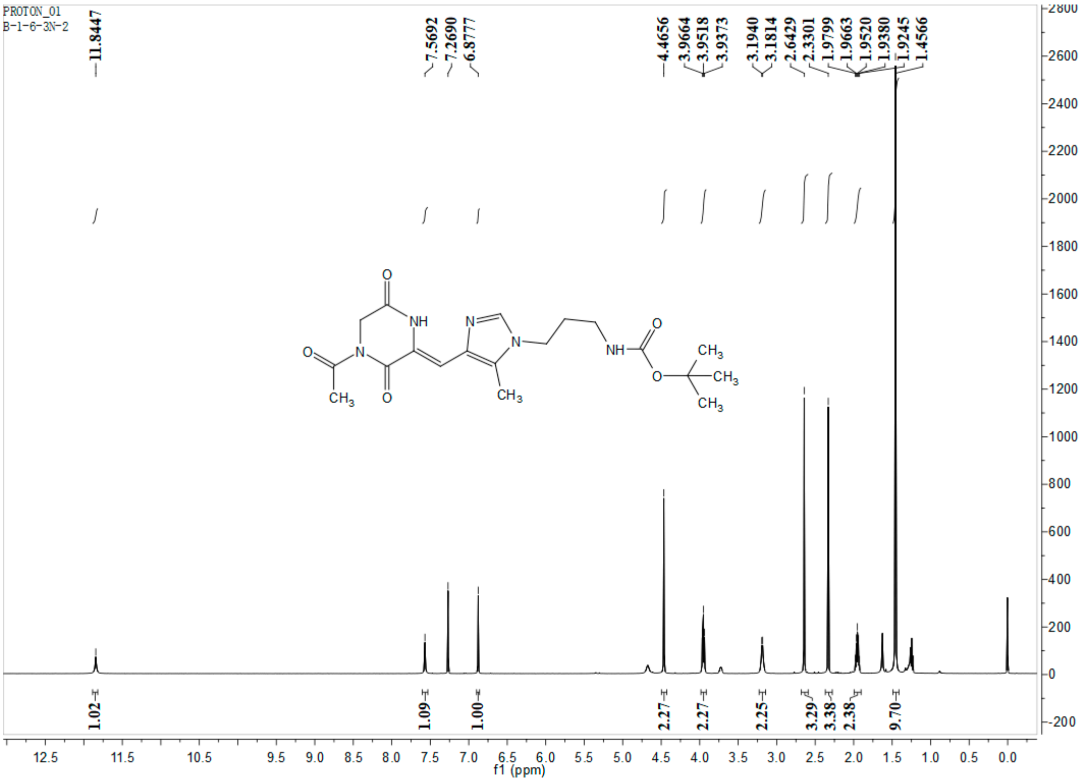

Figure S21. ^{1}H NMR spectrum for **13k** ((500 MHz, $CDCl_3$))

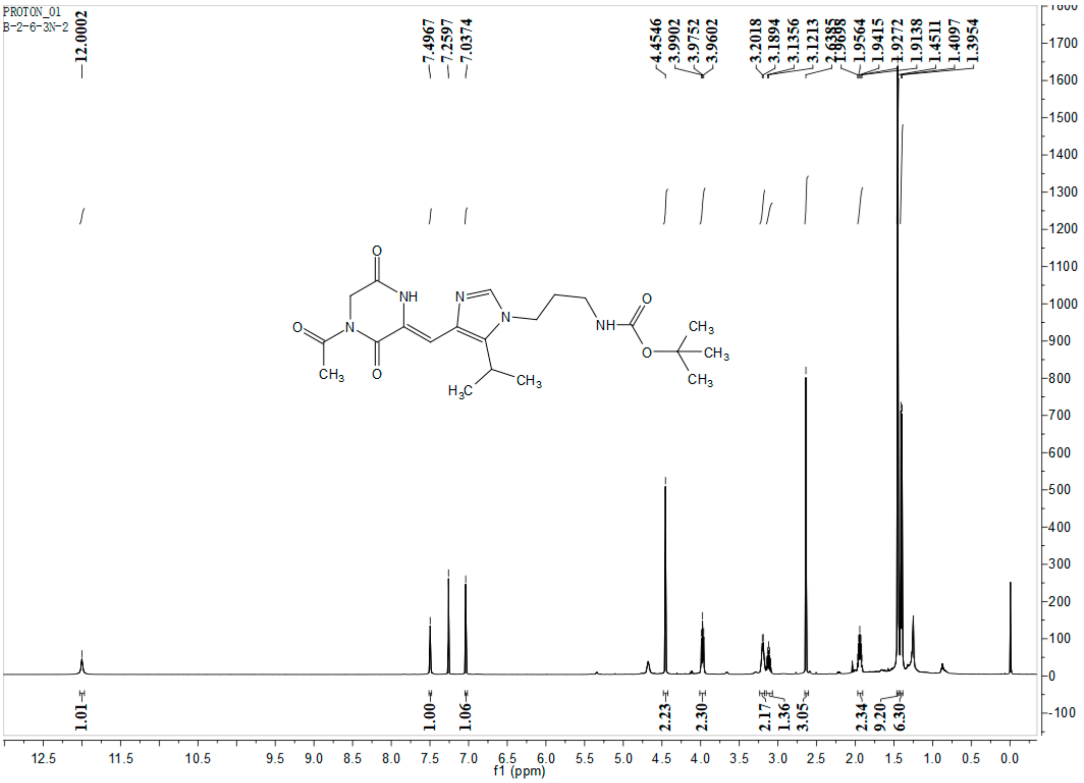

Figure S22. ^{1}H NMR spectrum for **13l** (500 MHz, $CDCl_3$)

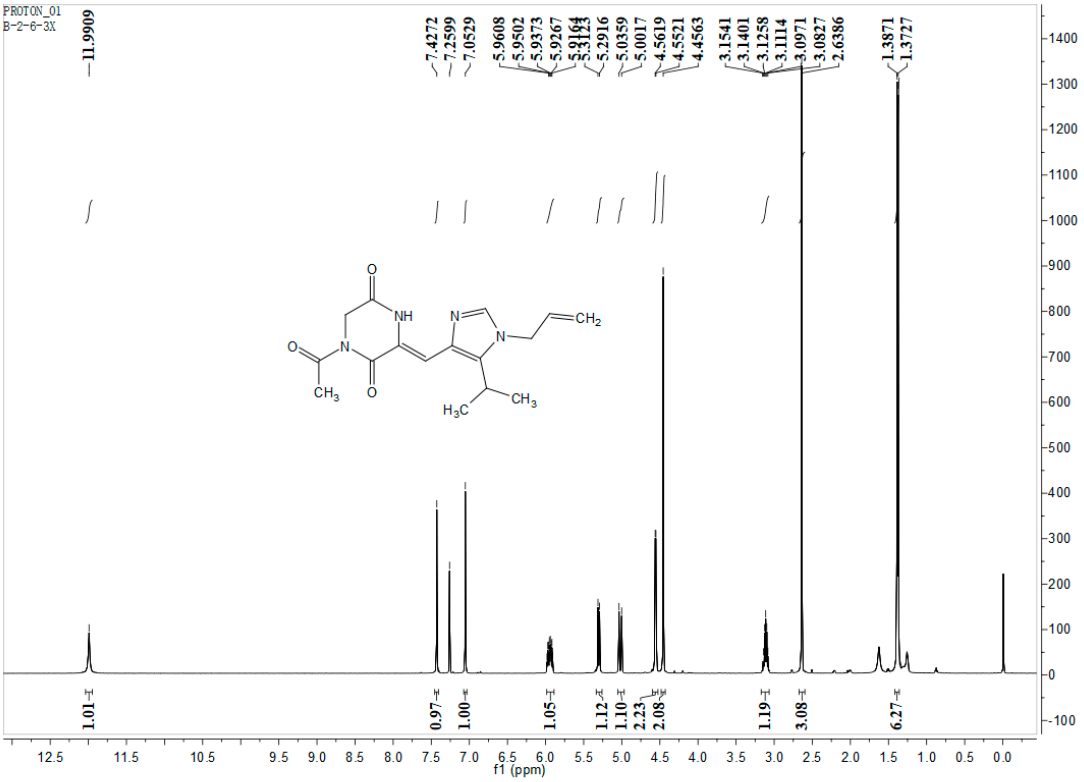

Figure S23. ^{1}H NMR spectrum for **13m** (500 MHz, $CDCl_3$)

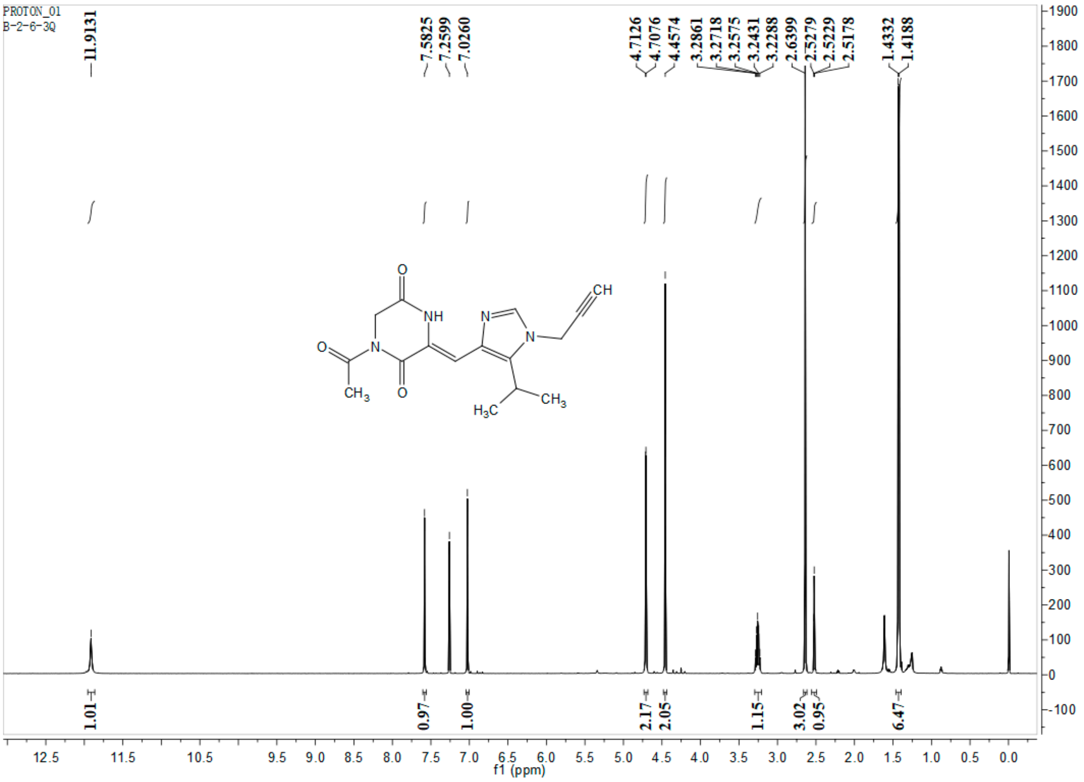

Figure S24. ^{1}H NMR spectrum for **15a** (500 MHz, DMSO-d_6)

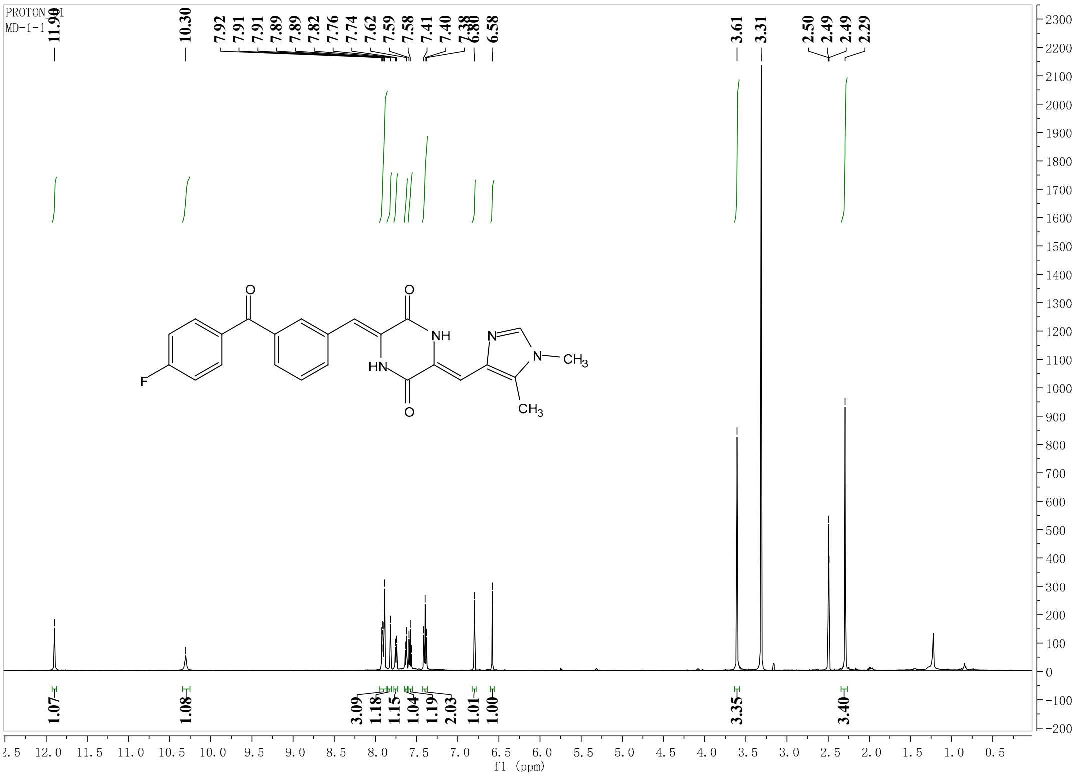

Figure S25. ^{13}C NMR spectrum for **15a** (125 MHz, DMSO-d_6)

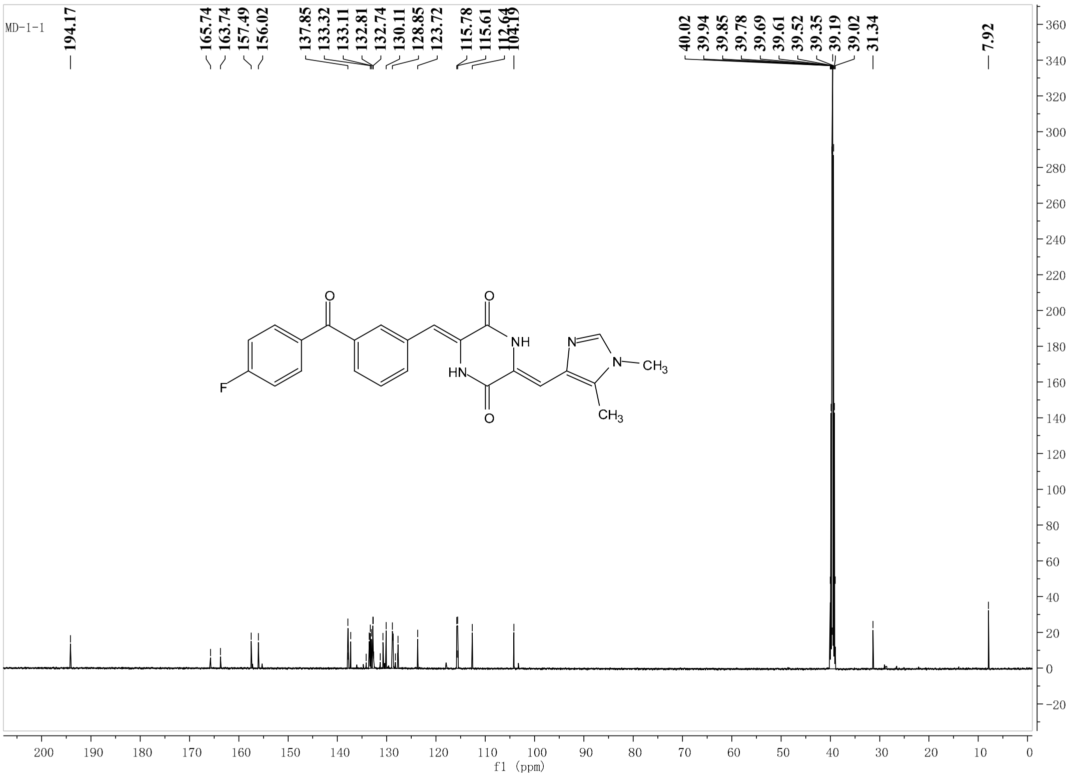

Figure S26. HRMS spectrum for **15a** (MeOH)

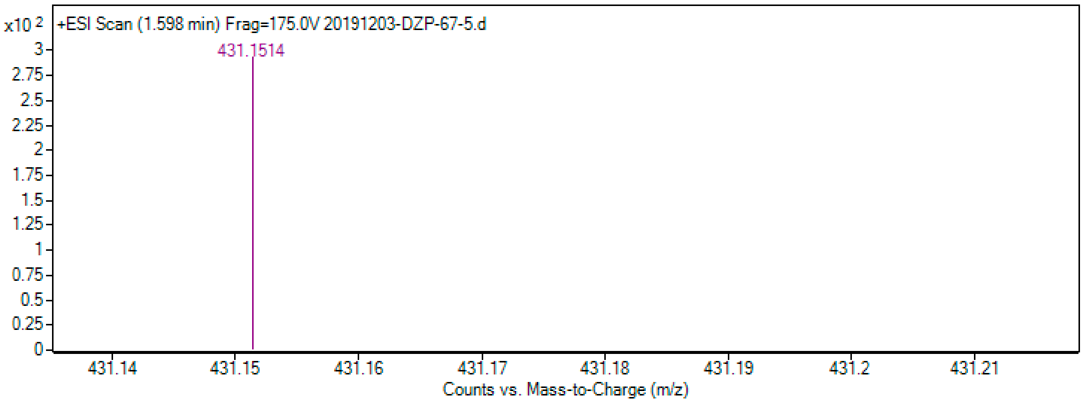

Figure S27. ^{1}H NMR spectrum for **15b** (500 MHz, DMSO-*d*$_6$)

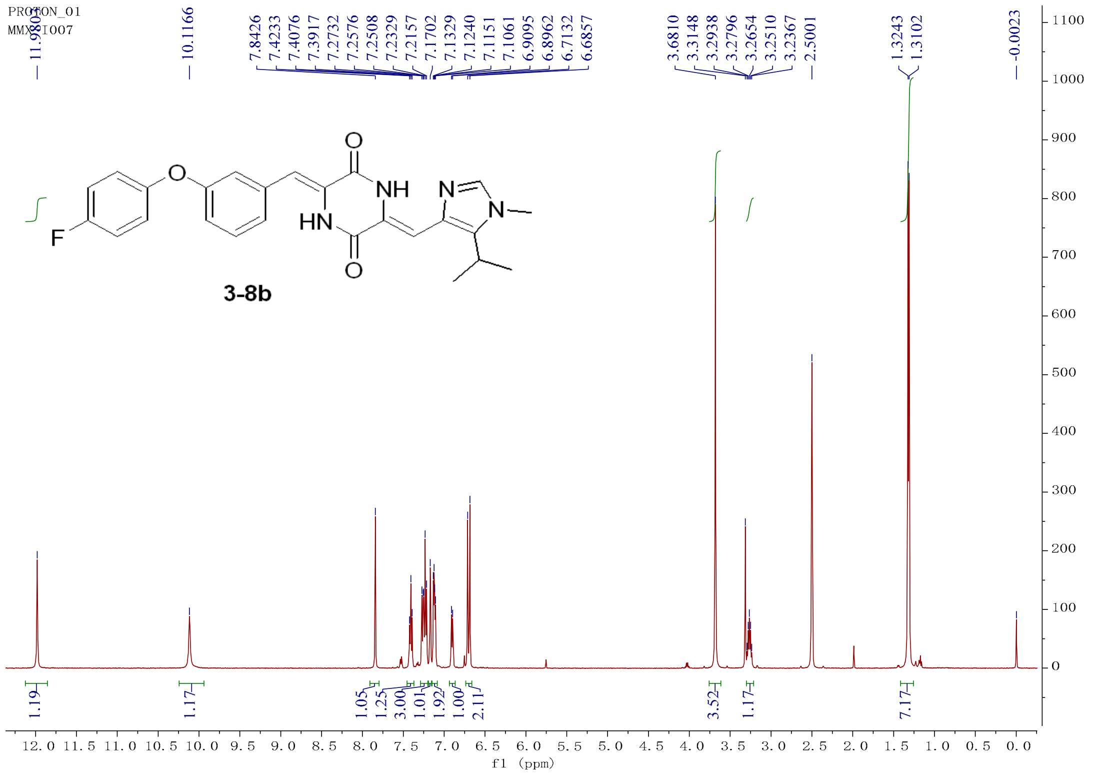

Figure S28. ^{13}C NMR spectrum for **15b** (125 MHz, DMSO-*d*$_6$)

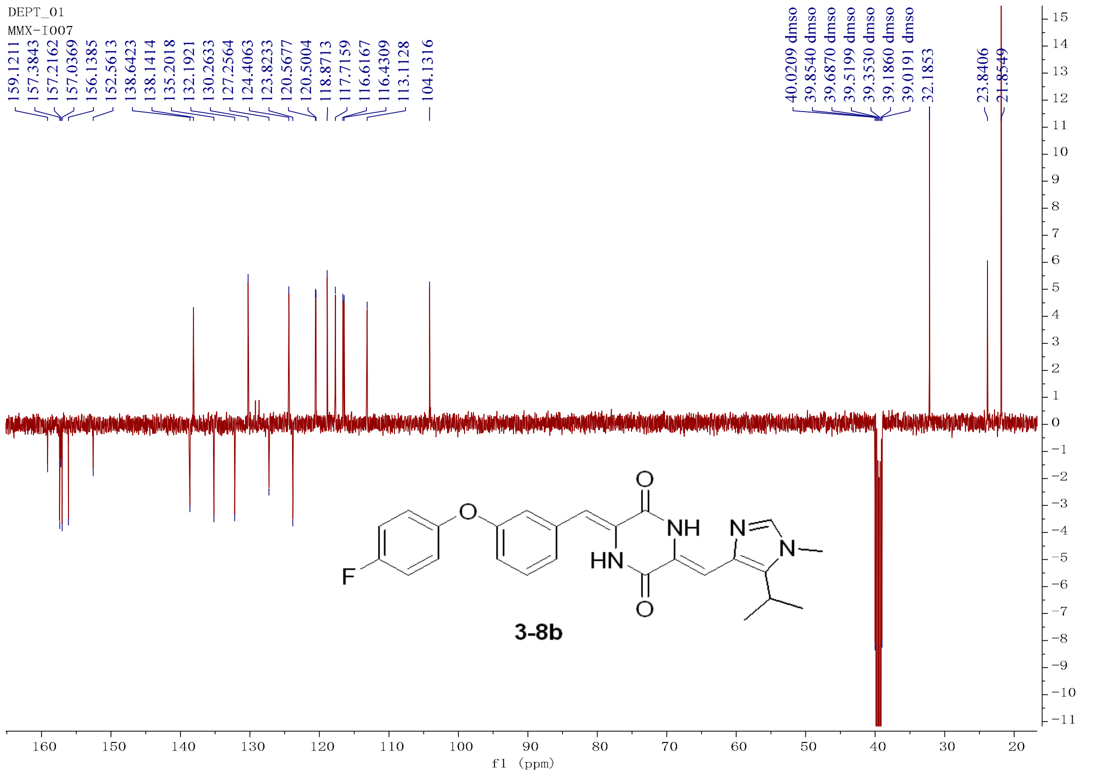

Figure S29. HRMS spectrum for **15b** (MeOH)

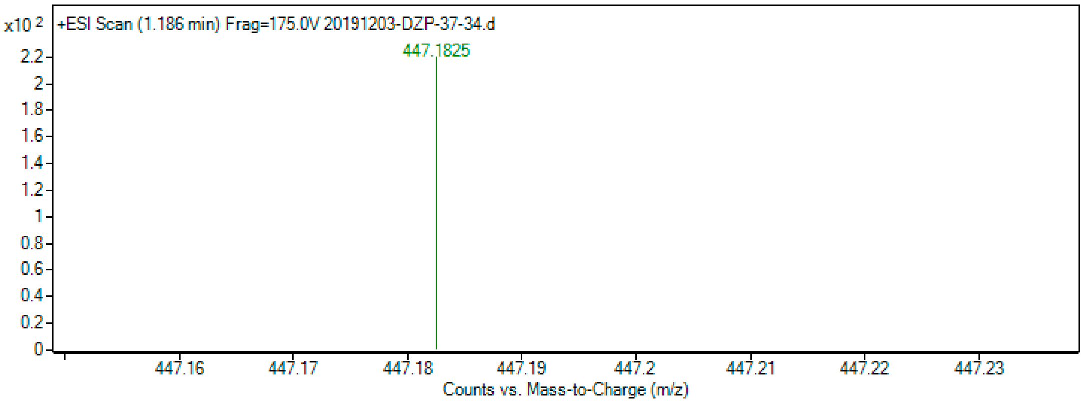

Figure S30. ^{1}H NMR spectrum for **15c** (500 MHz, DMSO-d_6)

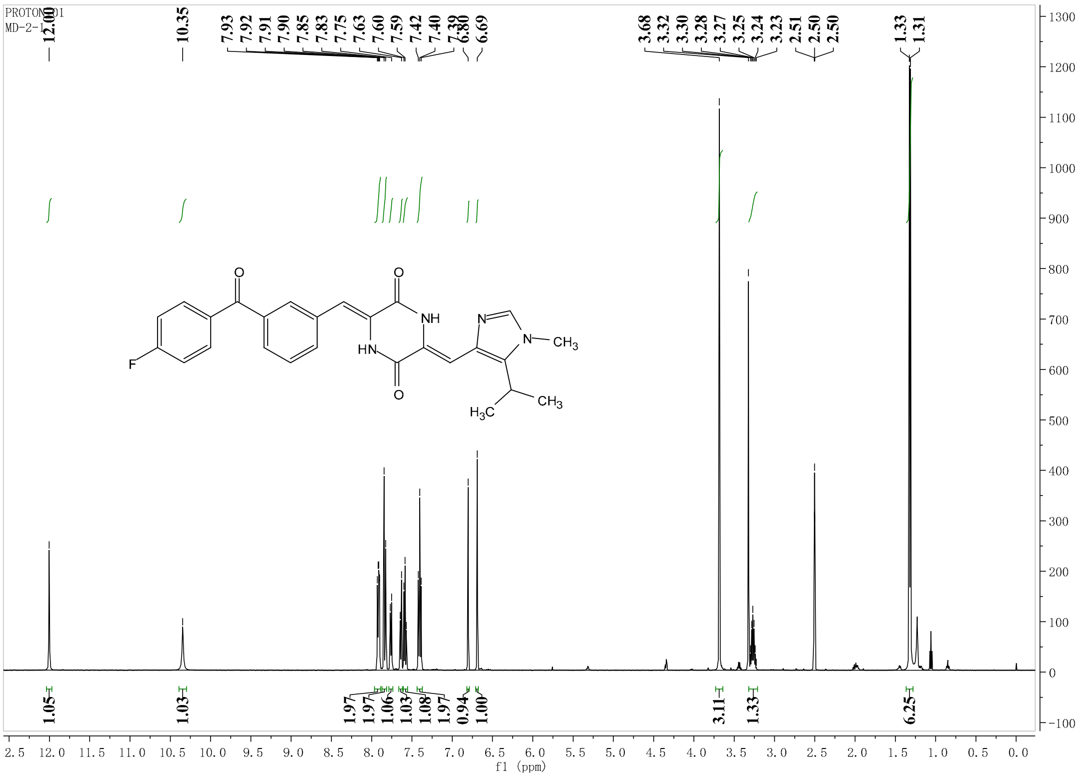

Figure S31. ^{13}C NMR spectrum for **15c** (125 MHz, DMSO-d_6)

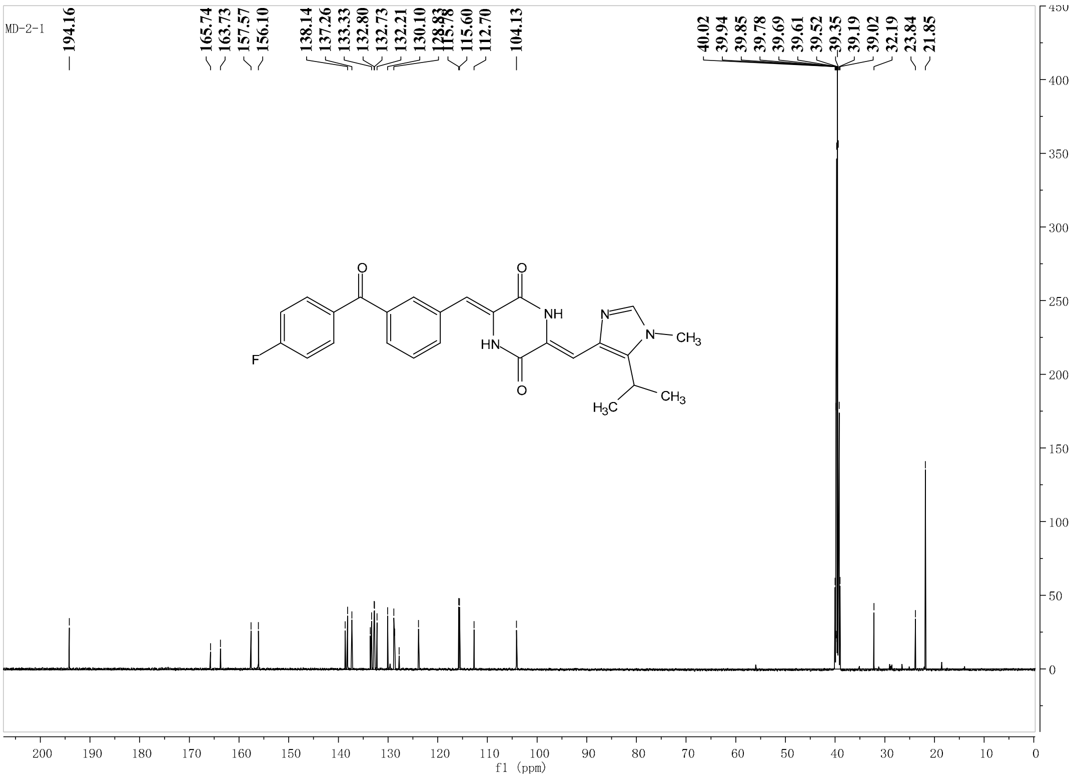

Figure S32. HRMS spectrum for **15c** (MeOH)

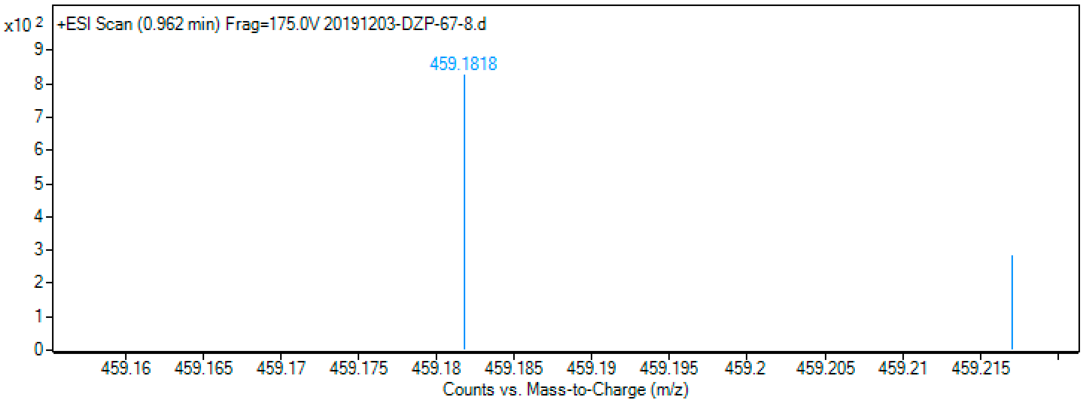

Figure S33. ^{1}H NMR spectrum for **15d** (500 MHz, DMSO-d_6)

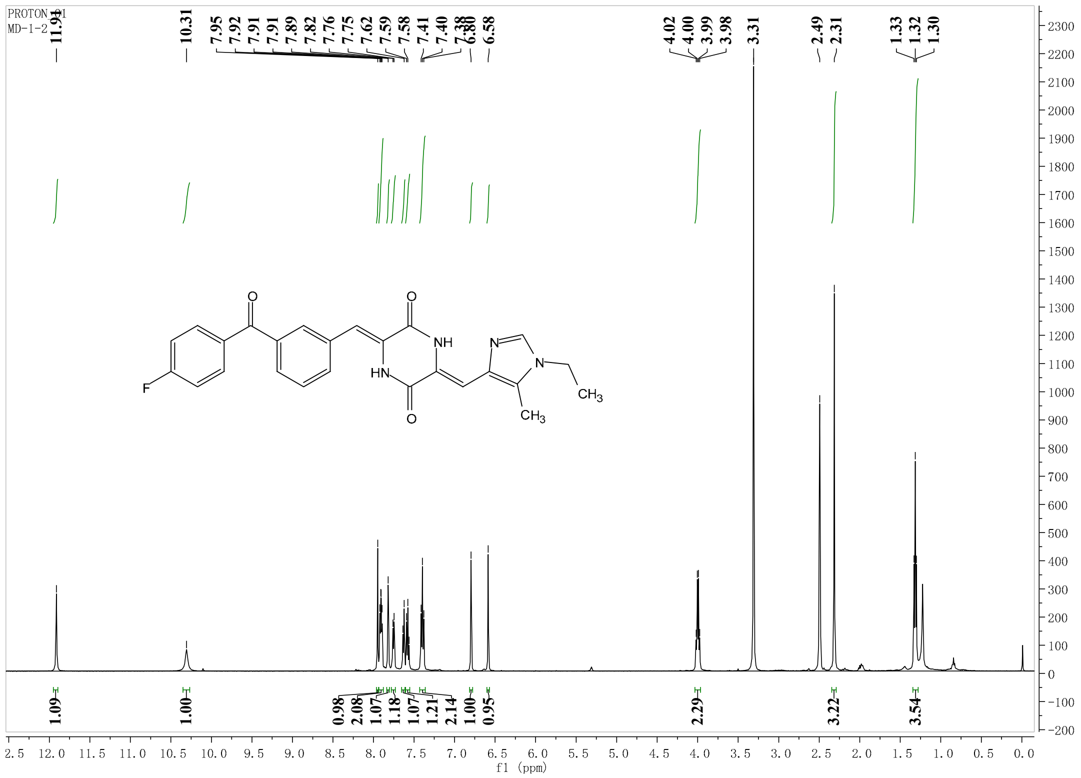

Figure S34. ^{13}C NMR spectrum for **15d** (125 MHz, DMSO-d_6)

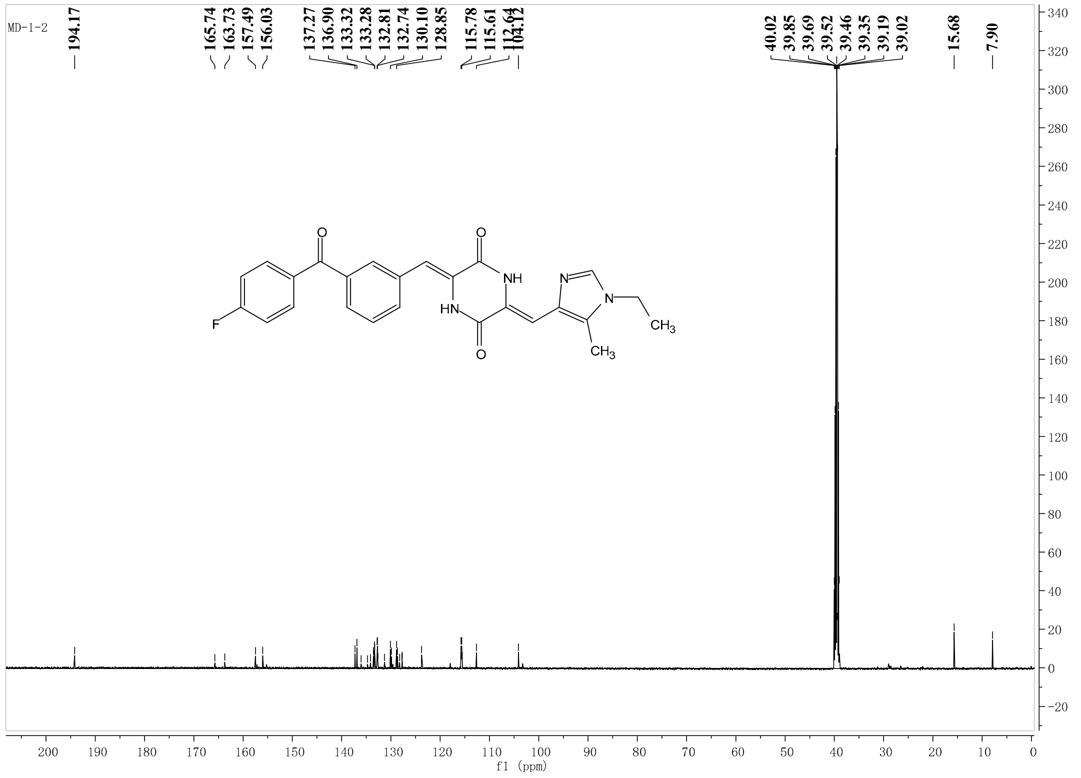

Figure S35. HRMS spectrum for **15d** (MeOH)

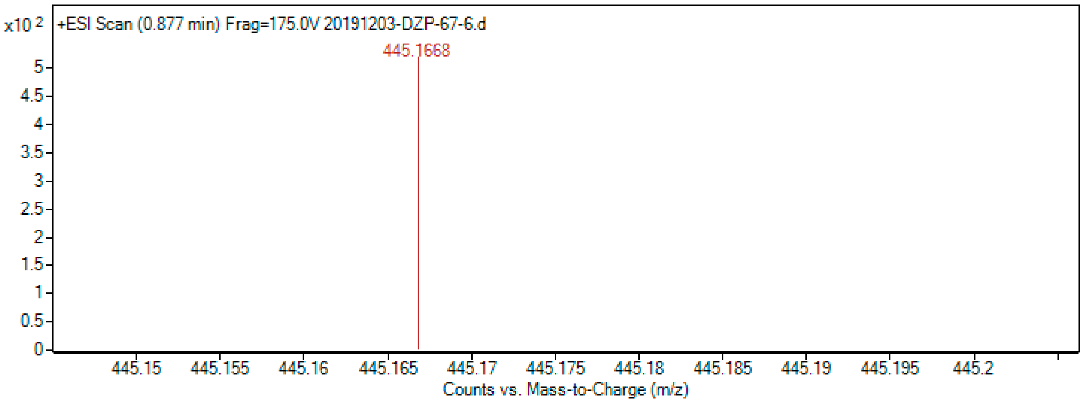

Figure S36. ^{1}H NMR spectrum for **15e** (500 MHz, DMSO-d_6)

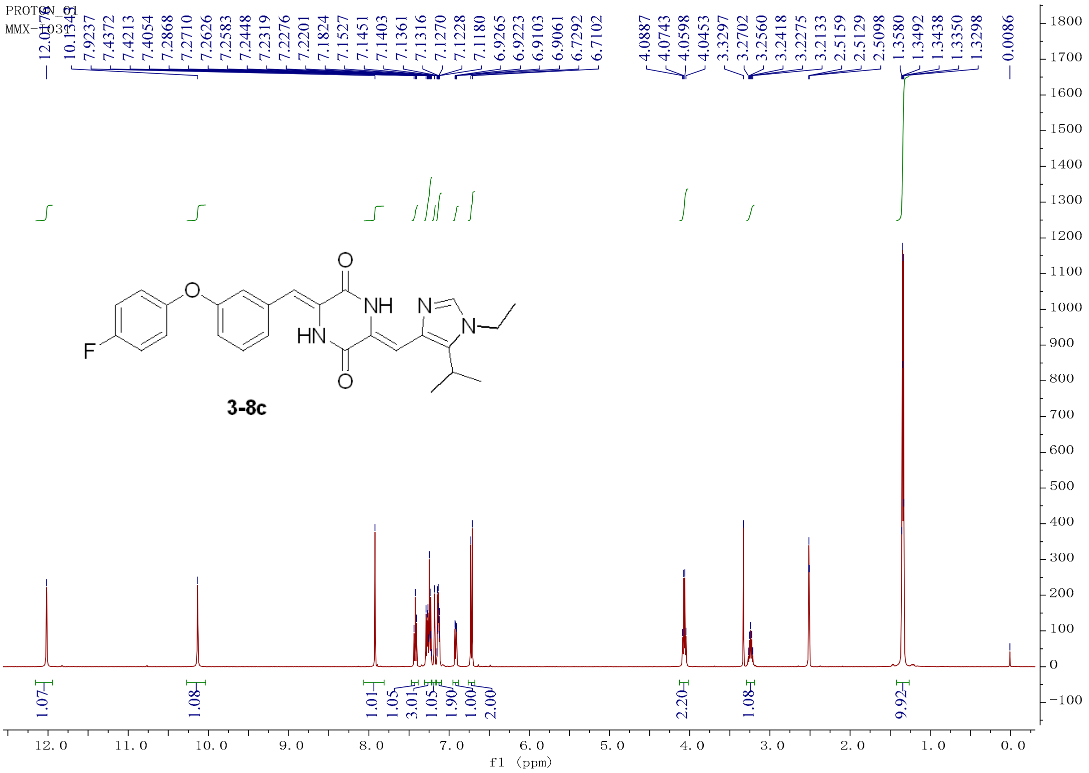

Figure S37. ^{13}C NMR spectrum for **15e** (125 MHz, DMSO-d_6)

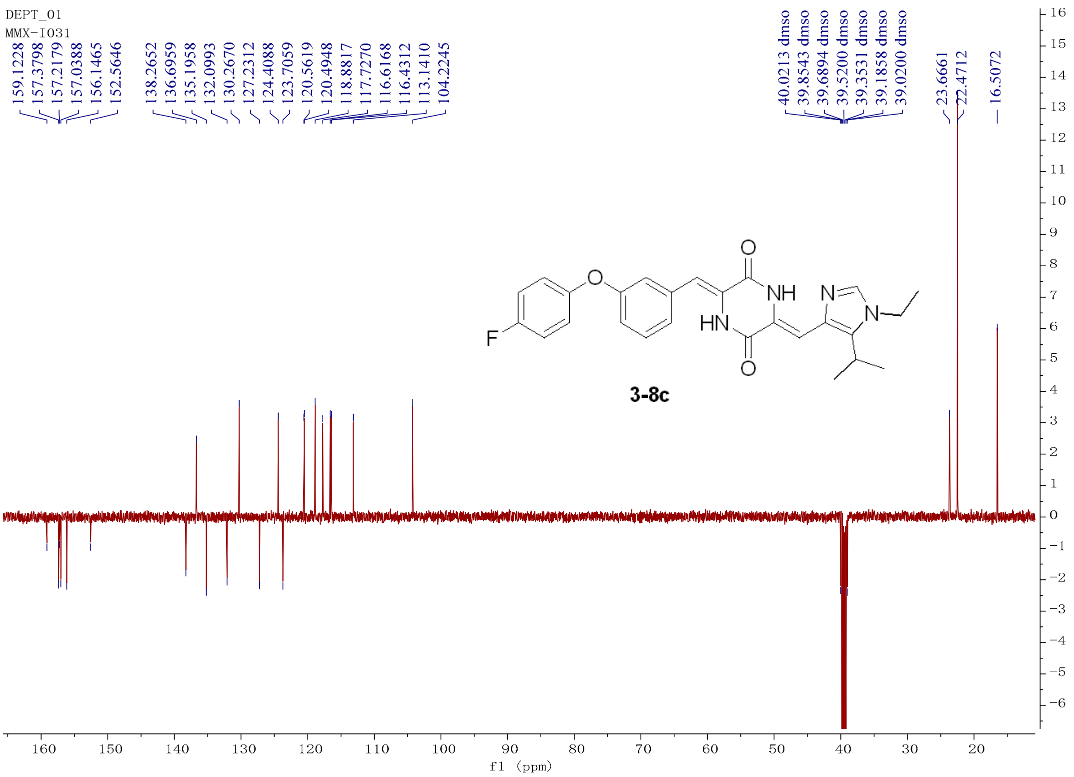

Figure S38. HRMS spectrum for **15e**(MeOH)

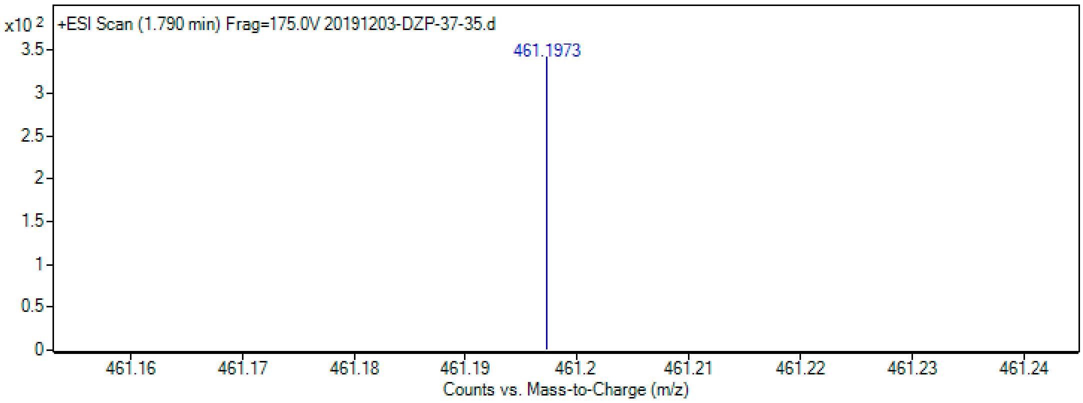

Figure S39. ^{1}H NMR spectrum for **15f** (500 MHz, DMSO-d_6)

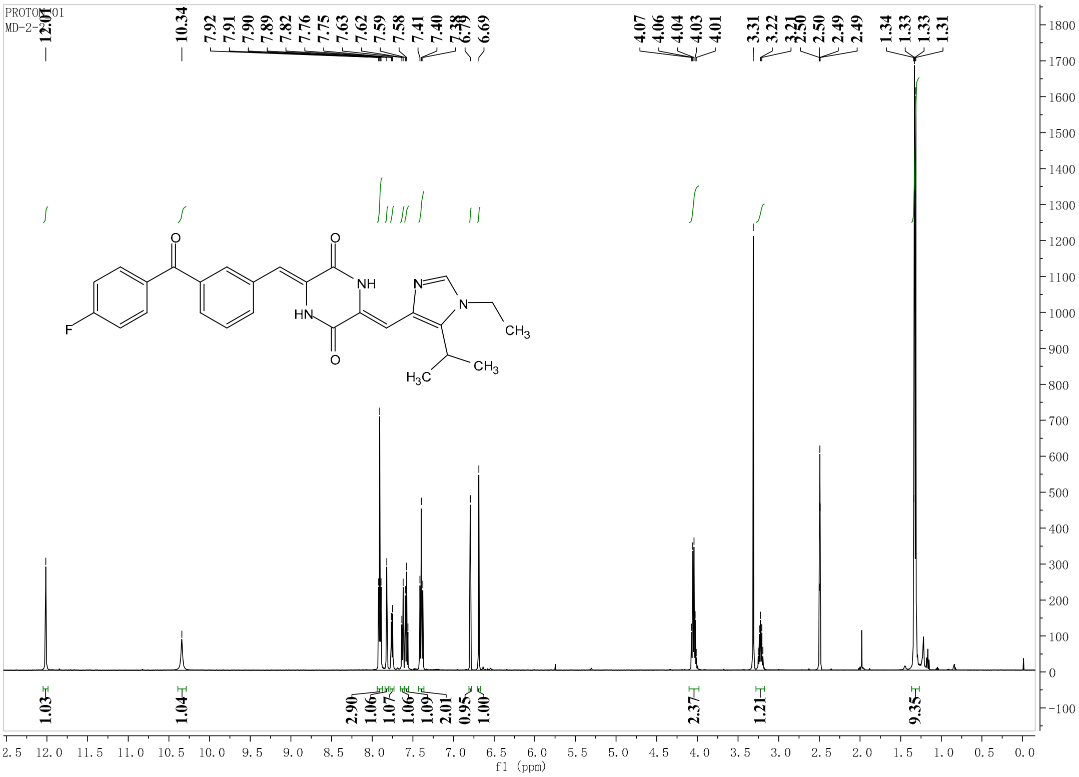

Figure S40. ^{13}C NMR spectrum for **15f** (125 MHz, DMSO-d_6)

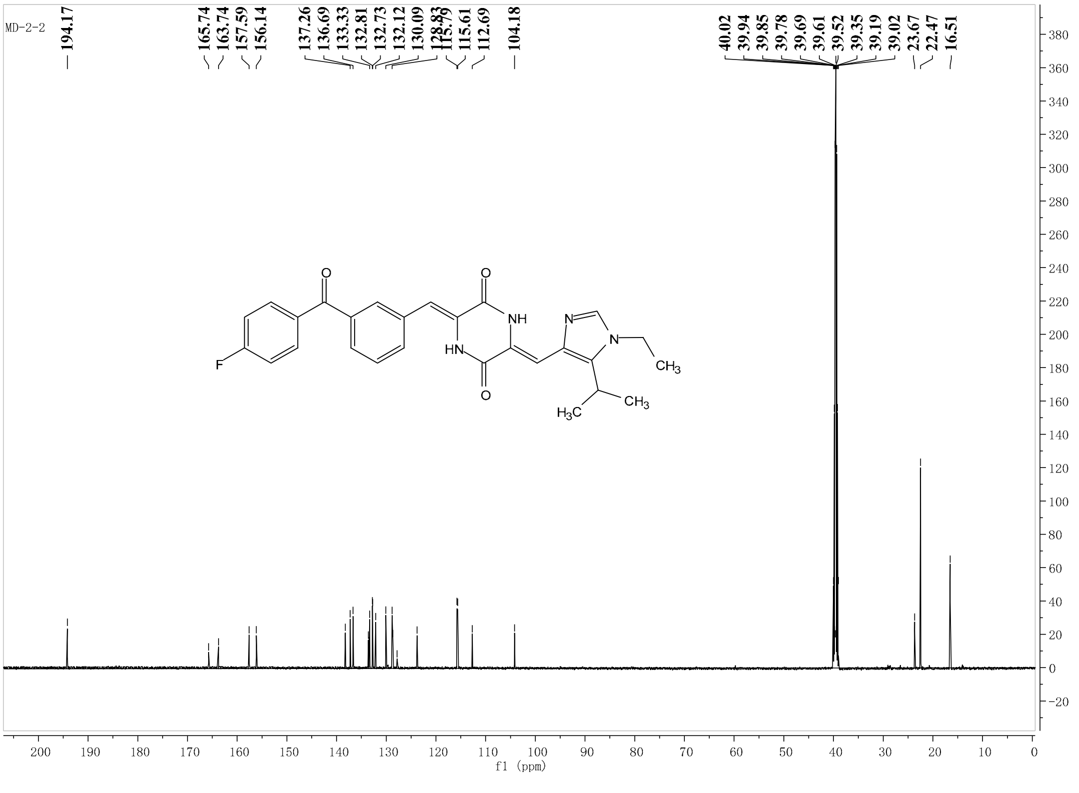

Figure S41. HRMS spectrum for **15f** (MeOH)

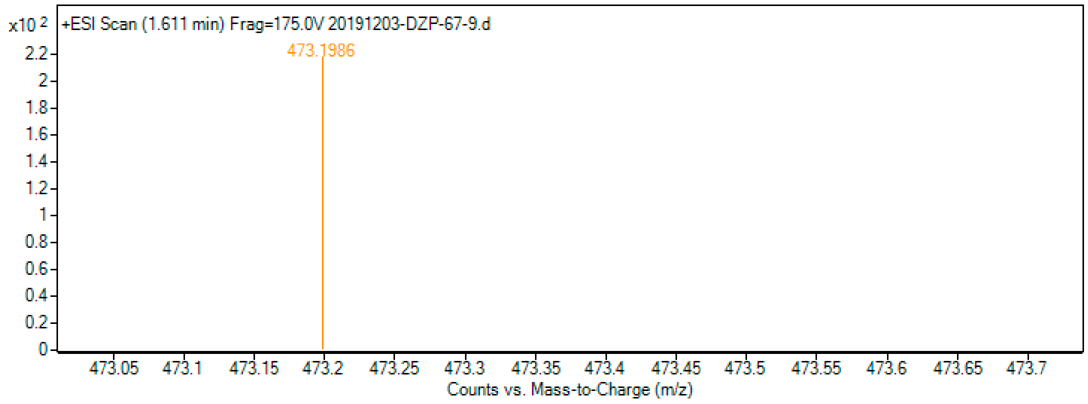

Figure S42. ^{1}H NMR spectrum for **15g** (500 MHz, DMSO-d_6)

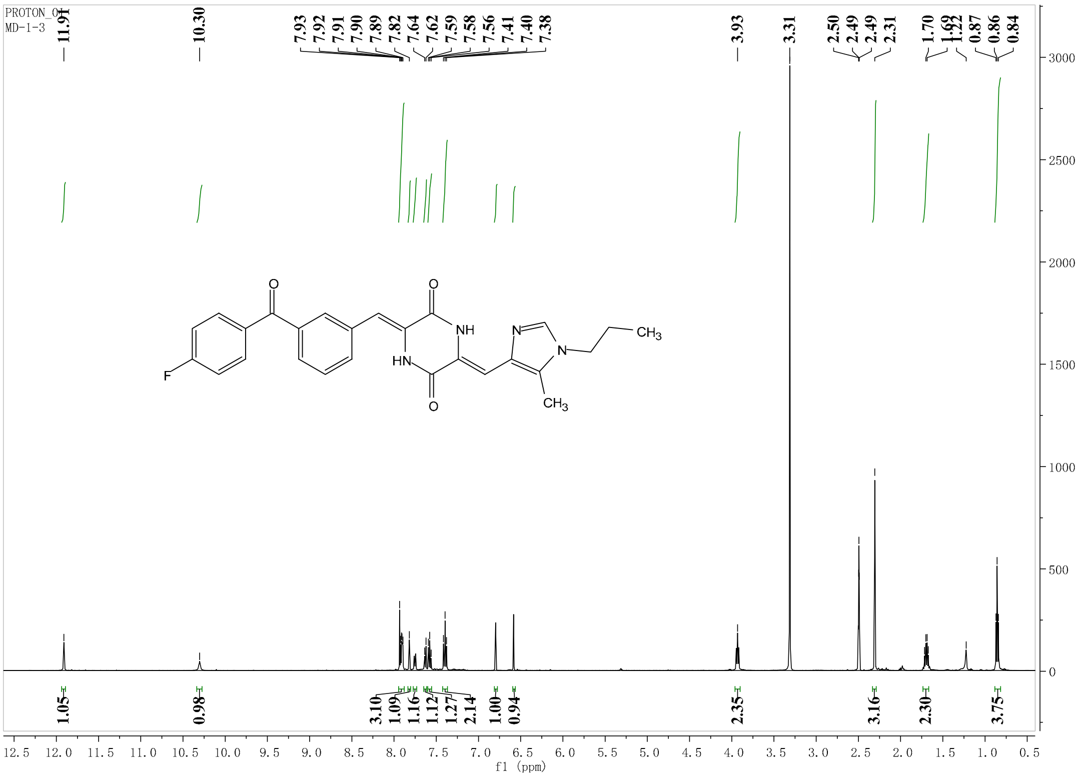

Figure S43. ^{13}C NMR spectrum for **15g** (125 MHz, DMSO-d_6)

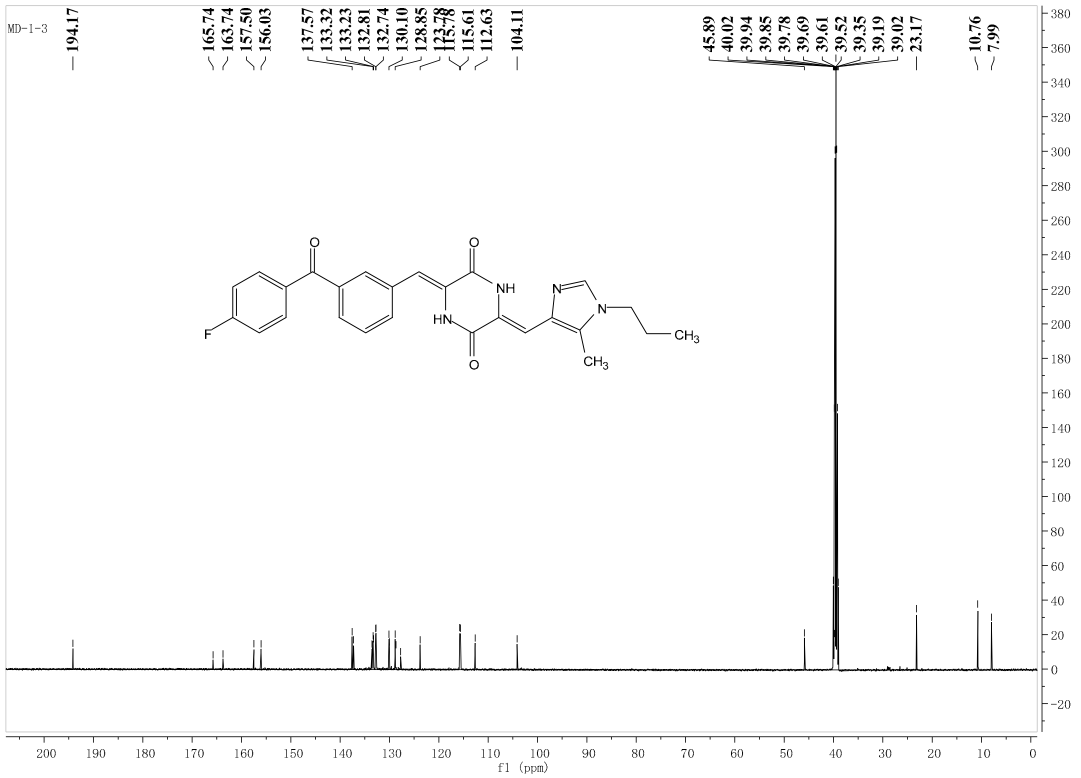

Figure S44. HRMS spectrum for **15g** (MeOH)

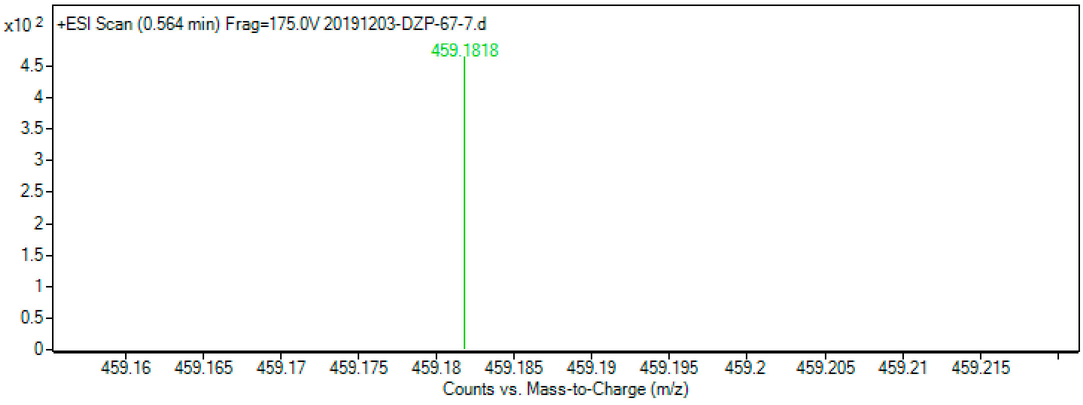

Figure S45. ^{1}H NMR spectrum for **15h** (500 MHz, DMSO-d_6)

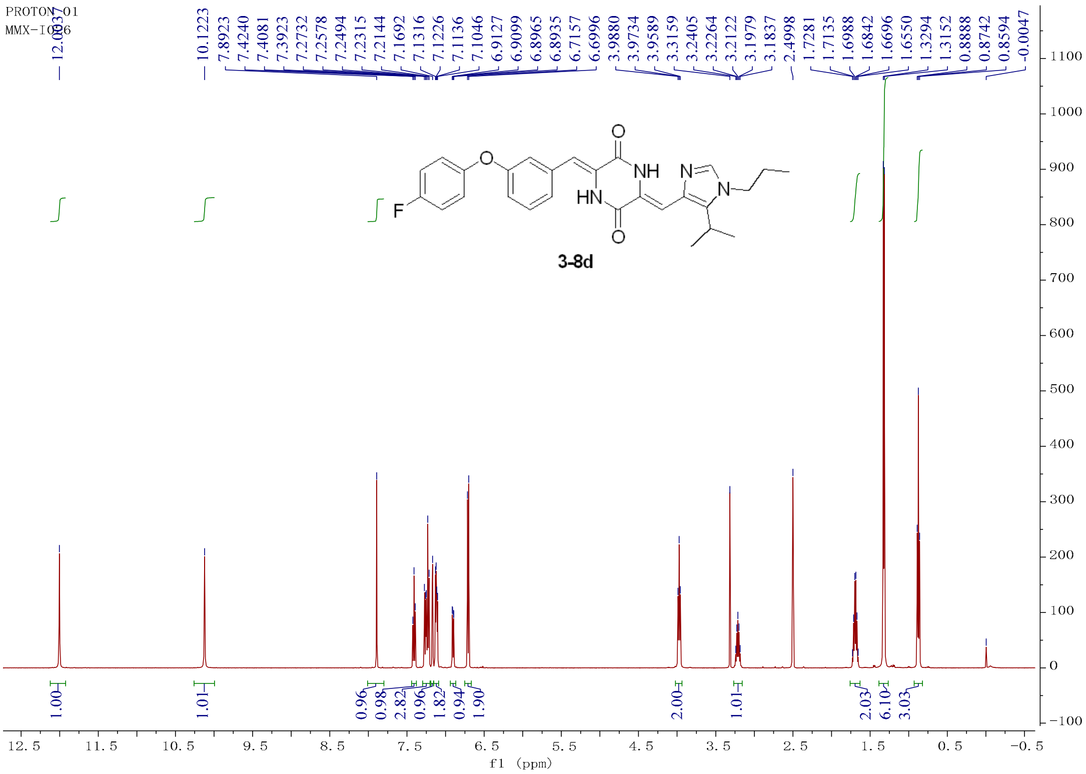

Figure S46. ^{13}C NMR spectrum for **15h** (125 MHz, DMSO-d_6)

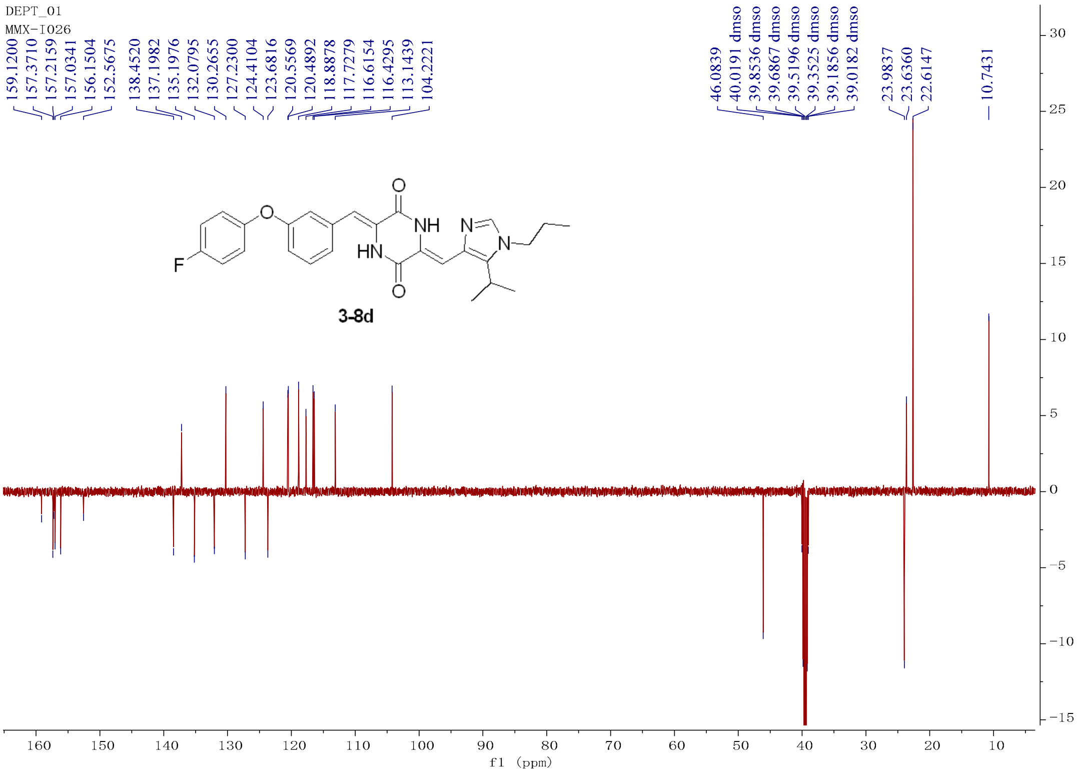

Figure S47. HRMS spectrum for **15h** (MeOH)

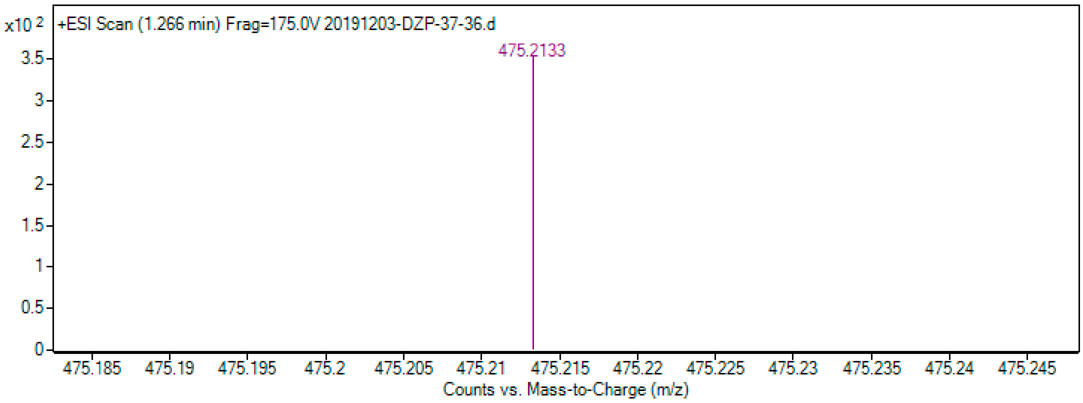

Figure S48. ^{1}H NMR spectrum for **15i** (500 MHz, DMSO-d_6)

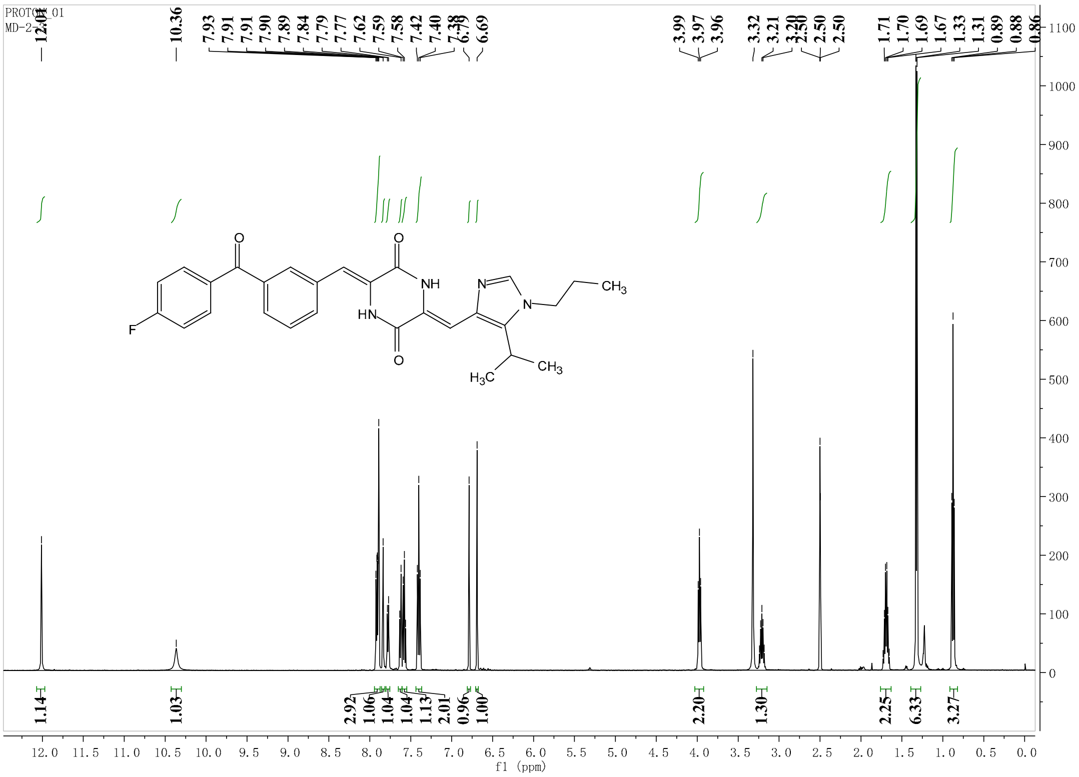

Figure S49. ^{13}C NMR spectrum for **15i** (125 MHz, DMSO-d_6)

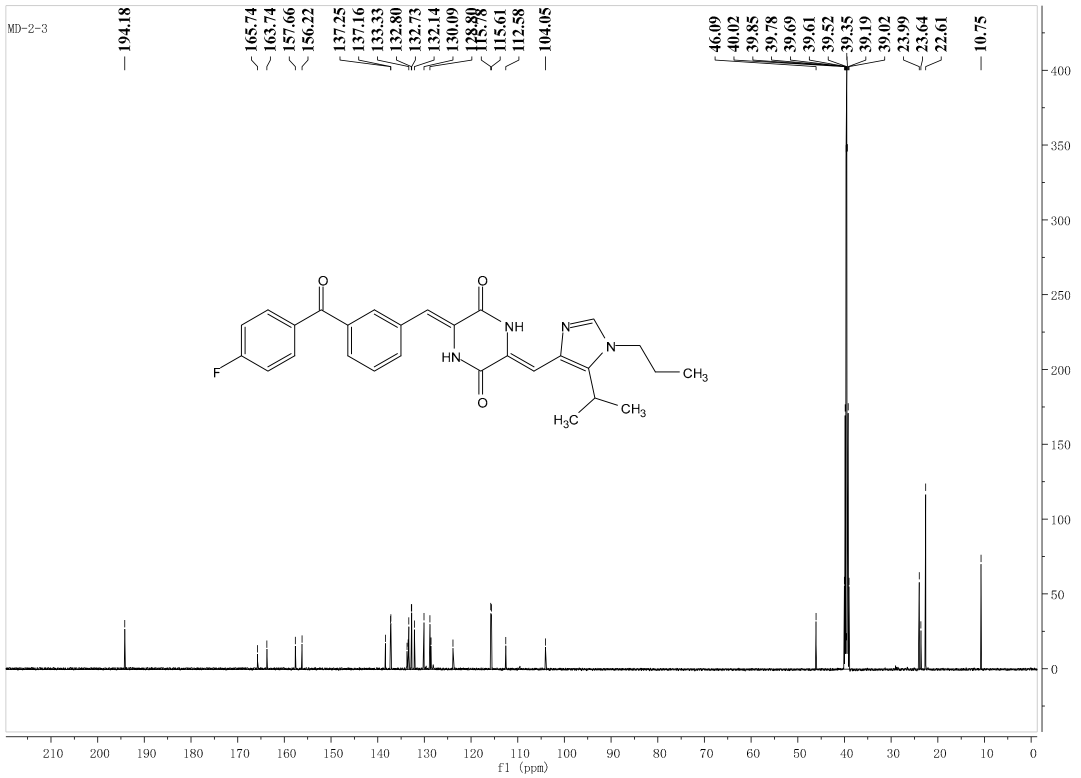

Figure S50. HRMS spectrum for **15i** (MeOH)

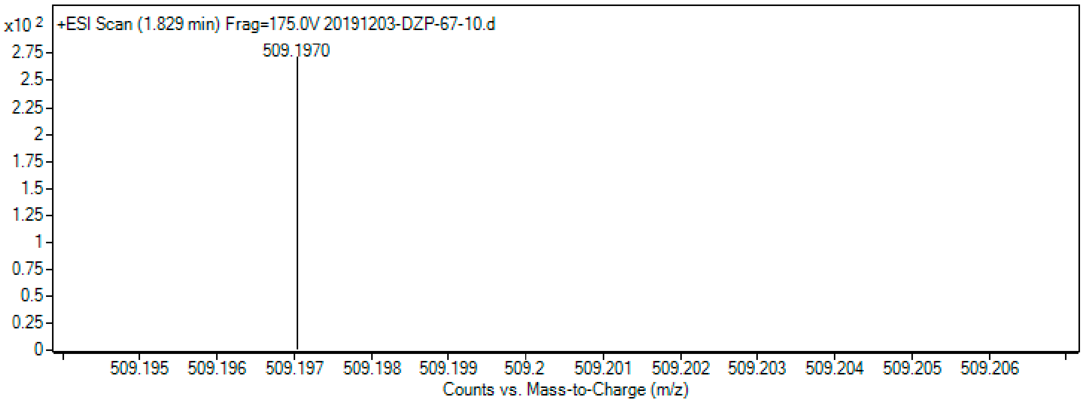

Figure S51. ^{1}H NMR spectrum for **15j** (500 MHz, DMSO-d_6)

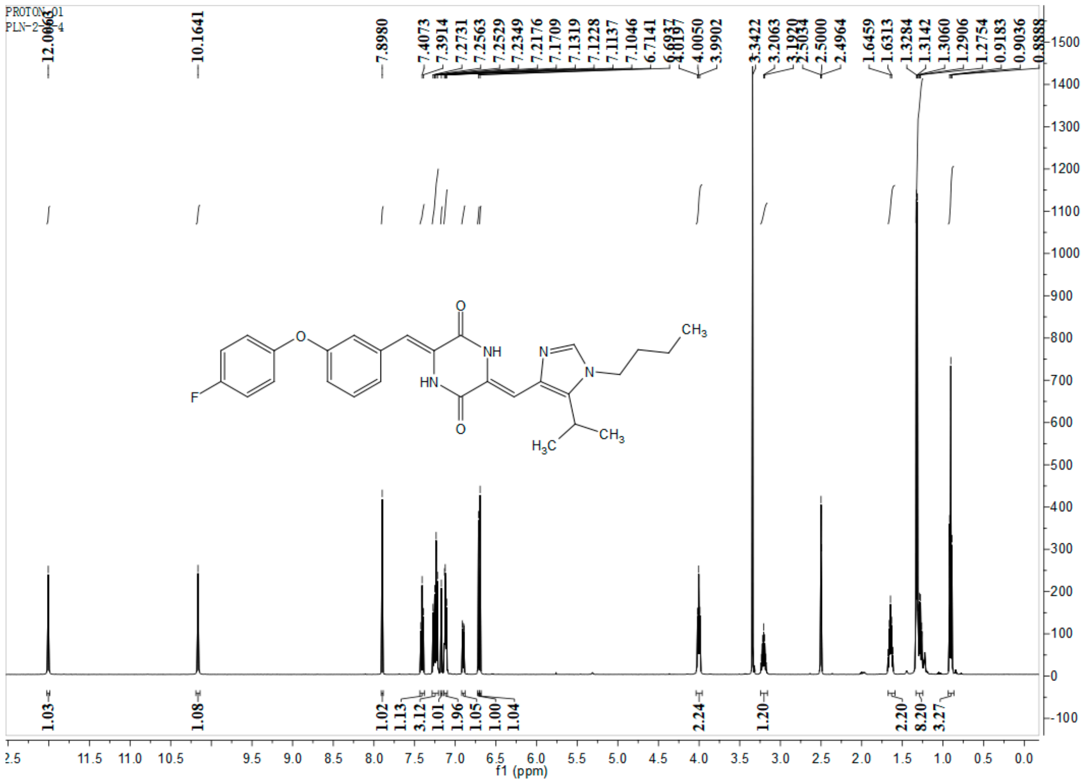

Figure S52. ^{13}C NMR spectrum for **15j** (125 MHz, DMSO-d_6)

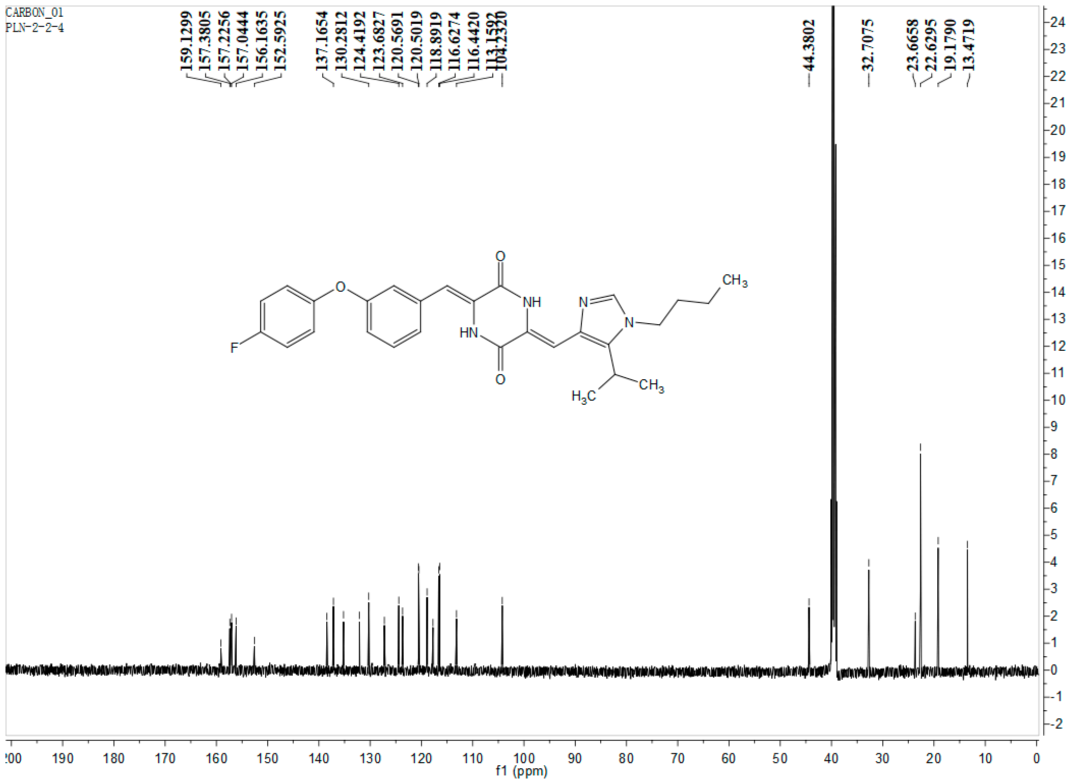

Figure S53. HRMS spectrum for **15j** (MeOH)

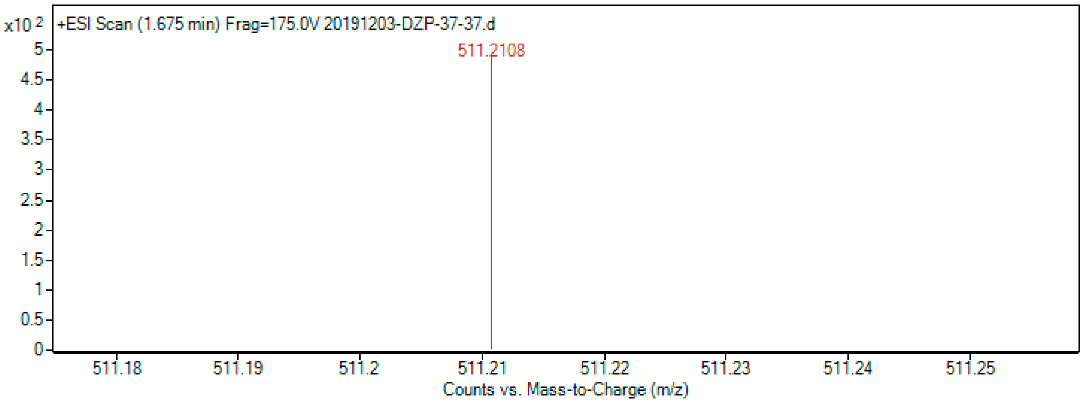

Figure S54. ^{1}H NMR spectrum for **15k** (500 MHz, DMSO-d_6)

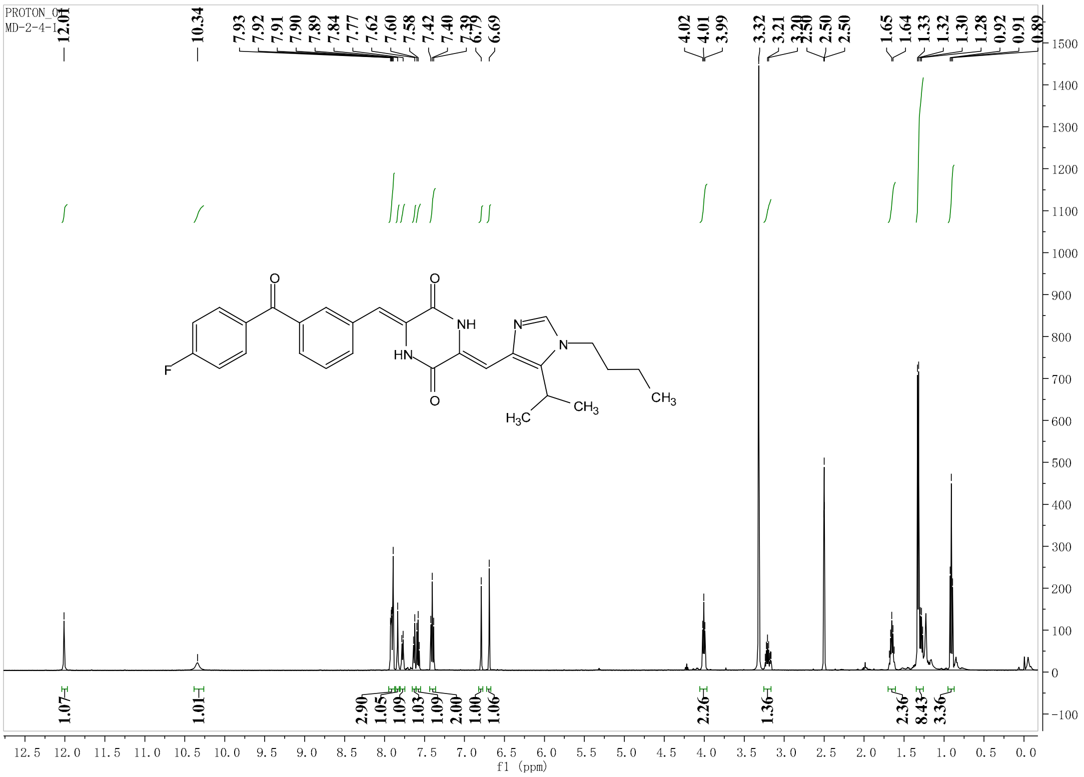

Figure S55. ^{13}C NMR spectrum for **15k** (125 MHz, DMSO-d_6)

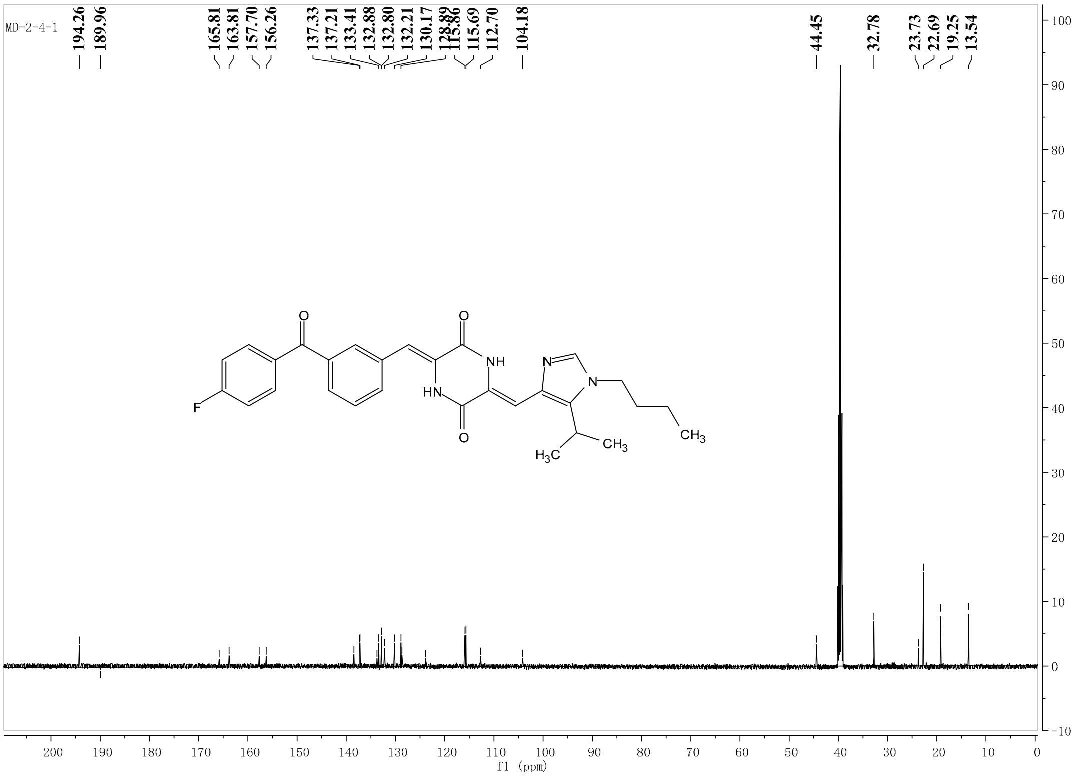

Figure S56. HRMS spectrum for **15k** (MeOH)

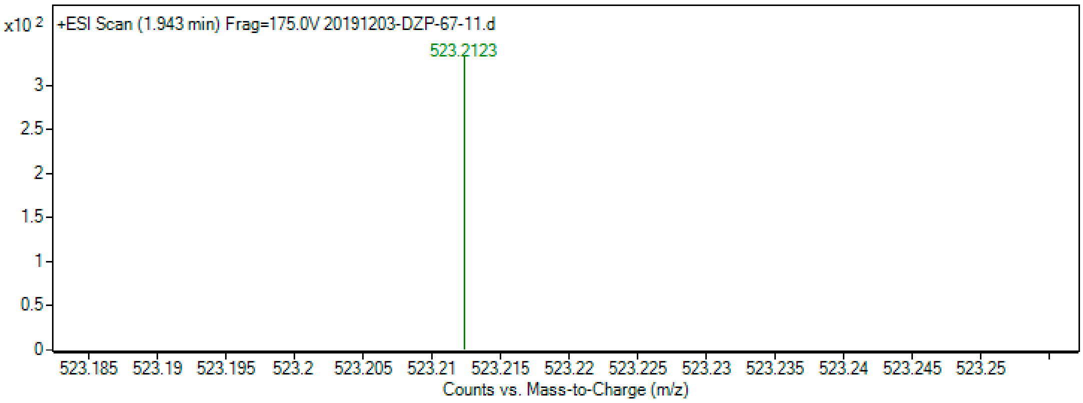

Figure S57. ^{1}H NMR spectrum for **15l** (500 MHz, DMSO-d_6)

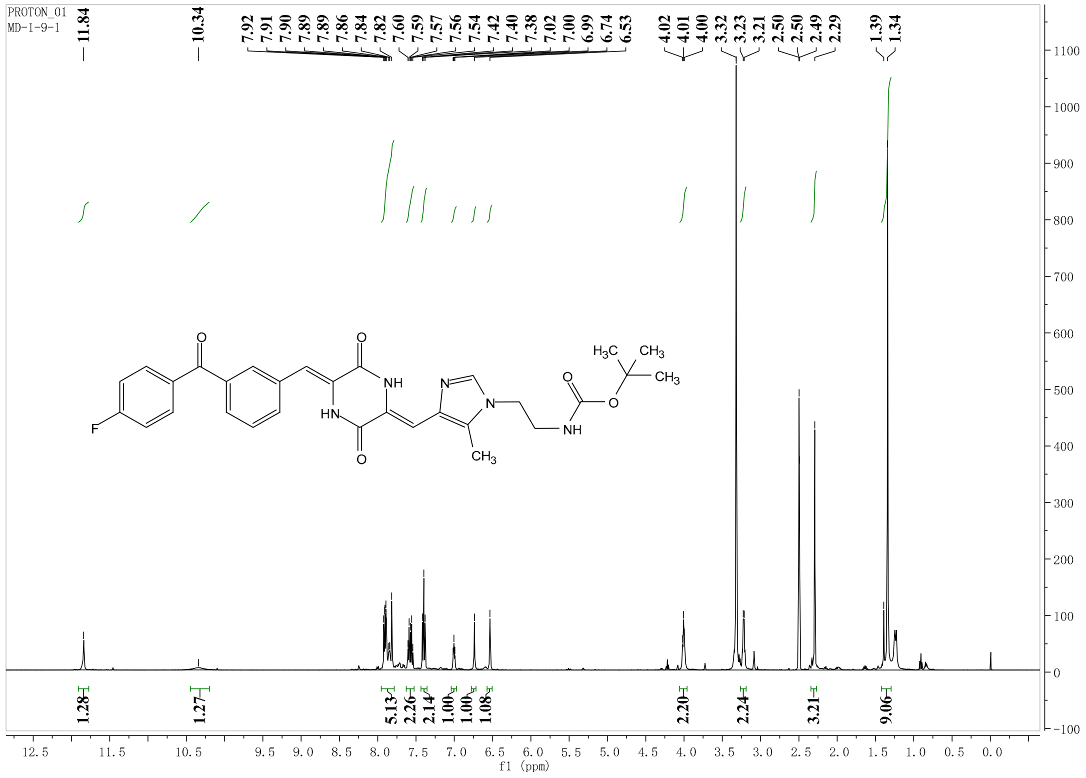

Figure S58. ^{13}C NMR spectrum for **15l** (125 MHz, DMSO-d_6)

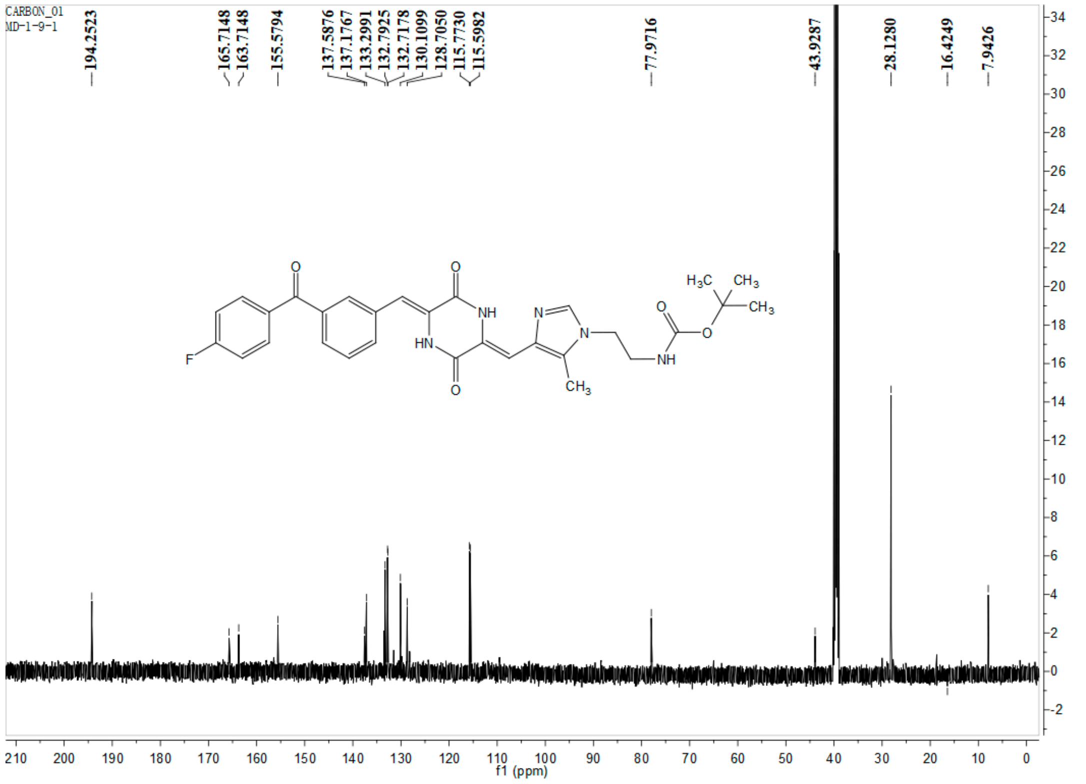

Figure S59. HRMS spectrum for **15l** (MeOH)

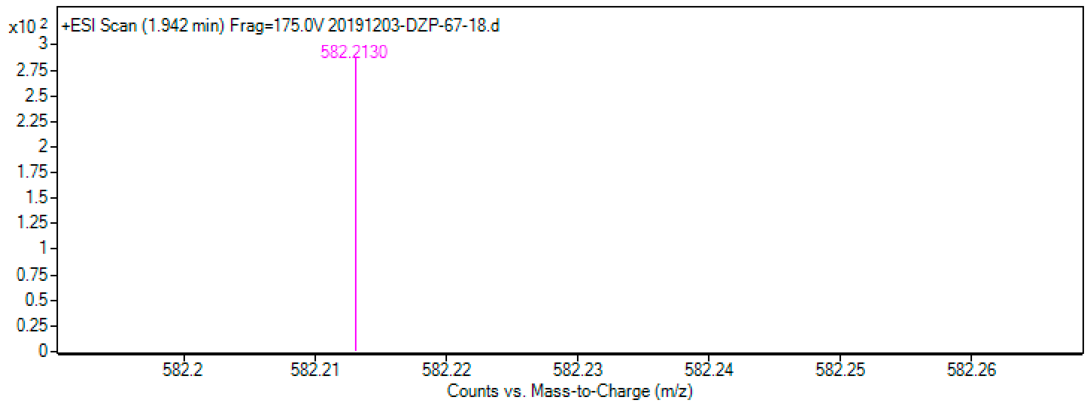

Figure S60. ^{1}H NMR spectrum for **15m** (500 MHz, DMSO-*d*6)

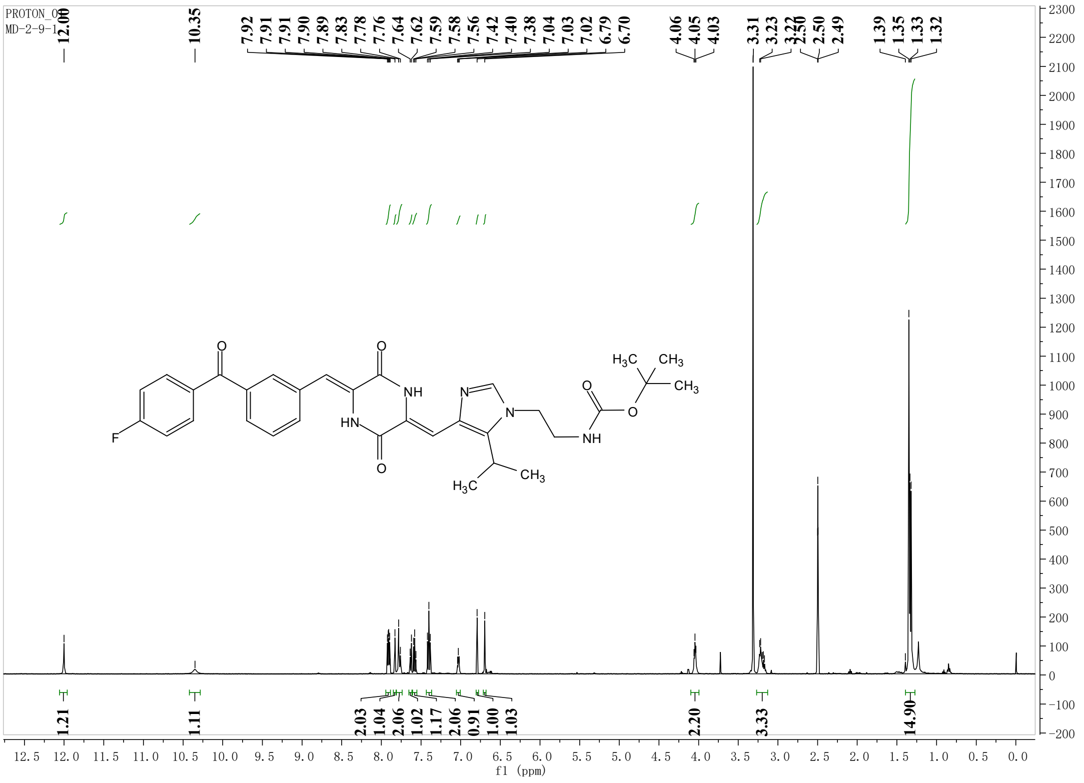

Figure S61. ^{13}C NMR spectrum for **15m** (125 MHz, DMSO-*d*6)

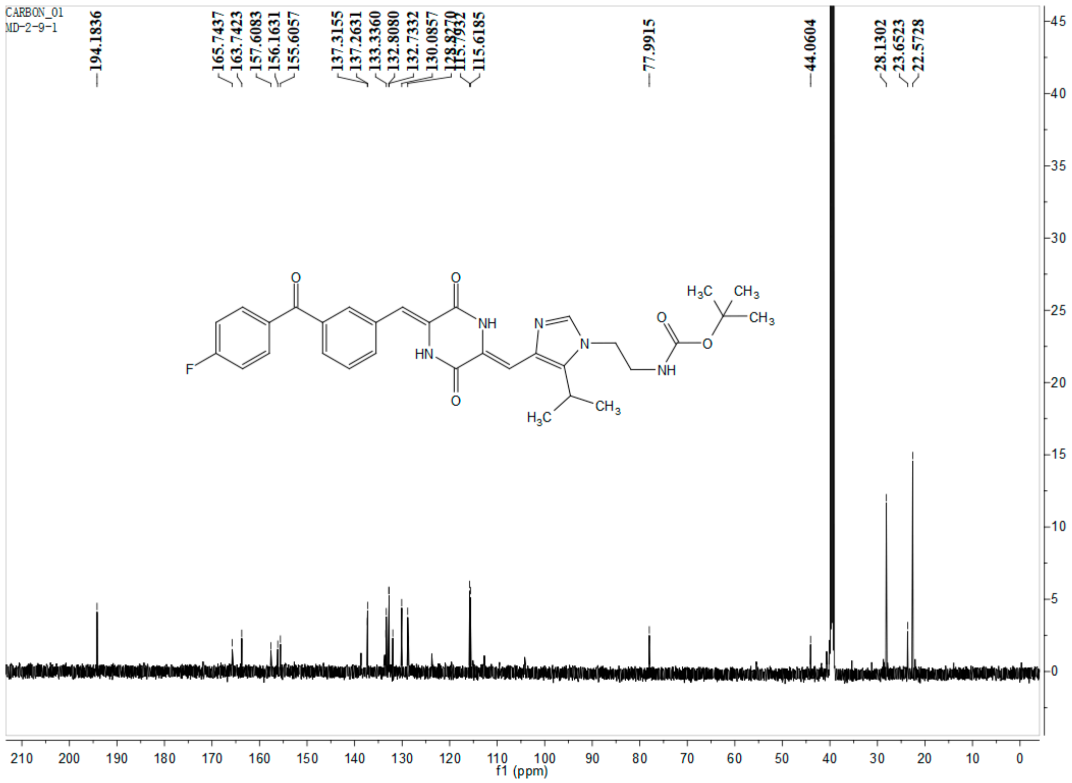

Figure S62. HRMS spectrum for **15m** (MeOH)

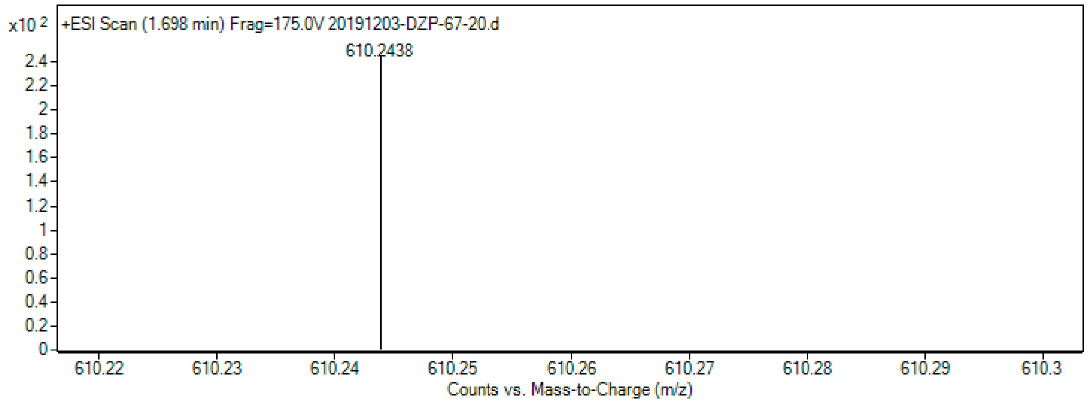

Figure S63. ^{1}H NMR spectrum for **15n** (500 MHz, DMSO-*d*6)

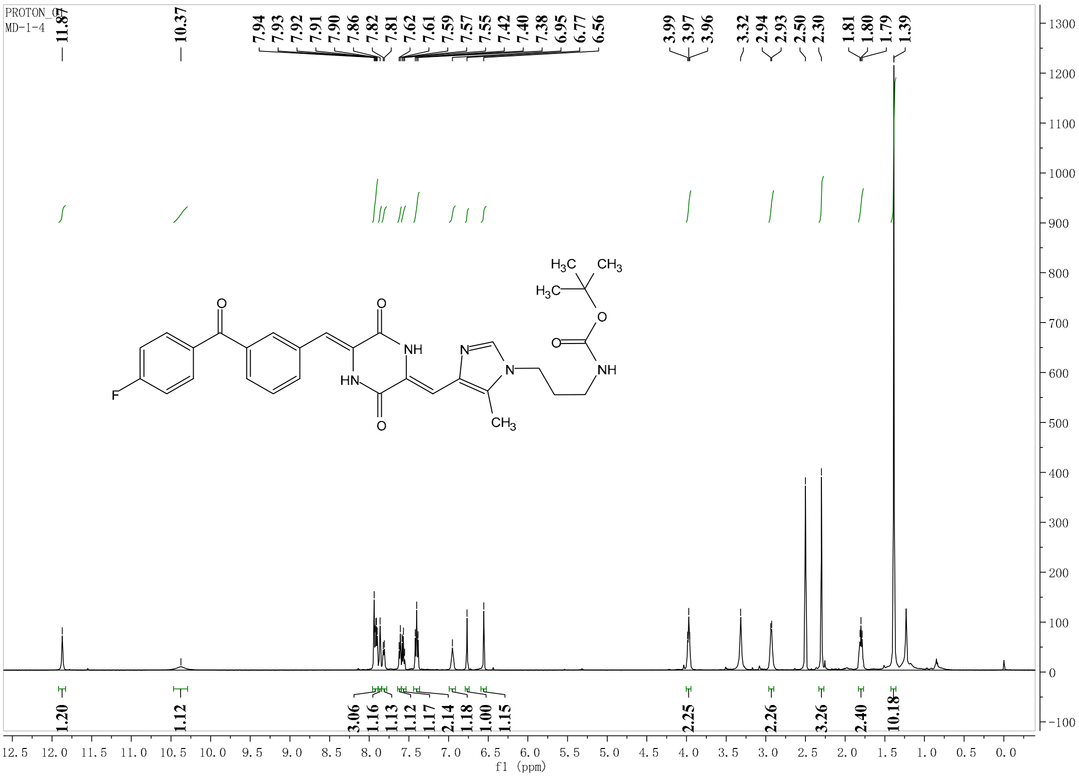

Figure S64. ^{13}C NMR spectrum for **15n** (125 MHz, DMSO-*d*6)

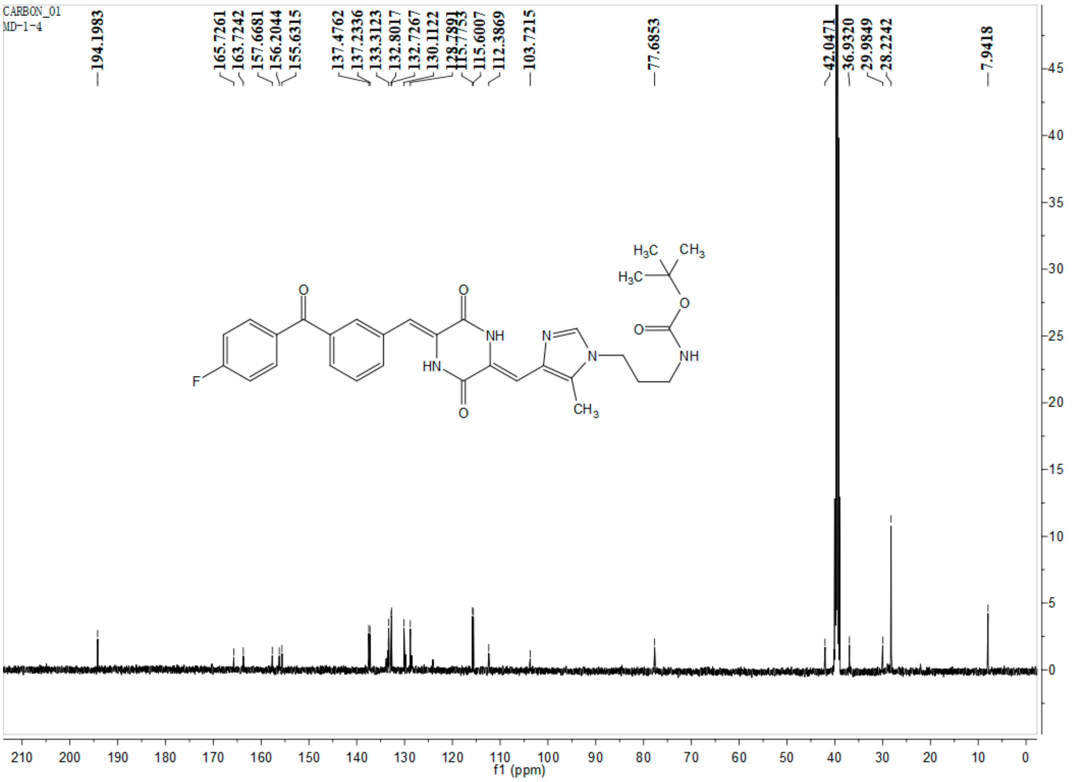

Figure S65. HRMS spectrum for **15n** (MeOH)

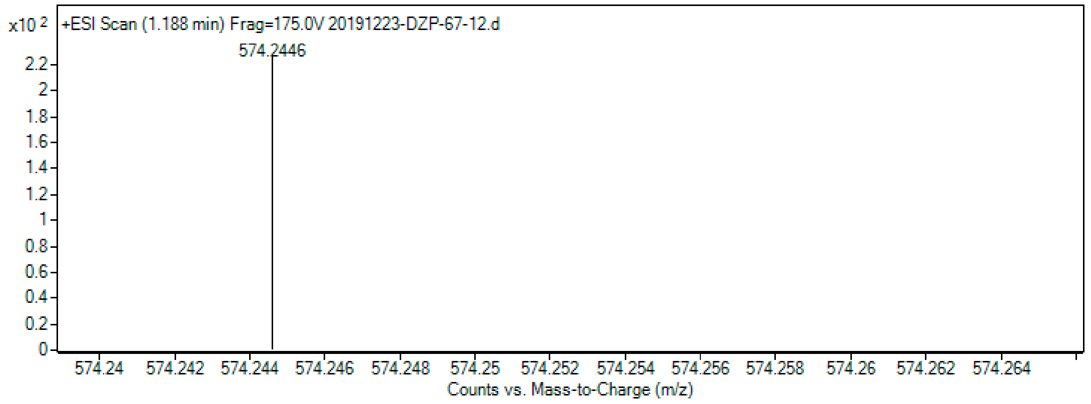

Figure S66. ^{1}H NMR spectrum for **15o** (500 MHz, DMSO-d_6)

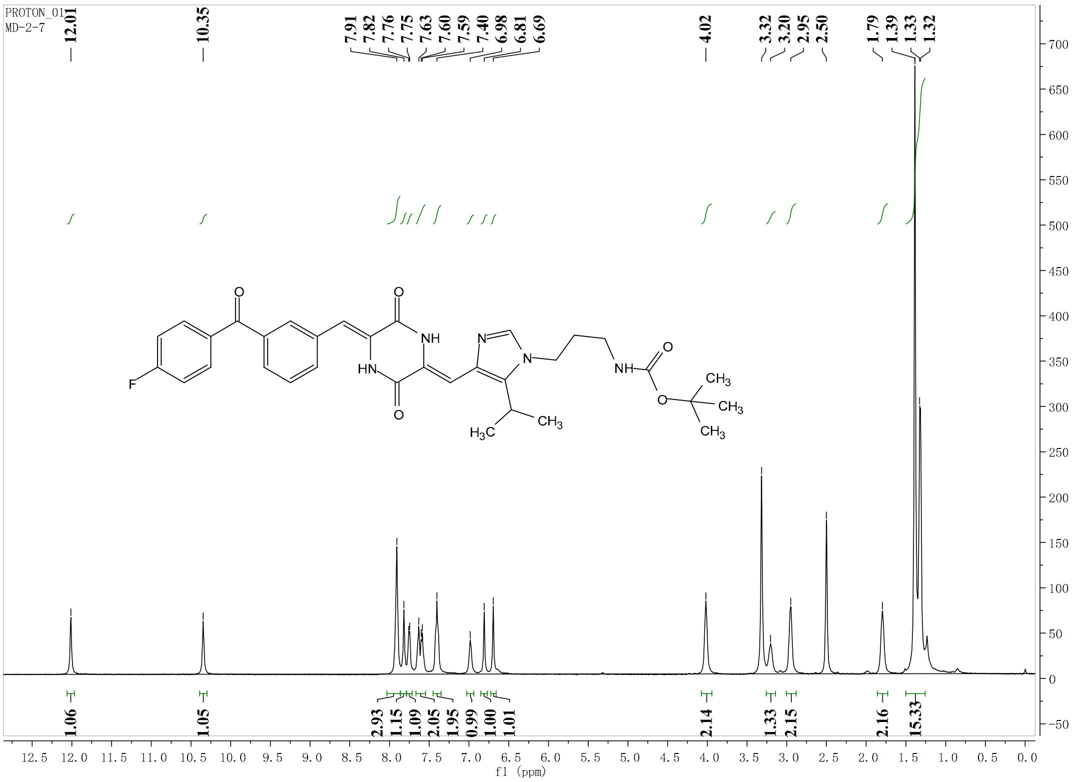

Figure S67. ^{13}C NMR spectrum for **15o** (125 MHz, DMSO-d_6)

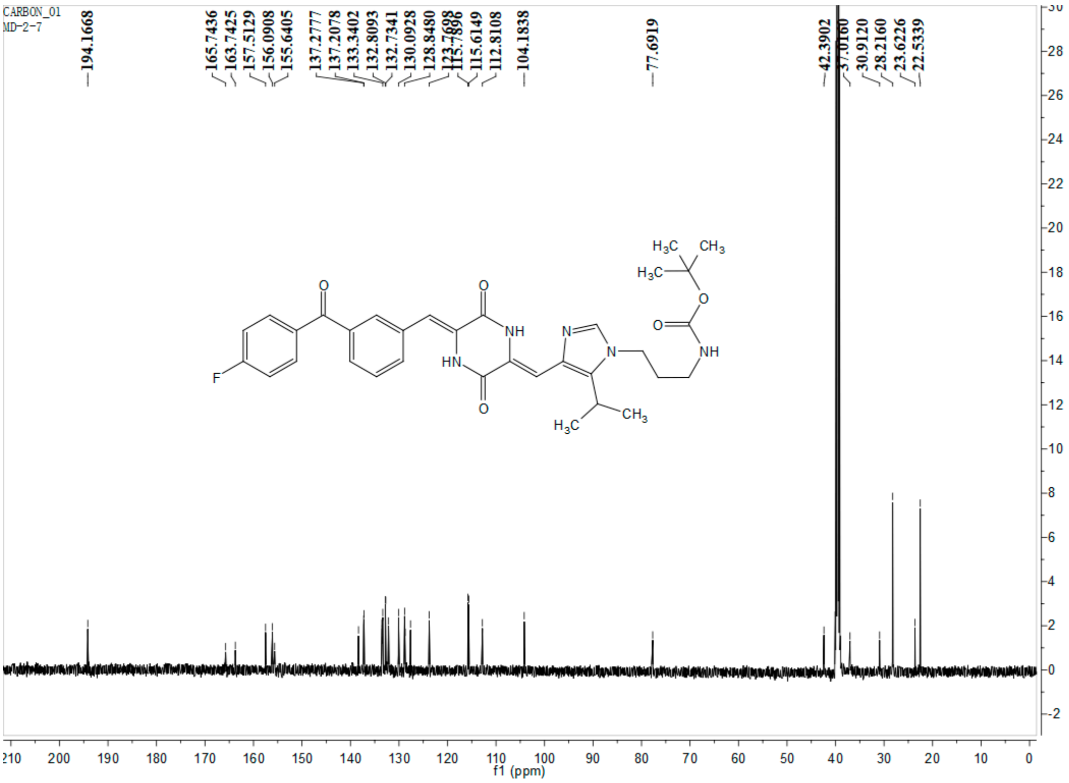

Figure S68. HRMS spectrum for **15o** (MeOH)

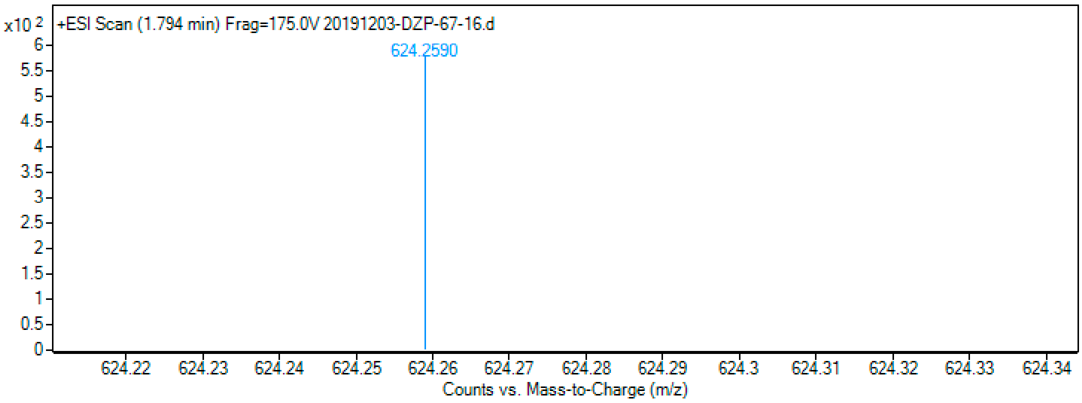

Figure S69. ^{1}H NMR spectrum for **15p** (500 MHz, DMSO-d_6)

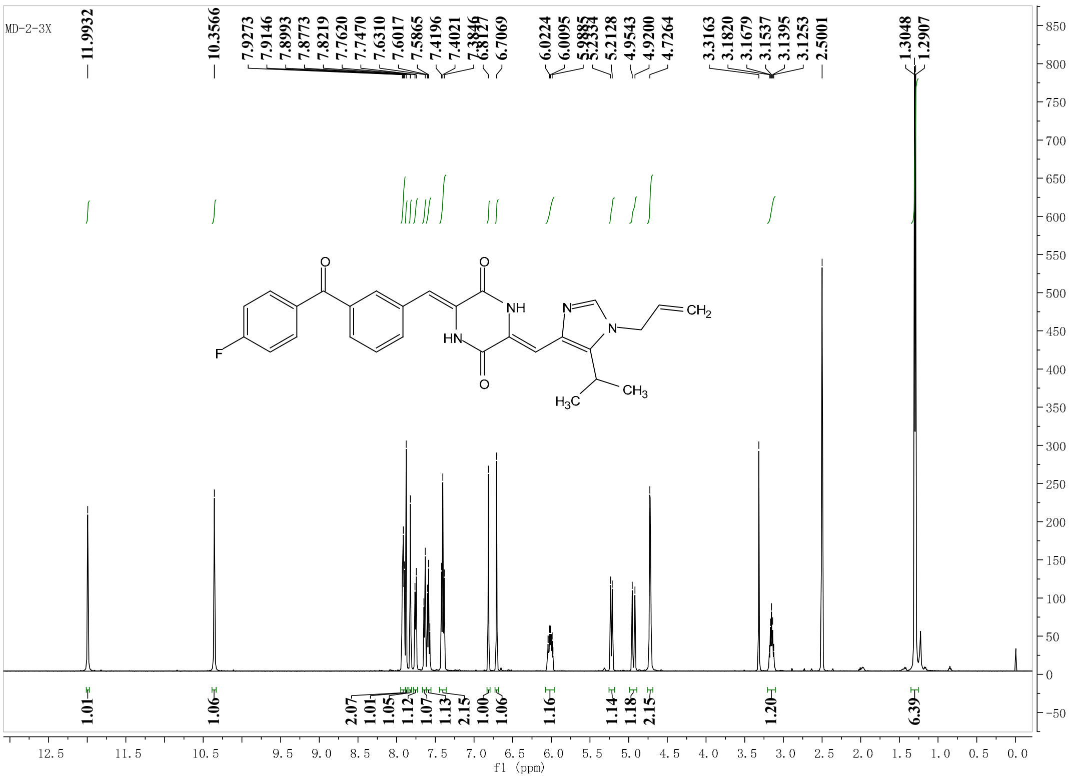

Figure S70. ^{13}C NMR spectrum for **15p** (125 MHz, DMSO-d_6)

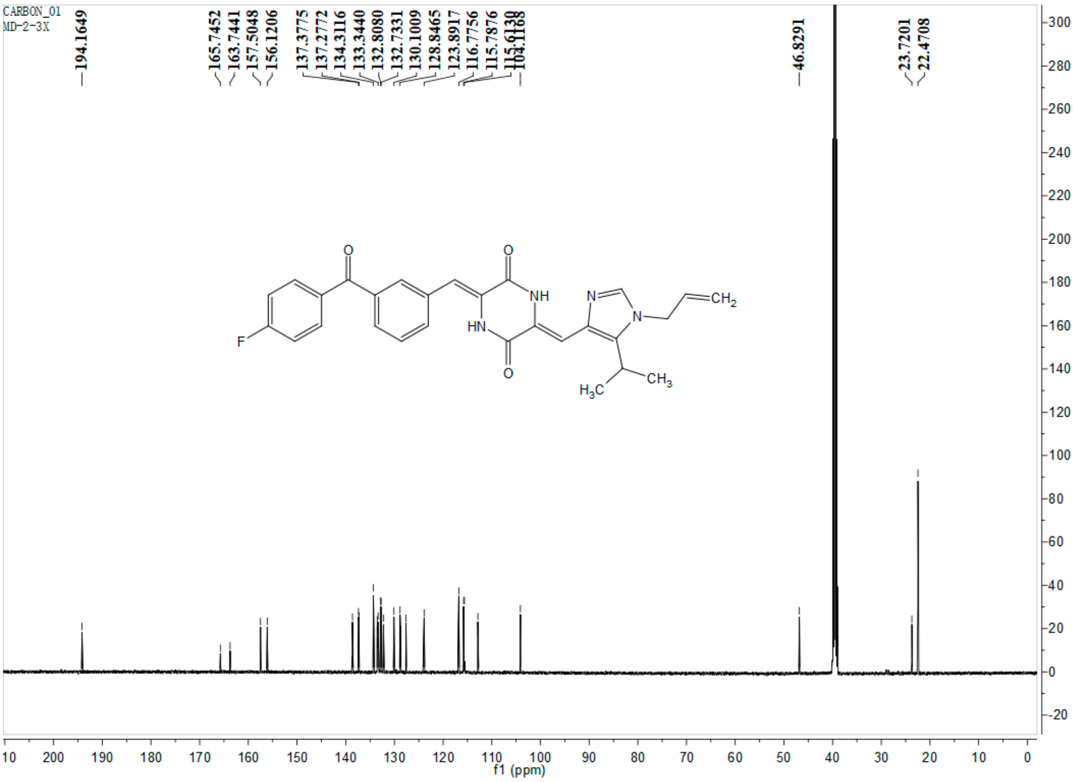

Figure S71. HRMS spectrum for **15p** (MeOH)

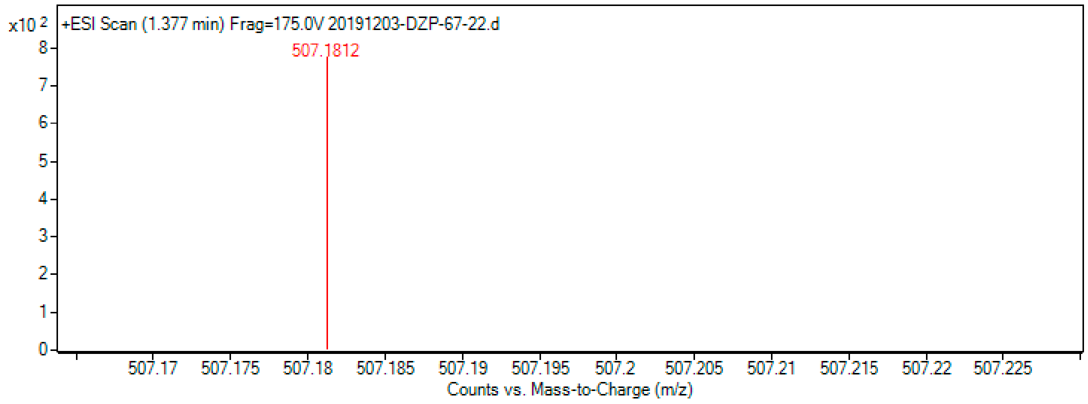

Figure S72. ^{1}H NMR spectrum for **15q** (500 MHz, DMSO-d_6)

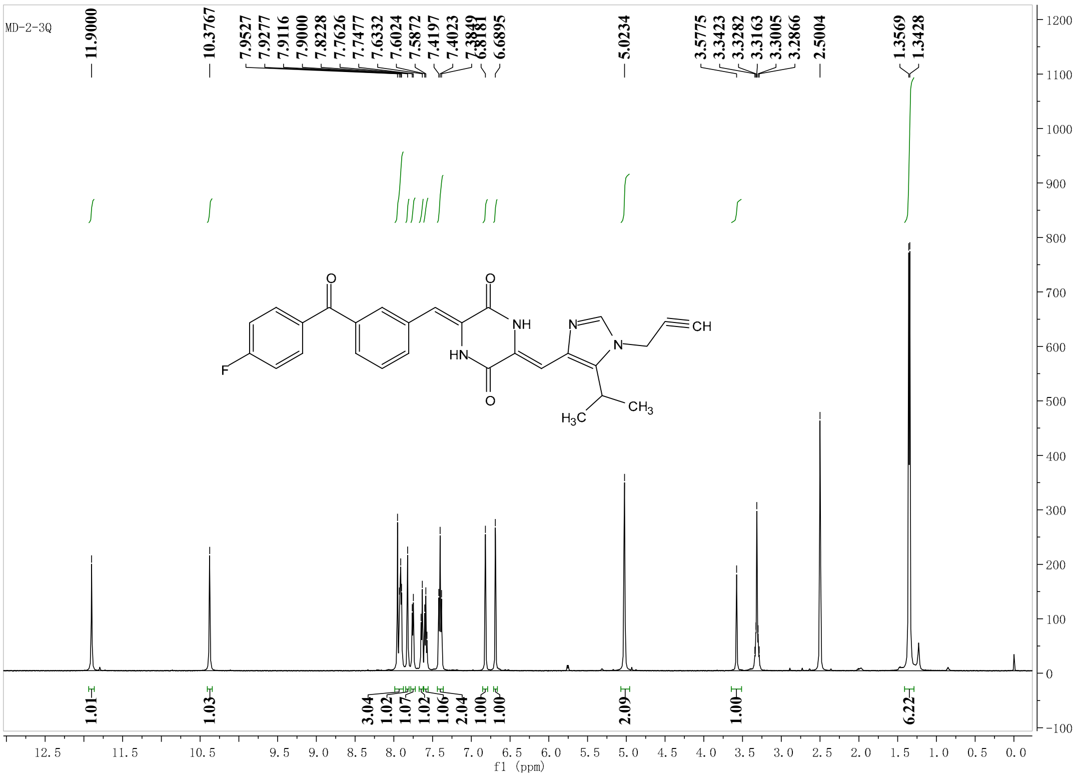

Figure S73. ^{13}C NMR spectrum for **15q** (125 MHz, DMSO-d_6)

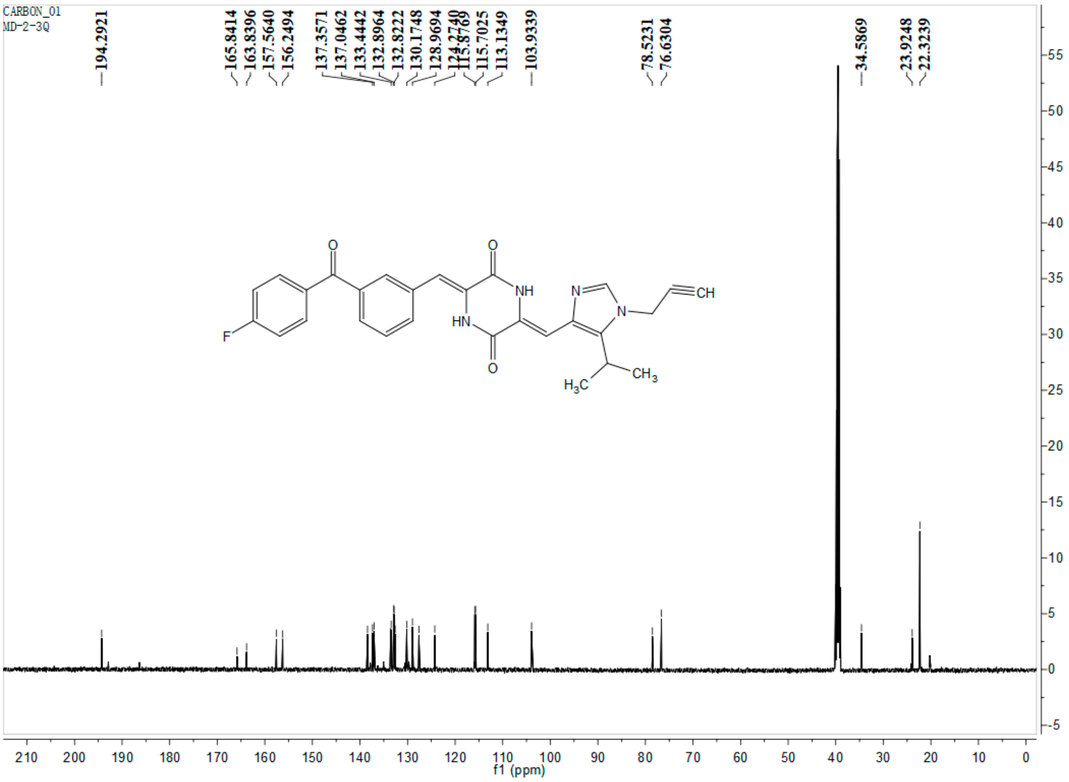

Figure S74. HRMS spectrum for **15q** (MeOH)

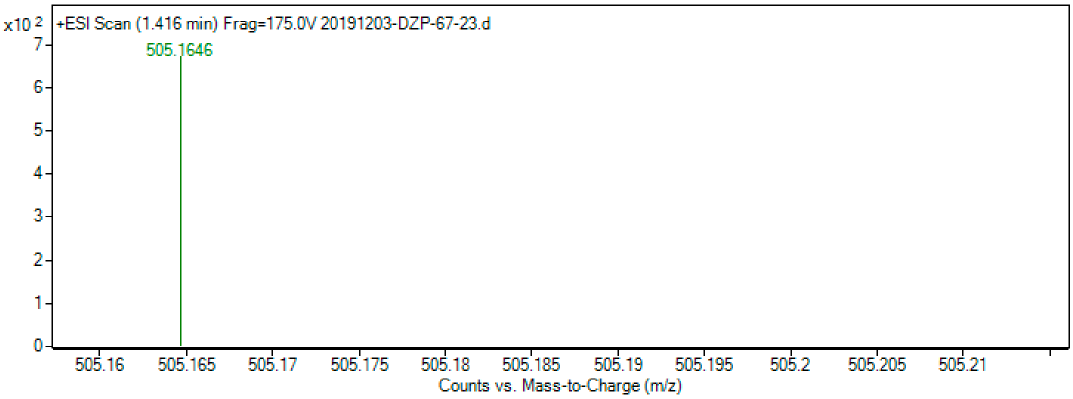

Figure S75. ^{1}H NMR spectrum for **16a** (500 MHz, DMSO-d_6)

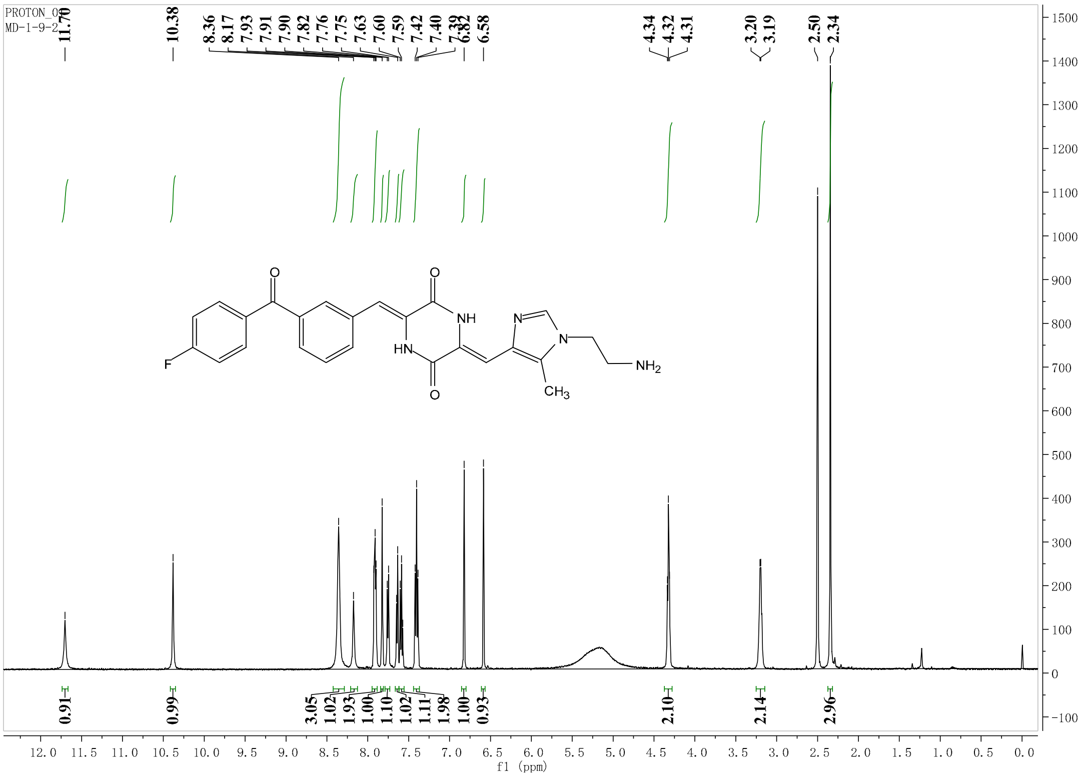

Figure S76. ^{13}C NMR spectrum for **16a** (125 MHz, DMSO-d_6)

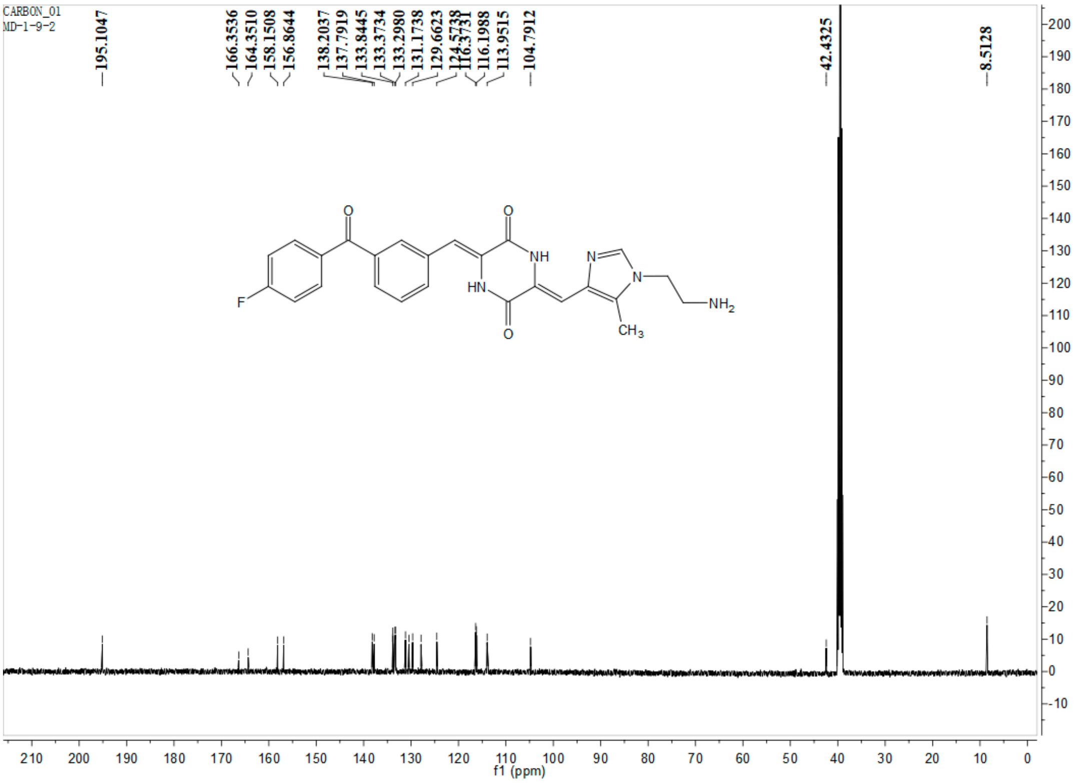

Figure S77. HRMS spectrum for **16a** (MeOH)

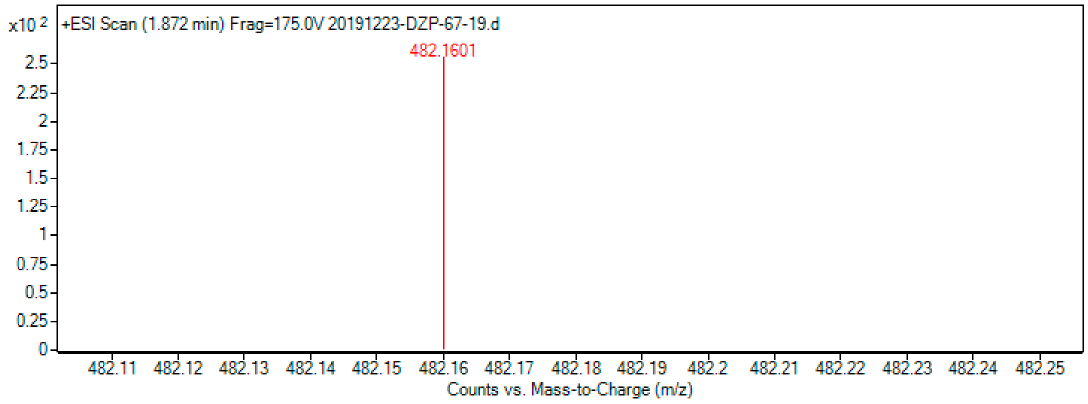

Figure S78. ^{1}H NMR spectrum for **16b** (500 MHz, DMSO-d_6)

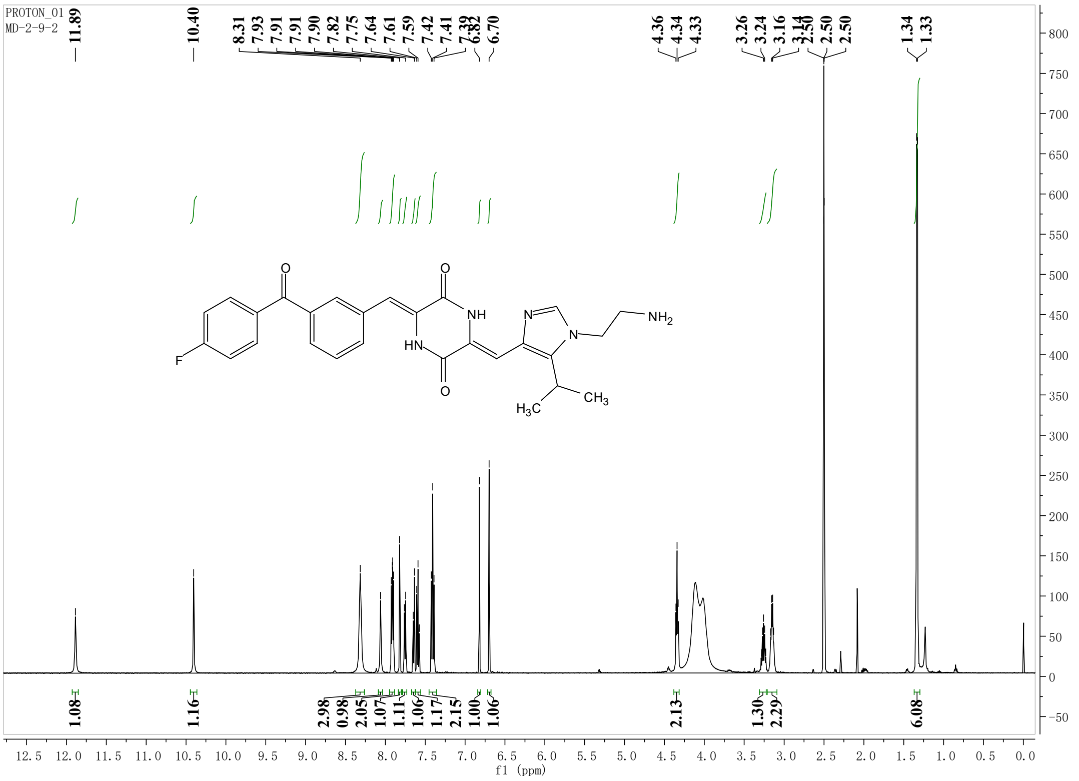

Figure S79. ^{13}C NMR spectrum for **16b** (125 MHz, DMSO-d_6)

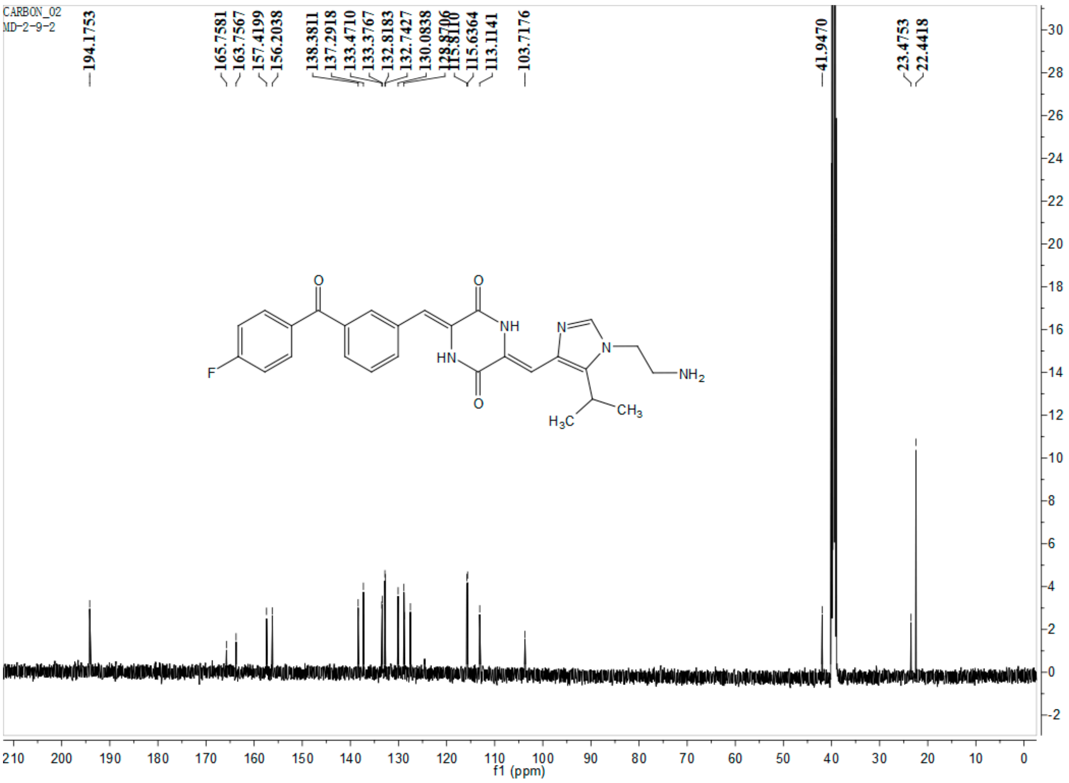

Figure S80. HRMS spectrum for **16b** (MeOH)

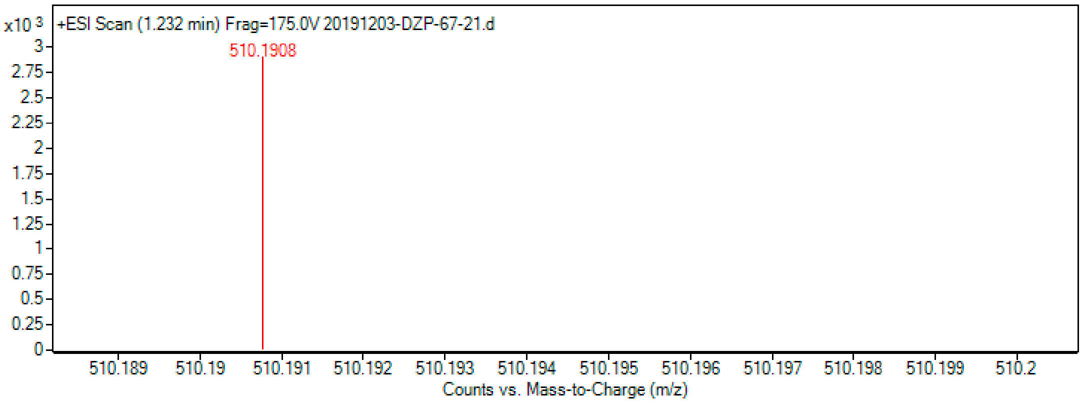

Figure S81. ^{1}H NMR spectrum for **16c** (500 MHz, DMSO-d_6)

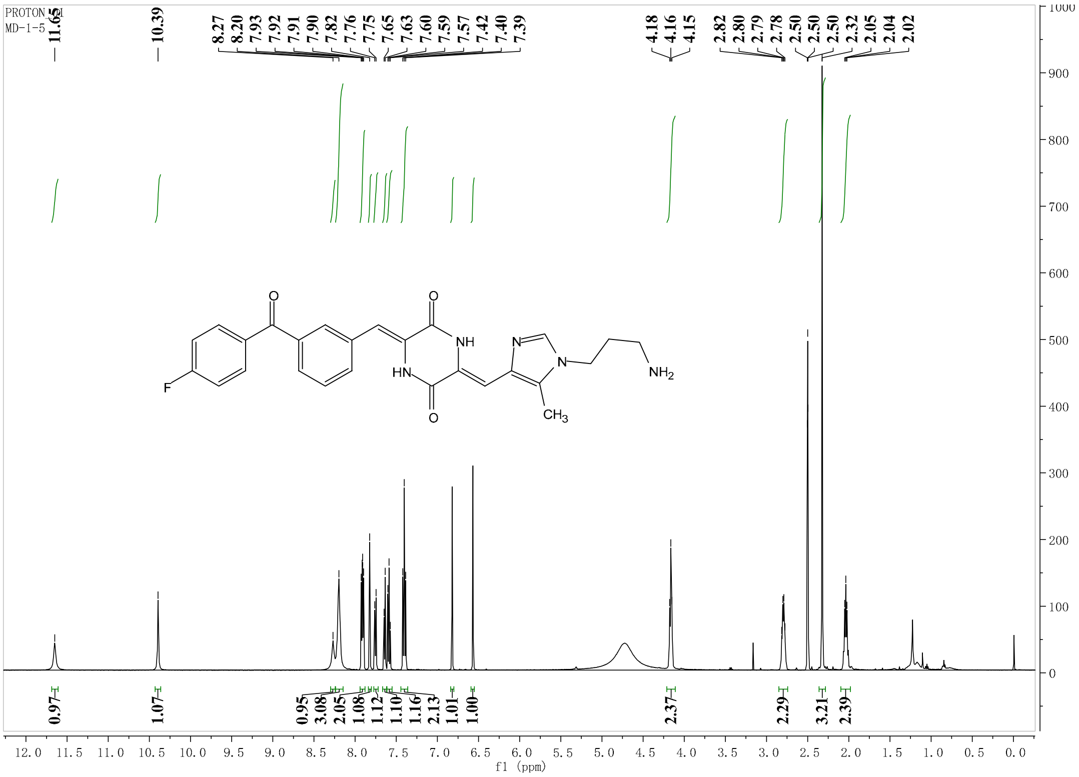

Figure S82. ^{13}C NMR spectrum for **16c** (125 MHz, DMSO-d_6)

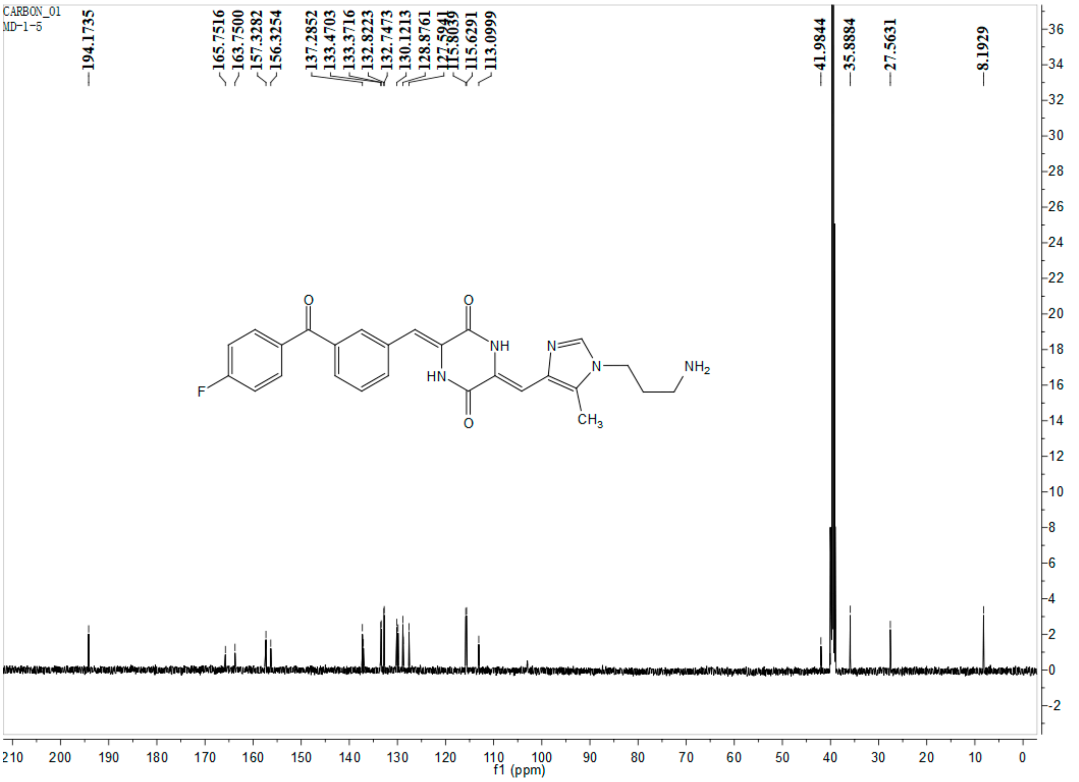

Figure S83. HRMS spectrum for **16c** (MeOH)

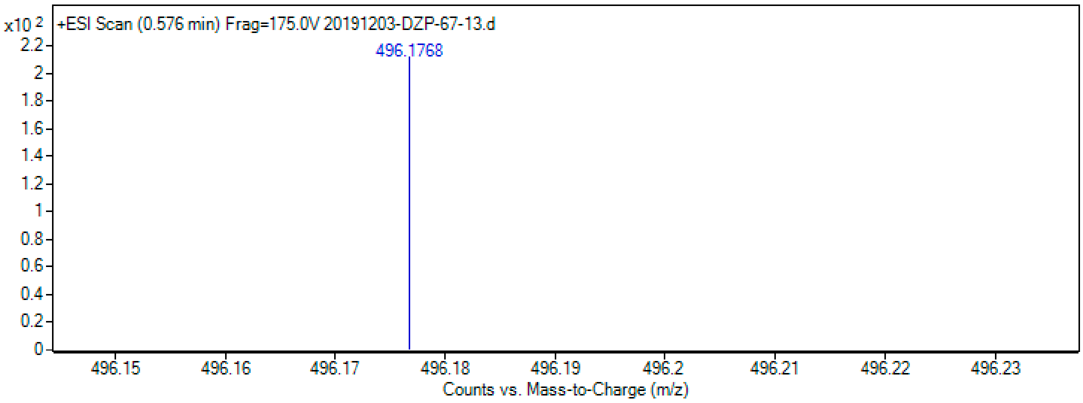

Figure S84. ^{1}H NMR spectrum for **16d** (500 MHz, DMSO-d_6)

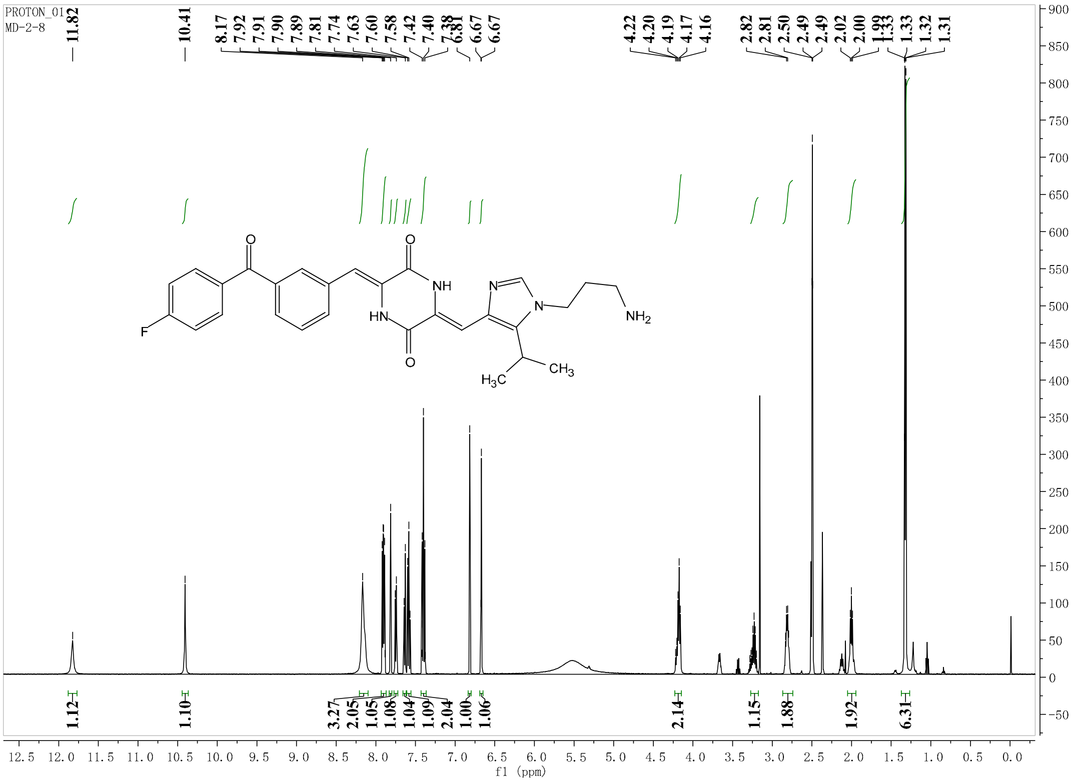

Figure S85. ^{13}C NMR spectrum for **16d** (125 MHz, DMSO-d_6)

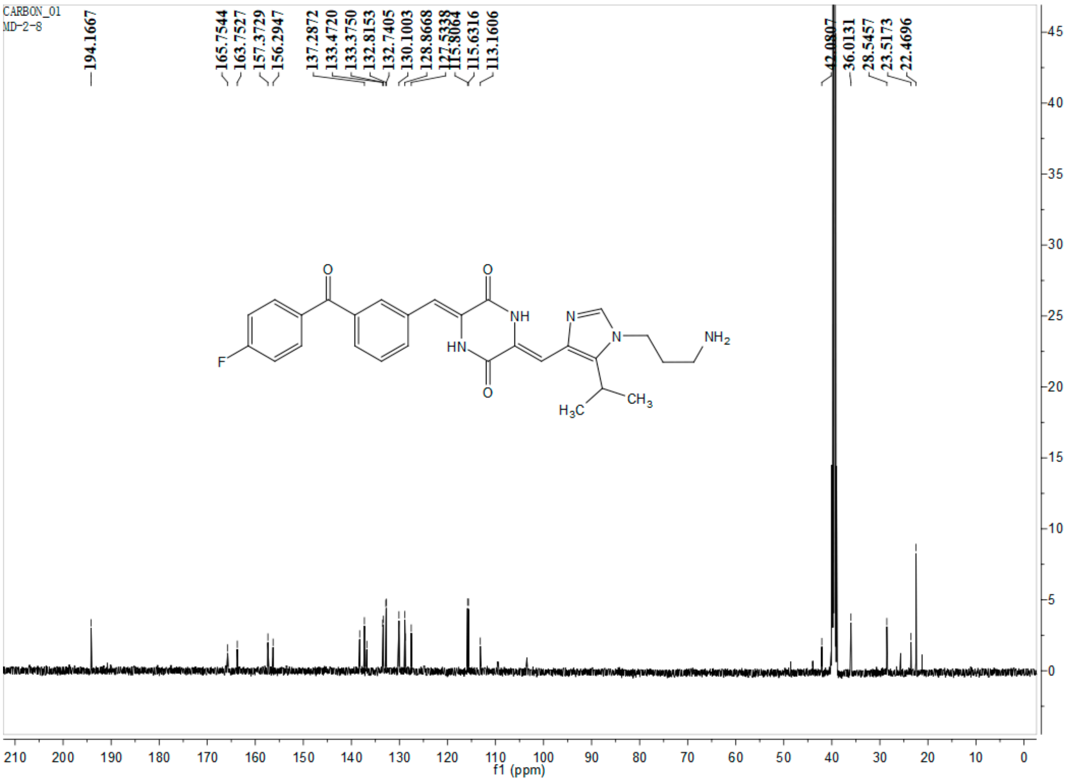

Figure S86. HRMS spectrum for **16d** (MeOH)

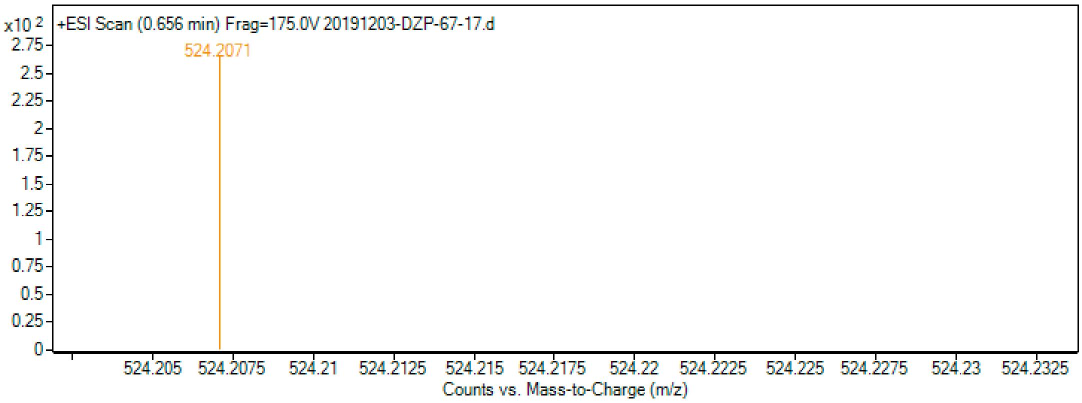

Supplement: Supplementary file 1 [file marinedrugs-20-00752-s001.zip › marinedrugs-2030600-supplementary.pdf]
